# The microbiota conditions a gut milieu that selects for wild-type *Salmonella* Typhimurium virulence

**DOI:** 10.1371/journal.pbio.3002253

**Published:** 2023-08-31

**Authors:** Ersin Gül, Erik Bakkeren, Guillem Salazar, Yves Steiger, Andrew Abi Younes, Melanie Clerc, Philipp Christen, Stefan A. Fattinger, Bidong D. Nguyen, Patrick Kiefer, Emma Slack, Martin Ackermann, Julia A. Vorholt, Shinichi Sunagawa, Médéric Diard, Wolf-Dietrich Hardt

**Affiliations:** 1 Institute of Microbiology, Department of Biology, ETH Zurich, Zurich, Switzerland; 2 Department of Biology, University of Oxford, Oxford, United Kingdom; 3 Department of Biochemistry, University of Oxford, Oxford, United Kingdom; 4 Institute of Microbiology and Swiss Institute of Bioinformatics, Department of Biology, ETH Zürich, Zürich, Switzerland; 5 Science for Life Laboratory, Department of Medical Biochemistry and Microbiology, Uppsala University, Uppsala, Sweden; 6 Institute for Food, Nutrition and Health, ETH Zürich, Zürich, Switzerland; 7 Institute of Biogeochemistry and Pollutant Dynamics, Department of Environmental Systems Science, ETH Zurich, Zurich, Switzerland; 8 Department of Environmental Microbiology, Eawag, Duebendorf, Switzerland; 9 Biozentrum, University of Basel, Basel, Switzerland; 10 Botnar Research Centre for Child Health, Basel, Switzerland; Georgia Institute of Technology, UNITED STATES

## Abstract

*Salmonella* Typhimurium elicits gut inflammation by the costly expression of HilD-controlled virulence factors. This inflammation alleviates colonization resistance (CR) mediated by the microbiota and thereby promotes pathogen blooms. However, the inflamed gut-milieu can also select for *hilD* mutants, which cannot elicit or maintain inflammation, therefore causing a loss of the pathogen’s virulence. This raises the question of which conditions support the maintenance of virulence in *S*. Typhimurium. Indeed, it remains unclear why the wild-type *hilD* allele is dominant among natural isolates. Here, we show that microbiota transfer from uninfected or recovered hosts leads to rapid clearance of *hilD* mutants that feature attenuated virulence, and thereby contributes to the preservation of the virulent *S*. Typhimurium genotype. Using mouse models featuring a range of microbiota compositions and antibiotic- or inflammation-inflicted microbiota disruptions, we found that irreversible disruption of the microbiota leads to the accumulation of *hilD* mutants. In contrast, in models with a transient microbiota disruption, selection for *hilD* mutants was prevented by the regrowing microbiota community dominated by Lachnospirales and Oscillospirales. Strikingly, even after an irreversible microbiota disruption, microbiota transfer from uninfected donors prevented the rise of *hilD* mutants. Our results establish that robust *S*. Typhimurium gut colonization hinges on optimizing its manipulation of the host: A transient and tempered microbiota perturbation is favorable for the pathogen to both flourish in the inflamed gut and also minimize loss of virulence. Moreover, besides conferring CR, the microbiota may have the additional consequence of maintaining costly enteropathogen virulence mechanisms.

## Abstract 2

The costly expression of virulence factors by the enteropathogen Salmonella Typhimurium elicits gut inflammation which in turn alleviates microbiota-mediated colonization resistance and promotes pathogen blooms. This comprehensive study explains the tight connection between microbiota complexity and evolution of Salmonella virulence.

**Highlights**

- In wild-type *S*. Typhimurium infections, the trade-off between virulence costs and benefits is strongly affected by the microbiota.
- Wild-type virulence in antibiotic-treated mice can disrupt microbiota irreversibly, which selects for long-term gut colonization by attenuated *hilD* mutants.
- Microbiota transfer can prevent selection for *hilD* mutants in wild-type *S*. Typhimurium infections.
- Microbiota transfer can displace *hilD*-dominated *Salmonella* populations from the gut lumen.

## Introduction

The gut is colonized with a diverse gut microbiota, which provides numerous beneficial functions including colonization resistance (CR) [1,2]. CR describes the microbiota’s ability to block the growth of pathogens in the gut lumen [3] by diverse mechanisms (reviewed in detail elsewhere; [4]). To overcome CR, gut pathogens can deploy virulence factors that elicit intestinal disease often characterized by a pronounced immune response (i.e., inflammation) that alters the gut-luminal milieu and the architecture of the gut tissue to the detriment of the host (i.e., enteropathy). Therefore, a pathogen’s virulence is associated with a trade-off between reducing host fitness (via intestinal disease) but enhancing its own gut luminal growth and subsequent transmission by depleting competing species in the microbiota [5]. By extension, the tripartite interaction between the host, the pathogen, and the microbiota is thought to influence the evolution of enteropathogen virulence, although the selective environment appears to be highly context dependent [6–8]. While some experimental work has assessed virulence evolution in simplified model systems or invertebrate hosts, studies of virulence evolution in the mammalian gut have remained scarce [7–9]. The eukaryotic pathogen *Candida albicans* is a notable exception. Elegant evolution experiments established that the gut microbiota select for wild-type *C*. *albicans* virulence while in germ-free mice, virulence-attenuated mutants featuring hyphal growth defects were selected for [10,11]. However, it remains unclear if the microbiota would affect virulence evolution of enteropathogenic bacteria in a similar fashion.

Infection biology has identified numerous toxins, injected effector proteins and innate immune stimuli of enteric pathogens such as *Salmonella enterica* serovar Typhimurium (*S*.Tm), *Citrobacter rodentium*, *Vibrio cholerae*, or *Clostridioides difficile*, which engage the host, elicit disease, alleviate CR, and thereby promote gut-luminal growth [12–16]. Promoting gut colonization is thought to rationalize the existence of virulence in these enteropathogens, yet only few studies have leveraged experimental evolution to probe the effects of the microbiota on virulence evolution in enteropathogenic bacteria.

*S*. Typhimurium expresses flagella, adhesins and most notably 2 type III secretion systems (TTSS) to approach the gut surface, invade the gut tissue, and elicit gut inflammation [17]. The TTSS-1 facilitates invasion into the gut epithelium and TTSS-2 enhances survival and growth within host tissues [18–20]. Virulence effectors secreted by the TTSS-2 system allow the pathogen to survive intracellularly in the *Salmonella* containing vacuole by interfering with the endocytic trafficking and by conferring survival from NADPH-oxidase-dependent killing [21]. Therefore, wild-type infections can drastically decrease host lifespan, such as in susceptible mice like C57BL/6 [18]. In streptomycin pretreated mice, wild-type *S*. Typhimurium blooms to high densities of ≈10^9^ CFU/g stool in the gut lumen, elicits gut inflammation (as measured by histopathology, cytokine gene expression profiling, or ELISA for the gut inflammation marker lipocalin-2) and thereby suppresses microbiota regrowth [12,19]. *S*. Typhimurium mutants lacking functional TTSS-1 and TTSS-2 machineries (like deletion mutants lacking the structural genes *invG* and *ssaV*) fail to trigger enteropathy and are displaced by regrowing gut microbiota within 4 days [12]. *S*. Typhimurium mutants that retain either TTSS-1 (with dysfunctional TTSS-2; like *ssaV*) or TTSS-2 (with dysfunctional TTSS-1; like *invG*) elicit enteropathy which is milder compared to wild-type *S*. Typhimurium [20,22,23]. Such mutants also show reduced transmission as indicated by reduced fecal pathogen loads in various mouse infection models [12,20,24,25].

*S*. Typhimurium enteric virulence is tightly regulated by the master regulator HilD, which controls the genes encoding TTSS-1, TTSS-2, flagella, and the SiiE adhesin [26,27] (S1 Fig). Thereby, HilD controls gut tissue infection, gut luminal growth, and transmission of *S*. Typhimurium, while suppressing microbiota regrowth in antibiotic-pretreated mice [12,17,28–33]. The expression of HilD itself is embedded in a tightly controlled regulon that is critical for minimizing the fitness costs associated with the expression of *S*. Typhimurium virulence [29]. The signals for expression appear to be derived or controlled by both the microbiota and the host [34,35] (S1 Fig). For example, these signals provide environmental cues for controlled expression of *S*. Typhimurium virulence factors such that the associated costs occur only at those moments of the infection cycle when the respective virulence factors are needed. In antibiotic pretreated mice, this is well established for the costly expression of the virulence factors required for the invasion of the gut epithelium and triggering of gut inflammation (in particular for TTSS-1, flagella, and the SiiE adhesin). The HilD regulon then shuts them off after mucosa invasion once they are no longer needed [36]. When virulence is expressed, however, the fitness cost manifests in reduced growth rates and exacerbated envelope stress sensitivity of TTSS-1-expressing *S*. Typhimurium cells (as shown ex vivo; [37,38]). In fact, these costs are strong enough for the selection for *hilD* mutants over *hilD*-proficient strains during 1 infection cycle, as shown in a proof-of-principle study [29]. This suggested that the cost of HilD-controlled virulence is a critical factor in *Salmonella* virulence evolution. This previous work [29], however, was limited as it focused on *ssaV* mutants of *S*. Typhimurium in order to follow infections of C57BL/6 mice for up to 10 days without compromising host survival. While this work discovered regulatory mechanisms that slowed the selection for *hilD* mutants in vivo, it neglected to capture the full virulence of *S*. Typhimurium as it occurs in the wild. This is important as wild-type *S*. Typhimurium elicits a stronger form of enteric disease than the *ssaV* mutant [20,23,24]. Furthermore, while long-term infection experiments with wild-type *S*. Typhimurium have been performed, the evolutionary dynamics of the pathogen were not studied [12]. Therefore, how wild-type *S*. Typhimurium virulence evolves in the gut during the infection of a host remained unclear.

Recent studies discovered that HilD-regulon mutants are positively selected in a small fraction of natural *Salmonella* isolates (78 *hilD* nonsense mutants per 100,000 isolates) [39–41]. The frequency of *hilD*-deficient mutants featuring premature stop codons was ≈22-fold higher than expected for genes that are under no selection. Based on these data, Cherry suggested that *hilD*-proficient strains are favored in most relevant conditions and that the 78 *hilD* mutants point to the existence of some unidentified condition where *hilD*-deficient mutants are selected for. These observations further supported that the pathogen faces fundamental trade-offs that are not well understood.

Here, we employed mouse infection models, within-host experimental evolution, and competitive infection experiments between isogenic strain pairs to explore how the microbiota affects virulence evolution of wild-type *S*. Typhimurium. Our data reveal that wild-type *S*. Typhimurium infection can disrupt the gut microbiota to such an extent that the gut luminal pathogen population evolves towards reduced virulence. The rise and long-term shedding of such mutants can be controlled by microbiota transfer. This identifies a previously unrecognized role of the microbiota in the selection for wild-type *S*. Typhimurium virulence within an infected host.

## Results

### In streptomycin pretreated mice, wild-type *S*. Typhimurium infection results in long-term fecal shedding of virulence-attenuated mutants

**Fig 1:**
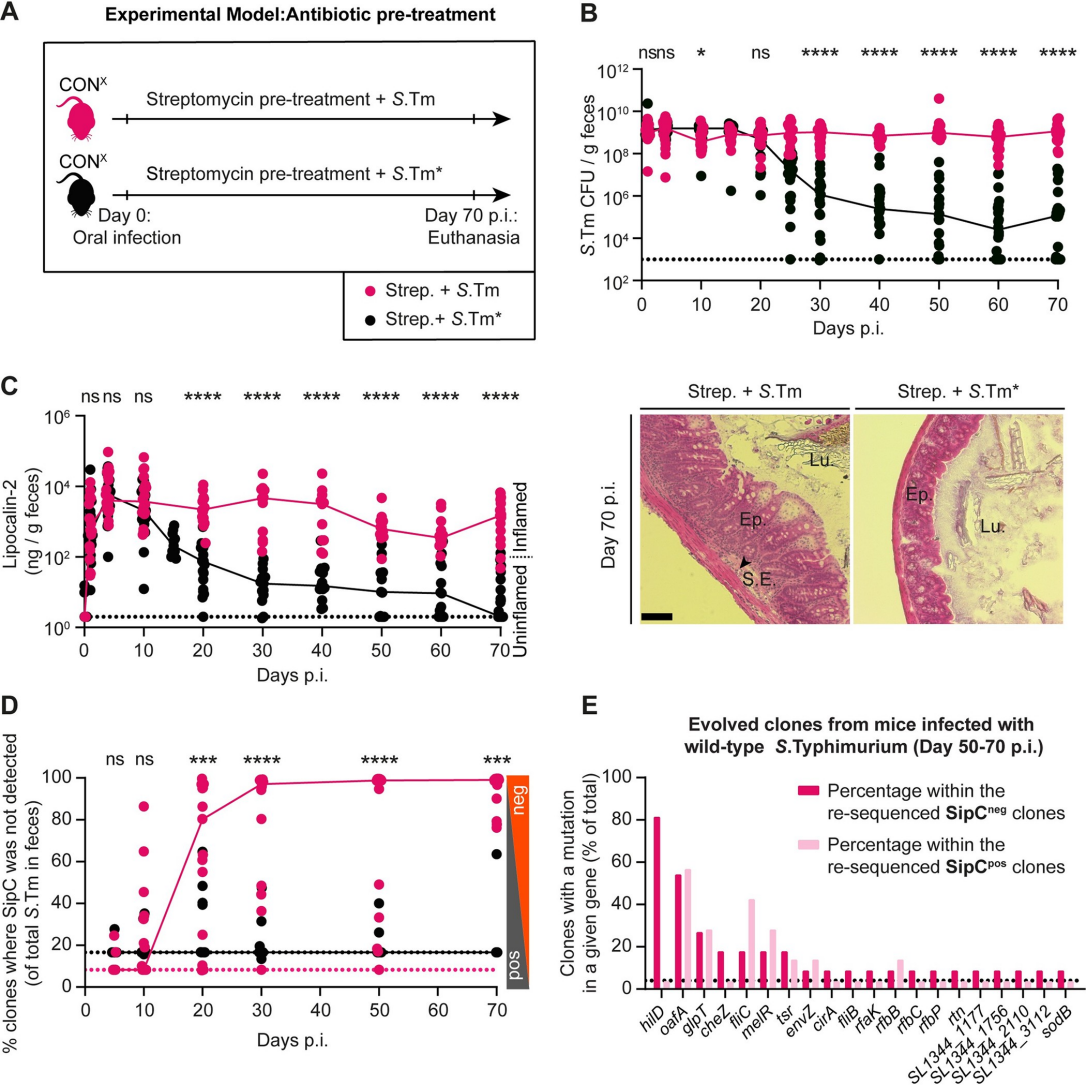
Selection for mutants with reduced virulence in streptomycin pretreated CON^X^ mice infected with wild-type *S*. Typhimurium or S.Tm*. (A) Scheme summarizing the experiment in Panels B–D. (B–D) Streptomycin pretreated CON^X^ mice were infected with wild-type *S*. Typhimurium (SL1344 WT; pink, filled circles; *n* = 19; 4 independent experiments) or *S*.Tm* (SL1344 *ΔssaV*; black, filled circles; *n* = 22; 3 independent experiments) for 70 days. (B) Fecal *Salmonella* populations as determined using MacConkey plates with selective antibiotics. (C) Gut inflammation. Left: ELISA data measuring the Lipocalin-2 concentration in fecal pellets. We analyzed samples from at least *n* = 11 animals per time point. Dotted lines indicate the detection limit. Colored lines connect the medians. Right: representative images of HE-stained cecum tissue sections showing intestinal crypts to assess the severity of enteric disease [19]; scale bar 100 μm. (D) Percentage of colonies without detectable SipC (as measured by colony protein blot). Colored lines connect the medians. Two-tailed Mann–Whitney U tests were used to compare the wild-type *S*. Typhimurium to the *S*.Tm* data (*p* ≥ 0.05 not significant (ns), *p* < 0.05 (*), *p* < 0.01 (**), *p* < 0.001 (***), *p* < 0.0001 (****)). Source data can be found in S1 Data file. (E) Whole-genome sequencing was performed from clones re-isolated from mice in Panels B–D. A complete overview of non-synonymous mutations is summarized in S1–S6 Tables. The graph illustrates the 20 genes most frequently mutated in clones re-isolated from mice infected with wild-type *S*. Typhimurium at day 50–70 p.i. Dark pink: mutations from clones without detectable SipC expression (*n* = 11 independent clones were analyzed). Light pink: mutations from clones where SipC was detected (*n* = 11 independent clones were analyzed). The dotted line indicates the percentage that corresponds to a mutation which occurs in just 1 clone. Genes mutated only in *mutS* mutant strains (i.e., mutator clones) are excluded. Only non-synonymous mutations and genes disrupted by stop codons or frameshifts are shown.

To assess how wild-type *S*. Typhimurium virulence evolves in the murine gut, we infected streptomycin pretreated mice with wild-type *S*. Typhimurium SL1344 (denoted as *S*. Typhimurium or *S*.Tm). *S*.Tm gut colonization has 2 stages in natural settings: (i) initial gut colonization which is promoted by any type of microbiota perturbation; and (ii) blooming and long-term shedding in the inflamed gut. In the streptomycin pretreated mouse model, the single dose of antibiotic which is applied 24 h before the infection transiently alleviates the CR and allows researchers to focus on the second stage, blooming in the inflamed gut [19,42,43]. Therefore, for our initial experiments, we selected this model to focus on the virulence-associated stages of the gut infection. As control, we infected a second group of mice with the *ssaV* deletion mutant of *S*. Typhimurium SL1344 (denoted as *S*.Tm*), an attenuated strain that lacks a functional TTSS-2 apparatus and which we had used in our proof-of-principle work on within-host evolution [29]. We chose 129SvEv mice for our initial experiments, as streptomycin pretreated 129SvEv mice develop overt enteropathy upon infection with wild type *S*. Typhimurium, which is similar to that of C57BL/6 mice that have been used most frequently in previous work [19,44] (S2A–S2F Fig). Like C57BL/6 mice, 129SvEv mice also permit gut-luminal *Salmonella* blooms. However, unlike C57BL/6 mice, 129SvEv mice express a functional *Nramp1* gene that is critical to restrict systemic spread of wild-type *S*. Typhimurium and have therefore been used not only in studies of chronic gut infections, but also in seminal work on transmission and persistent systemic infections [12,24,45]. This resembles the capacity of immunocompetent humans to control systemic spread of wild-type *S*. Typhimurium. These features of 129SvEv mice enable long-term studies of within-host evolution of the wild-type pathogen. Our 129SvEv mouse colony is bred under hygienic isolation and harbors a complex, specified pathogen-free microbiota which confers a high level of CR, as long as the microbiota remains unperturbed [25]. 129SvEv mice with this microbiota are denoted as CON^X^. Streptomycin pretreatment was used to transiently alleviate CR in the CON^X^ mice, and 24 hours later, the mice were infected with *Salmonella* via the natural orogastric route and remained in individually ventilated cages that form a strict hygienic barrier preventing the unintended introduction of new microbial strains (Fig 1A; Materials and methods).

In line with previous work [12,29], wild-type *S*. Typhimurium and *S*.Tm* colonized the gut lumen at high densities at days 1 to 10 of infection (≈10^9^ CFU/g stool; Fig 1B). Also, both strains elicited gut inflammation within the first 3 days of infection, as assessed by ELISA for the gut inflammation marker lipocalin-2 (Fig 1C, left side), which is expressed by the infected mucosa [29,46] and provides a reasonable assay for probing the time course of gut inflammation. In the *S*.Tm* infected mice, gut inflammation was resolved between days 20 to 70, as indicated by reduced lipocalin-2 concentrations in the stools, histopathological examination (Fig 1C, right side), cytokine mRNA expression analysis and neutrophil staining in gut tissues at day 70 (S3A–S3C Fig). Further, the gut-luminal pathogen population declined below 10^5^ CFU per gram (CFU/g) feces in mice infected with *S*.Tm* between days 25 to 70 p.i. (Fig 1B). This was strikingly different compared to the wild-type *S*. Typhimurium infected mice, where gut inflammation and fecal pathogen loads remained much higher than in the *S*.Tm* infected group until day 70 p.i. (Figs 1B, 1C, and S3A–S3C).

Next, we asked if mutant clones with attenuated virulence arise in the gut lumen. Earlier work using genetically barcoded *S*. Typhimurium populations had shown that the pathogen population structure in the cecum lumen is established by bacterial growth, influx, efflux, and death and that the *Salmonella* population structures of the feces resemble those in the cecum [46–49]. Thus, by analyzing the feces, we could assess the clonal composition of the *Salmonella* population within the host (that is the cecum lumen) and obtain information about the pathogen population that can be transmitted to new hosts. The fecal *S*. Typhimurium population was analyzed using a colony protein blot assay [29,50] (Materials and methods). We plated dilutions of fecal material on MacConkey agar, replica-plated the colonies onto nitrocellulose and lysed the *Salmonella* cells to transfer the proteins expressed by each colony onto the membrane. As wild-type *S*. Typhimurium expresses detectable amounts of TTSS-1 on such plates, we could use an antibody for the TTSS-1 translocon protein SipC to detect colonies capable of TTSS-1 expression (S1B Fig; [29,50]). This assay revealed a further striking difference between the pathogen populations in the 2 groups of mice. While *S*.Tm*-infected mice shed pathogen populations dominated by clones expressing TTSS-1 (SipC detected in >95% of all colonies; Figs 1D and S4), such clones were much less frequent in the second group of mice. In CON^X^ mice pretreated with streptomycin and infected with wild-type *S*. Typhimurium, we detected SipC in <2% (median of all mice) of the fecal *Salmonella* colonies by day 30 p.i. and this fraction remained extremely low until day 70 p.i. (Figs 1D and S4). Overall, the wild-type *S*. Typhimurium infection yields lower numbers of fecal *Salmonella* cells capable of expressing SipC, than the mutant *S*.Tm* infection mice (S4C Fig). At the same time, the absolute number of fecal *Salmonella* cells without detectable SipC expression was 10^4^-fold higher. This suggested that wild-type *S*. Typhimurium infection in streptomycin CON^X^ mice selects for pathogen mutants with reduced virulence and that this selection was much more pronounced than with *S*.Tm*.

To understand the genetic nature of the evolved clones without detectable SipC expression, we isolated and genome-sequenced several colonies from the feces at different times after wild-type *S*. Typhimurium infection or from *S*.Tm* infections based on their Colony Blot phenotypes (i.e., SipC detected, undetected, or intermediate). With this approach, we isolated 15 clones from wild-type *S*. Typhimurium infections and 12 from *S*.Tm* infections, which do not express SipC. Furthermore, colonies expressing SipC (intermediate or high; 22 from wild-type *S*. Typhimurium infections; 21 from *S*.Tm* infections) were genome-sequenced as controls (S1–S6 Tables). We reasoned that these numbers of clones should allow us to survey mutations that are positively selected and to distinguish them from mutations hitchhiking on the mutations selected for. At day 70 p.i., the majority of clones without detectable SipC expression featured mutations in the HilD regulon, mostly disrupting its master regulator gene *hilD* (Fig 1E and S1–S6 Tables). In wild-type *S*. Typhimurium-infected mice, 82% of all clones without detectable SipC had such *hilD* mutations at day 50 to 70 p.i. This is in line with the critical role of *hilD* in the expression of the TTSS-1 translocator protein SipC and suggests that *hilD* mutants are selected for in the wild-type *S*. Typhimurium-infected mice. In addition, we observed multiple clones with mutations in other genes implicated in *Salmonella* colonization and virulence, such as LPS biosynthesis, metabolism, chemotaxis, or flagella biosynthesis, all of which are indicative of a trend of within-host evolution towards reduced virulence (Fig 1E; Supplementary discussion A in S1 Text). We decided to focus on the *hilD* gene in our further experiments, as *hilD* was previously shown to be the most frequently mutated gene in animal models and natural isolates, and earlier work has established the central role of HilD in *Salmonella* virulence in antibiotic pretreated mice [29,41,51,52].

### The evolved *S*. Typhimurium population, dominated by *hilD* mutants, shows reduced virulence

**Fig 2:**
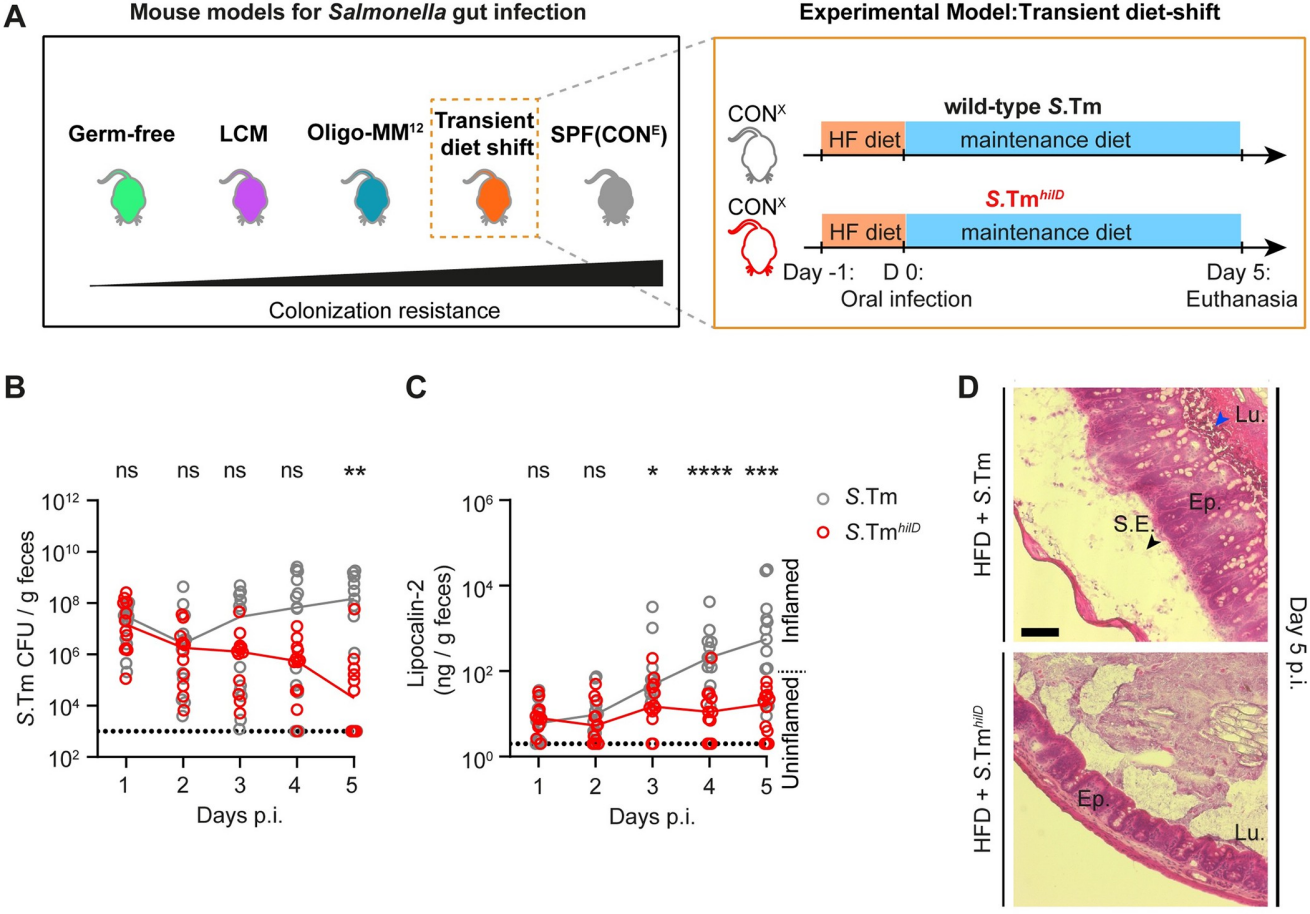
Reduced virulence of *S*.Tm^*hilD*^ in the high-fat diet shift model. (A) Experimental scheme. CON^X^ mice that were shifted to high-fat diet for 1 day (and shifted back to maintenance diet on the day of infection) were infected for 5 days with wild-type *S*. Typhimurium (gray, empty circles; *n* = 13 mice) or *S*.Tm^*hilD*^ (red, empty circles; *n* = 12 mice; 5 × 10^7^ CFU, by gavage). (B) *Salmonella* loads in the feces, as determined using MacConkey plates with selective antibiotics. (C) Lipocalin-2 centration in the feces, as determined by ELISA. Dotted lines indicate the detection limit. Colored lines connect the medians. (D) Representative images of HE-stained cecum tissue sections; scale bar 100 μm. The data were obtained in 2 independent experiments. Two-tailed Mann–Whitney U tests were used to compare the wild-type *S*. Typhimurium to the *S*.Tm^*hilD*^ data (*p* ≥ 0.05 not significant (ns), *p* < 0.05 (*), *p* < 0.01 (**), *p* < 0.001 (***), *p* < 0.0001 (****)). Source data can be found in S1 Data file.

We performed 3 types of infection experiments to test the reduced virulence of the *hilD* mutant-dominated *Salmonella* population that evolved in wild-type *S*. Typhimurium infected mice by day 70 p.i. (from Fig 1A–1D). In the first experiment, the feces were suspended in PBS and then used to infect naïve streptomycin pretreated CON^X^ mice via the orogastric route for 3 days (S5A Fig). Compared to control infections with the original wild-type *S*. Typhimurium strain, the evolved population reached equivalent gut-luminal densities (≈10^9^ *Salmonella* cells per gram feces; S5B Fig), but caused significantly less gut inflammation, as judged by a reduced lipocalin-2 concentration in the feces (S5C Fig). In a second experiment, we sought to measure the virulence attenuation of *hilD* mutants in a system that retains a more intact gut microbiota than the streptomycin pretreated mice to better resemble natural infections. Therefore, we turned to the “high-fat diet shift” protocol [25] (Fig 2A). Earlier work had established that shifting CON^X^ mice for 24 h from the normal plant-based mouse chow to a lard-based high-fat diet will transiently disturb the microbiota to temporarily alleviate CR (though less efficiently than in streptomycin pretreated mice). This permits wild-type *S*. Typhimurium growth in the intestinal lumen, resulting in shedding of high numbers of *Salmonella* cells in the feces. In natural settings, such increased fecal shedding would be expected to promote fecal-oral transmission. After the high-fat diet shift, the orogastric infection with wild-type *S*. Typhimurium elicits enteropathy within 3 to 4 days. These disease kinetics are significantly delayed compared to the streptomycin pretreated mouse model, where enteropathy is observed as early as 8 to 12 h after infection. We assume that this is due to the milder microbiota perturbation, which leads to slower gut colonization in the high-fat diet shift model. In the high-fat diet shift model, therefore, inflammation-dependent bloom of the luminal pathogen population is later and less pronounced than in streptomycin pretreated mice. After shifting CON^X^ mice for 24 h to high-fat diet (Materials and methods), we infected them with either wild-type *S*. Typhimurium or an isogenic *hilD* mutant, in which the gene had been recombinantly deleted (termed *S*.Tm^*hilD*^), by gavage. Wild-type *S*. Typhimurium maintained higher fecal loads and elicited more pronounced enteropathy, while *S*.Tm^*hilD*^ loads declined during the 5 days of infection and caused attenuated enteropathy with delayed kinetics as judged by lipocalin-2 ELISA and by histopathology (Fig 2B–2D). Equivalent observations were made in the experiment shown in S6 Fig, in which we infected gnotobiotic mice that harboring 2 different microbiotas that confer intermediate degrees of CR and can thus be infected without prior microbiota perturbation. In these models, as seen previously, wild-type *S*. Typhimurium maintained higher fecal loads and elicited more pronounced enteropathy than *S*.Tm^*hilD*^. Based on this evidence, we conclude that *hilD* mutants have a reduced virulence compared to wild-type *S*. Typhimurium and can be displaced by the gut microbiota, such as the one that remains in the diet-shift model.

Our data so far established that wild-type *S*. Typhimurium evolves during the infection of streptomycin pretreated mice yielding pathogen populations that are dominated by *hilD* (and other) mutants, and that such attenuated mutants are shed in the feces at high densities for months. These evolved mutants did not show any sign of reversal upon infection of the next host, but instead showed reduced ability to trigger enteric disease. Thus, wild-type *S*. Typhimurium evolved towards reduced virulence. This was strikingly different from earlier work re-capitulated in the experiments with *S*.Tm*, which served as control in the current study. In these experiments, we observed only transient selection for *hilD* mutants, and the gut-luminal *Salmonella* population was displaced by days 20 to 60 p.i., without accumulation of evolved mutants. Overall, attenuated virulence was selected for more strongly and over much longer periods of time in wild-type *S*. Typhimurium than in *S*.Tm* infections. However, it had remained unclear which feature of the tripartite interaction between the wild-type pathogen, the microbiota and the host’s immune response would promote the selection for reduced virulence in the wild-type *S*. Typhimurium infection.

### Microbiota transfer displaces the gut luminal *Salmonella* population dominated by *hilD* mutants

First, we probed the role of the microbiota in displacing *Salmonella* populations that had evolved towards reduced virulence. Specifically, we hypothesized that the wild-type *S*. Typhimurium infection may disrupt the gut microbiota to such an extent that it loses its ability to displace the *hilD* mutant-dominated pathogen population selected for during the infection. Such pronounced microbiota disruption seemed plausible, as inflammation is known to alter the gut microenvironment and suppress regrowth of the microbiota, promoting gut luminal growth of *Salmonella* spp. and related enteric bacteria [12,31,32,53]. Moreover, in streptomycin pretreated mice, gut inflammation induced by wild-type *S*. Typhimurium elicits such a pronounced anti-microbial defense that even gut-luminal loads of the inflammation-adapted pathogen transiently decline by 10 to 10,000-fold at day 2 of the infection [48,49] before regrowing to carrying capacity (≈10^9^ CFU/g in cecum content or feces). Based on this previous knowledge and our data presented above, we hypothesized that wild-type *S*. Typhimurium might disrupt the gut microbiota “beyond return”, so that it cannot regrow when the virulence of the pathogen population declines and therefore fails to displace the mutant-dominated gut luminal population at the end of an infection. It is important to note that the experiments above (as well as our previous work [12,28,29,52]) were performed using individually ventilated cages. This hygienic isolation prevents access to microbiota from other sources. Therefore, once a particular member of the microbiota is lost, it cannot be naturally re-acquired [54]. We reasoned that the long-term prevalence of HilD-regulon mutants in mice infected wild-type *S*. Typhimurium might be promoted by hygienic isolation.

**Fig 3:**
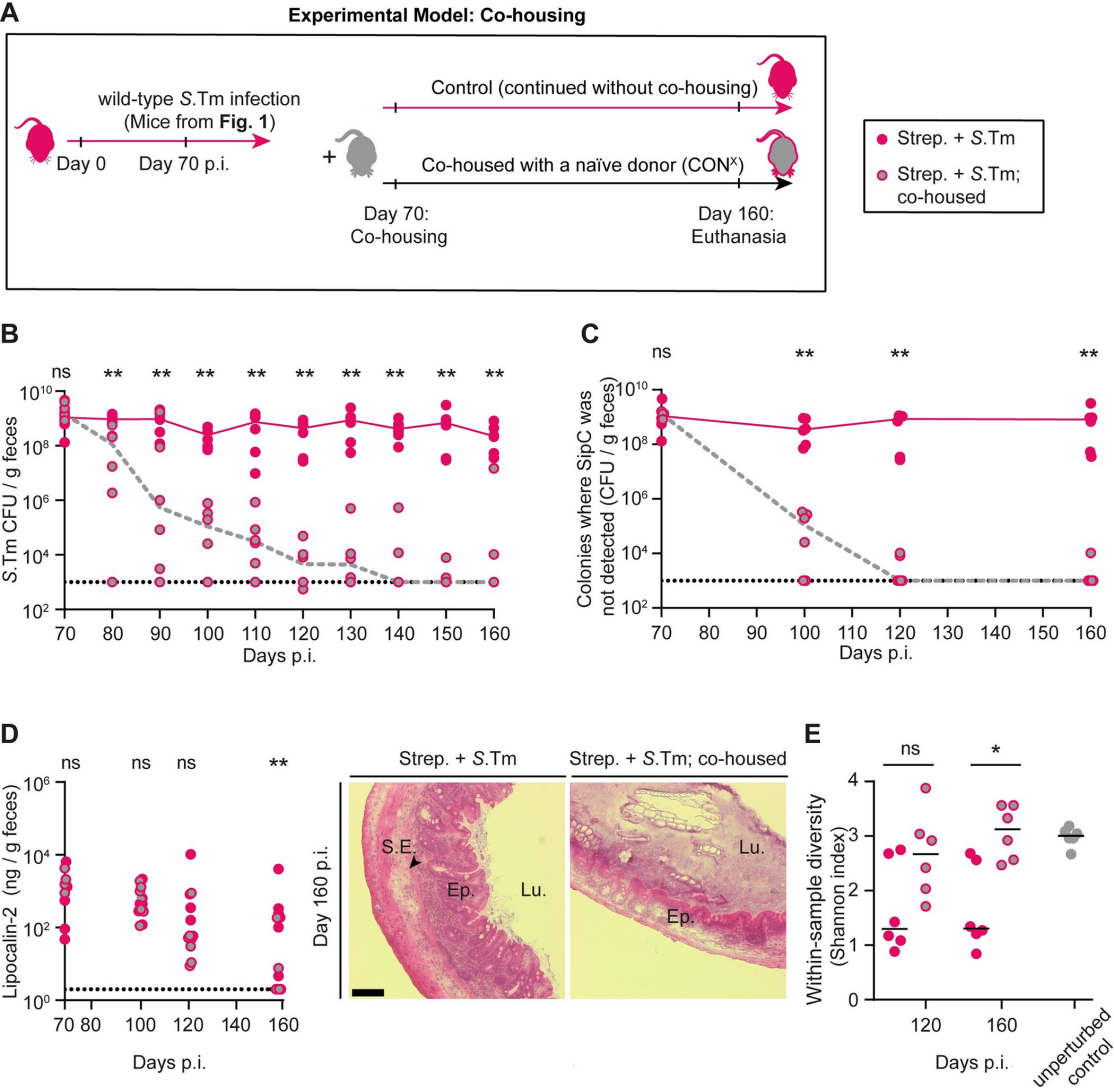
Microbiota transfer from uninfected CON^X^ mice displaces mutant-dominated *Salmonella* populations from wild-type *S*. **Typhimurium infected mice.** (A) Experimental scheme. After day 70 of infection with wild-type *S*. Typhimurium (from Fig 1A–1E; n = 12) the mice were either co-housed with an untreated CON^X^ mouse (pink, gray filled circles; n = 6 mice; each infected mouse was caged with a healthy mouse) or kept under hygienic isolation (pink filled circles; *n* = 6 mice). (B) *Salmonella* population sizes as determined using MacConkey plates with selective antibiotics. (C) Size of *Salmonella* population that did not yield SipC signals in the colony protein blot assay, as determined by multiplying the percentage of the colonies without detectable SipC signal with the pathogen population (as shown in B). (D) Gut inflammation. Left: Lipocalin-2 concentration in the feces, as determined by ELISA (fecal pellets from at least *n* = 6 mice analyzed per time point). Dotted lines indicate the detection limit. Lines connect the median values at the days of analysis. Right: representative images of HE-stained cecum tissue sections; scale bar 100 μm. (E) Microbial community analysis of fecal samples at day 120 and day 160 p.i. The within-sample diversity was measured using the Shannon Index (pink circles: no co-housing, pink, gray filled circles: co-housed; gray circles; feces from donor mice (that is unperturbed CON^X^ animals)). The data shown was obtained from 2 independent experiments including comparing both groups. Two-tailed Mann–Whitney U tests were used to compare the data from mice with or without co-housing at each time point (*p* ≥ 0.05 not significant (ns), *p* < 0.05 (*), *p* < 0.01 (**), *p* < 0.001 (***), *p* < 0.0001 (****)). Source data can be found in S1 Data file.

Microbiota transfer experiments are a gold standard for demonstrating microbiome functions in mouse physiology and disease research [55]. To probe into the role of the microbiota in displacing *hilD* mutant-dominated *Salmonella* populations, we performed 2 types of microbiota transfer experiments. Both approaches relied on co-housing, as previous work had established that this mediates microbiota transfer into *Salmonella*-infected mice [56]. First, we asked if microbiota transfer from naive hosts with a complex microbiota (that is uninfected CON^X^ mice) could terminate the continuous gut colonization by the *hilD* mutant-dominated *Salmonella* population that rises in the wild-type *S*. Typhimurium infection. We co-housed mice at the end of the experiment shown in Fig 1A–1D (that is at day 70 p.i. with wild-type *S*. Typhimurium; SipC detected in <2% of all colonies), with untreated CON^X^ animals (Fig 3A). Compared to control mice without co-housing, the total *Salmonella* loads in the feces and the fraction of *Salmonella* clones without detectable SipC were significantly reduced and dropped below the detection limit in most mice by day 120 (<10^3^ CFU/g feces; Fig 3B and 3C). Moreover, in addition to decreasing detectable lipocalin-2, cytokine mRNA expression, neutrophil staining and histopathology indicated that co-housing enhanced the resolution of gut inflammation (Figs 3D and S7).

In control mice without co-housing, the fraction of *Salmonella* clones without detectable SipC remained high (Fig 3B and 3C) and the pathogen population accumulated further mutations (day 160 p.i.; median = 8 mutations per clone; S8 Fig and S3 Table). Thus, under hygienic isolation, HilD-regulon mutants prevailed.

To confirm that co-housing achieved microbiota transfer, we compared the gut microbiome composition between both groups of mice using 16S rRNA sequencing of fecal samples taken from mice in Fig 3B–3D (day 120 and 160 p.i.). The co-housed mice re-gained a significantly higher community diversity compared to the control animals (Fig 3E; within-sample diversity measured using Shannon index). In fact, co-housing re-established a similar within-sample diversity as observed in the naïve CON^X^ “donor” mice (Fig 3E, compare gray circles with and without red lining). In line with this, co-housing restored microbiota composition at least in part, as indicated by the principal coordinate analysis (PCoA) based on Bray–Curtis dissimilarities at day 160 p.i. and by the analysis of microbiota composition (S9 Fig).

Altogether, this first transfer experiment suggested that transferring a complex microbiota can resolve chronic gut inflammation and displace the gut luminal HilD-regulon mutant-dominated pathogen population, which evolved during wild-type *S*. Typhimurium infection.

### Microbiota transfer shows that the microbiota of *S*.Tm*-infected mice retains its capacity to displace a *Salmonella* population dominated by *hilD* mutants

**Fig 4:**
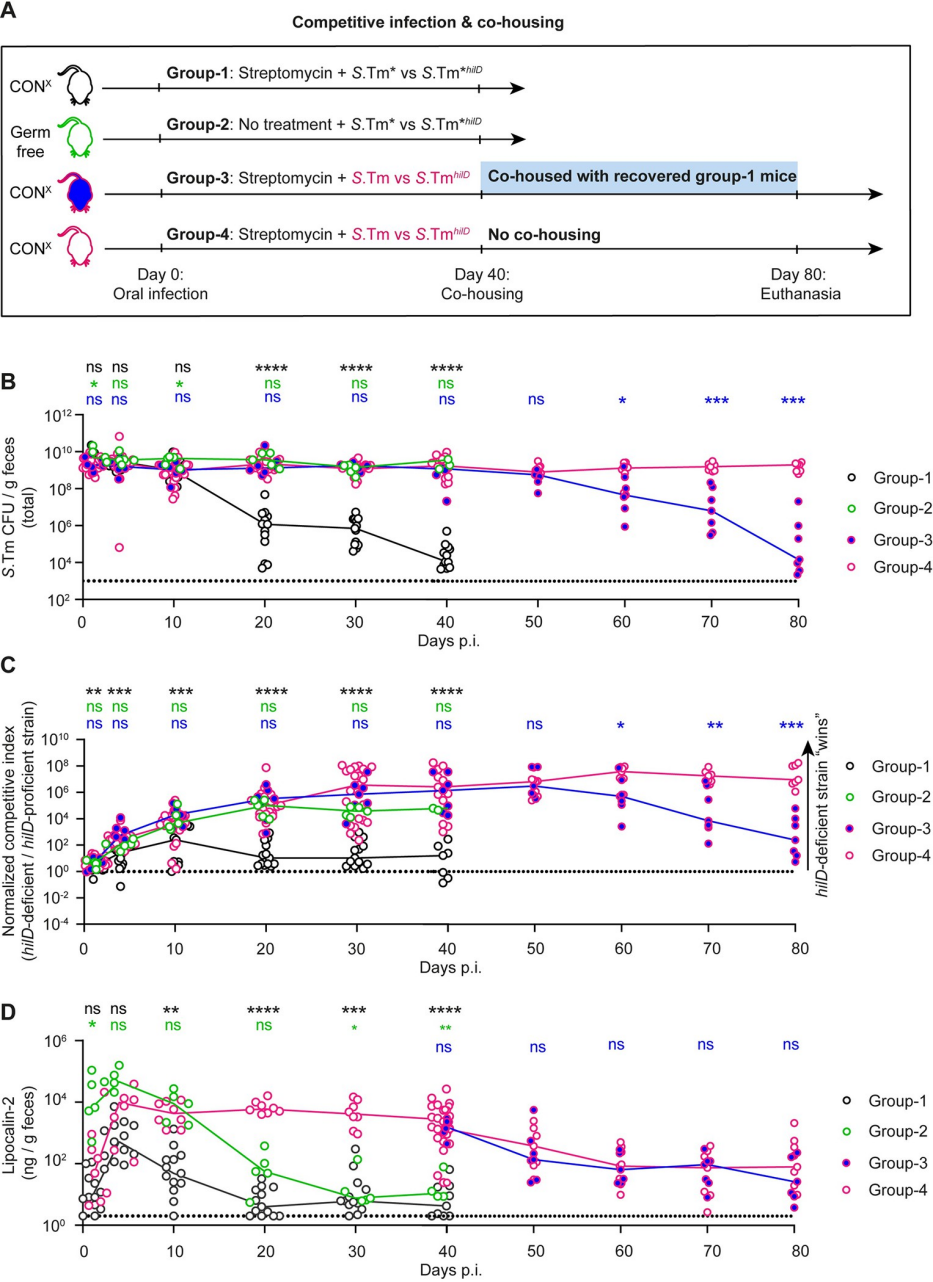
Microbiota transfer from *S*.Tm*-infected mice can displace the mutant-dominated pathogen population selected for during wild-type *S*. **Typhimurium infection.** (A) Experimental scheme. We employed 4 groups of mice: **1.** Streptomycin pretreated CON^X^ mice were infected for 40 days with a 1:10 (*n* = 6 mice) or a 100:1 (*n* = 7 mice) *S*.Tm* vs. *S*.Tm*^hilD^ (black symbols with white filling; *n* = 13 mice; 5 × 10^7^ CFU, by gavage). By day 40 p.i., this group yielded the donor mice (black). **2.** Germ-free C57BL/6 mice were infected for 40 days with a 100:1 mix of *S*.Tm* vs. *S*.Tm*^hilD^ (green symbols with white filling; *n* = 5 mice; 5 × 10^7^ CFU, by gavage). **3. and 4.** Streptomycin pretreated CON^X^ mice were infected for 40 days with a 1:10 (*n* = 8 mice) or a 100:1 (*n* = 20 mice) mix of *S*.Tm* vs. *S*.Tm*^hilD^ (pink symbols with blue filling, or pink symbols with white filling respectively; 5 × 10^7^ CFU, by gavage). By day 40 p.i., this yielded the 2 experimental groups. **3.** At day 40, we co-housed mice from group 3 (*n* = 7 mice) with mice from group 1 and followed the infection until day 80. **4.** The mice remained under hygienic isolation (*n* = 7 mice) until day 80 p.i. (B) Total *Salmonella* loads in the feces, as determined using MacConkey plates with selective antibiotics. (C) Competitive index of the *hilD*-deficient vs. the *hilD*-proficient strains in the respective groups, as calculated from selective plating data. (D) Lipocalin-2 concentration in the feces, as determined by ELISA (fecal pellets from 5 to 14 mice per group, as indicated per group and per time point). Colored lines connect the medians. For each group, we show the pooled data from at least 2 independent experiments. Group 2 shows data from 1 experiment with 2 cohorts. Two-tailed Mann–Whitney U tests were used to compare the indicated groups: black symbols: group 1 vs. groups 4 (between days 0–40 p.i.); green symbols: group 2 vs. group 4 (between days 0–40 p.i.); blue symbols: group 3 vs. group 4 (between days 0–80 p.i.). (*p* ≥ 0.05 not significant (ns), *p* < 0.05 (*), *p* < 0.01 (**), *p* < 0.001 (***), *p* < 0.0001 (****)). Source data can be found in S1 Data file.

In an alternative type of microbiota transfer experiment, we probed into the capacity of the gut microbiota remaining after 1 round of *S*.Tm* infection to displace wild-type *S*. Typhimurium *hilD* mutants. As microbiota regrowth is thought to displace the gut luminal *Salmonella* population at the end of such *S*.Tm* infections (as in the controls in Fig 1A–1D), we reasoned that the “recovered” microbiota might also be capable of displacing the *hilD* mutant-dominated pathogen population that is selected for in wild-type *S*. Typhimurium infected hosts. Here, we changed our experimental set-up slightly to more precisely quantify the selection for or against *hilD* mutants. To this end, we infected streptomycin pretreated CON^X^ mice with a recombinant *hilD* deletion mutant and its isogenic *hilD*-proficient counterpart carrying antibiotic selection markers. This allowed us to precisely monitor the size of each population in feces. Furthermore, we infected these mice for 40 days instead of 70 days, as this was sufficient for *hilD* mutant cells to dominate the population (Fig 1A–1E). We used an inoculum in which *hilD*-proficient cells were 10- or 100-fold more abundant than the isogenic *hilD*-deficient mutant (*S*.Tm*^hilD^) (Fig 4A). The higher surplus of *S*.Tm* was chosen to mimic the selective environment from the experiments in Fig 1A–1D. Specifically, this generated an *S*.Tm*-conditioned inflammatory milieu in the gut lumen during the first days of the infection that allowed for the selection for initially rare *hilD* mutants. This competitive infection allowed us to verify that the regrowing microbiota had successfully displaced the gut luminal *Salmonella* population and prevented further selection for *hilD* mutants.

In the first stage of this experiment, we generated 4 groups of mice. The first group provided the “microbiota donor” mice. The second group was designed to probe into the importance of the microbiota in displacing the gut-luminal *Salmonella* population in the first group. To this end, we infected germ-free C57BL/6 mice with the same inoculum that we had used for the first group (that is *S*.Tm* versus *S*.Tm*^hilD^; 100:1). In contrast to the first group, the second group (that is, the germ-free mice) shed highly dense *Salmonella* populations dominated by *S*.Tm*^hilD^ until day 40 p.i. (≈10^9^ CFU/g feces; Fig 4B and 4C). Strikingly, the second group retained this high *S*.Tm*^hilD^ colonization in spite of falling lipocalin-2 levels, which are indicating the resolution of gut inflammation by days 30 to 40 p.i. (Fig 4D). These observations supported the hypothesis that the microbiota is essential for displacing the gut luminal pathogen population that evolves during the course of the *S*.Tm* infection (as observed in the first group and in our previous work) [29]. Also, these data confirmed that the selection for *hilD* mutants occurs in different mouse lines (C57BL/6 and 129SvEv) [29] and that enteropathy can resolve (at least partially) in the absence of microbiota, as indicated by the declining lipocalin-2 levels (from >10^5^ ng/g feces at day 3 p.i. down to 10^1^ ng/g feces at day 30 to 40 p.i.).

The third and the fourth groups were the experimental groups of our transfer experiment. In groups 3 and 4, we infected streptomycin pretreated CON^X^ mice with a 10:1 or a 100:1 mixture of wild-type *S*. Typhimurium versus *S*.Tm^*hilD*^ (Fig 4A; *S*.Tm: *S*.Tm ^*hilD*^). This experimental setup allowed us to accurately quantify the selection for the *hilD* mutant by differential plating using unique antibiotic resistances for wild-type *S*. Typhimurium and *S*.Tm^*hilD*^ (Table 1). Furthermore, the setup established an inflammatory gut-luminal milieu conditioned by wild-type *S*. Typhimurium and created a situation in which *hilD* mutants were initially rare and subsequently selected for to dominate the gut lumen (days 1–40 p.i.; Fig 4B–4D). Of note, infections with both 1:10 and 1:100 inoculate mixtures yielded equivalent outcomes and therefore are equally represented in the co-housing experiments after day 40 p.i. The data for the first 40 days were consistent with the results from the wild-type *S*. Typhimurium infection shown in Fig 1A–1D (that is we observed the selection for *S*.Tm^*hilD*^ over wild-type *S*. Typhimurium; reaching ≈10^9^ CFU/g feces). At day 40, we placed mice from the first group as “microbiota donors” into the cages of the third group. This reduced the total *Salmonella* loads in the feces and the selection for *S*.Tm^*hilD*^ compared to the fourth group which remained without donor mice (Fig 4B and 4C). The displacement of the *hilD* mutant-dominated gut luminal *Salmonella* population went along with a slight amelioration of the gut inflammation, as indicated by histopathological inspection (S10 Fig). However, the resolution of enteropathy was incomplete and fecal lipocalin-2 concentrations remained much higher than in non-infected mice and did not differ between groups 3 and 4 (Fig 4D). This data demonstrated that the gut microbiota re-establishing during recovery from *S*.Tm*-induced colitis retains its capacity to displace the gut-luminal *hilD* mutant-dominated pathogen population selected for in wild-type *S*. Typhimurium infected mice. This displacement can occur in the face of gut inflammation, as observed in wild-type *S*. Typhimurium infected mice by days 40 to 80 p.i.

Together, our 2 microbiota transfer experiments established that *hilD* mutants are displaced by the microbiota. The lack of microbiota (as in germ-free mice) or its irreversible disruption (as in streptomycin-treated CON^X^ mice infected with wild-type *S*. Typhimurium) promotes within-host evolution of the pathogen towards reduced virulence. Therefore, the microbiota appears to be critical for creating a selective environment that promotes the maintenance of *Salmonella* virulence.

### Gut inflammation selects for *hilD* mutants independent of the *S*.Tm strain background

To this point, our experiments established that wild-type *S*. Typhimurium infections differ from *S*.Tm* infections in their tendency to select for *hilD* mutants and the massive fecal shedding of these virulence-attenuated mutants for weeks and months. Furthermore, our data suggested that this might be attributable to the different capacities of these 2 strains to trigger enteropathy and thereby disturb the gut microbiota. However, it remained possible that pleiotropic effects pertaining to the strain backgrounds, e.g., the *ssaV* deletion mutation, which attenuates tissue colonization by *S*.Tm* relative to wild-type *S*. Typhimurium, might contribute to the selection for *hilD* mutants in the gut lumen. We addressed this using 2 different approaches.

In the approach shown in S11 Fig, we used a mixture of isogenic *S*.Tm strains as an inoculum where a strain given in 10,000-fold excess conditions the gut milieu to either remain “uninflamed” (*S*.Tm lacking *invG ssaV; S*.Tm^Avir^; cannot trigger substantial gut inflammation) or to feature wild-type enteropathy (wild-type *S*.Tm). Within the 10,000-fold excess of the conditioning strain, we mixed a 1:1 ratio of focal competing strains that were either *hilD*-deficient or -proficient *S*.Tm mutants constructed in either the *S*.Tm^Avir^ or wild-type *S*.Tm background (scheme in S11A Fig). By this way, we could disentangle if the strain background or instead the inflamed gut milieu selects for *hilD* mutants. In these 4 conditions, we analyzed the competition between isogenic *S*. Typhimurium strain pairs. Strikingly, when the gut is conditioned to remain “uninflamed” (that is by using a 10,000-fold excess of *S*.Tm^Avir^), the *hilD*-proficient strain and its isogenic *hilD-*deficient mutant remained at a 1:1 ratio throughout the 4 days of the infection (competitive index, C.I. = 1; S11 Fig). This was independent of the strain background used. In contrast, in the positive control experiment where untagged wild-type *S*. Typhimurium is used in 10,000-fold excess and triggered pronounced enteropathy, the *hilD* mutant outcompeted the tagged *hilD*-proficient strain by 1,000-fold within 4 days. Indeed, the same held true when we competed *S*.Tm^Avir^ (*S*.Tm SL1344 *invG ssaV*) against its *hilD*-deficient derivative in the presence of 10,000-fold excess of wild-type *S*. Typhimurium (S11 Fig). Thus, gut inflammation selects for *hilD* mutants, regardless of the presence or absence of *ssaV* in the competing strain pairs or their capacity to invade and grow within host tissues.

The second approach we applied is depicted in S12 Fig. Here, we used an experimental setup in which the infections are performed with the same strain (i.e., wild type *S*. Typhimurium) but the kinetics of gut inflammation differ due to the use of different mouse models. Therefore, we could address if the selection for or against *hilD* mutants is affected by the degree of gut inflammation. To this end, we performed serial transmission experiments in 2 different types of isogenic C57BL/6 mice. The serial fecal-oral transmission allowed us to limit the infection in the individual mice to 3 or 4 days and thereby avoid death from systemic pathogen spread in C57BL/6 mice. We used 2 groups of C57BL/6 mice differing in both the degree of CR conferred by their microbiota and the disease kinetics. Gut inflammation occurs within 24 h in streptomycin pretreated C57BL/6 (CON^E^; Materials and methods) mice, while it is delayed to day 3 to 4 p.i. in untreated C57BL/6 mice that are associated with a defined microbiota composed of 12 representative microbiota strains (OligoMM^12^ mice [57]). Both types of mice were infected with the same wild-type *S*. Typhimurium strain. While OligoMM^12^ mice shed pathogen populations dominated by clones expressing TTSS-1 until the end of the experiment (SipC detected in 100% of all colonies; S12 Fig), this population declined much faster in the streptomycin pretreated CON^E^ mice. Thus, we conclude that the selection for *hilD* mutants is not related to the strain background, but that it is much rather determined by the gut-luminal milieu shaped by gut inflammation and initial microbiota composition.

Altogether, these data suggest that the gut milieu created as a result of wild-type *S*. Typhimurium-triggered inflammation selects for *hilD* mutants regardless of the strain background (e.g., wild-type, *ssaV* mutant, or avirulent). However, it remained unclear whether microbiota transfer can prevent selection for *hilD* mutants during the acute phase of the wild-type *S*. Typhimurium infection (e.g., in streptomycin pretreated mice).

### Microbiota transfer can prevent selection for attenuated *hilD* mutants during the acute phase of wild-type *S*. Typhimurium infection

**Fig 5:**
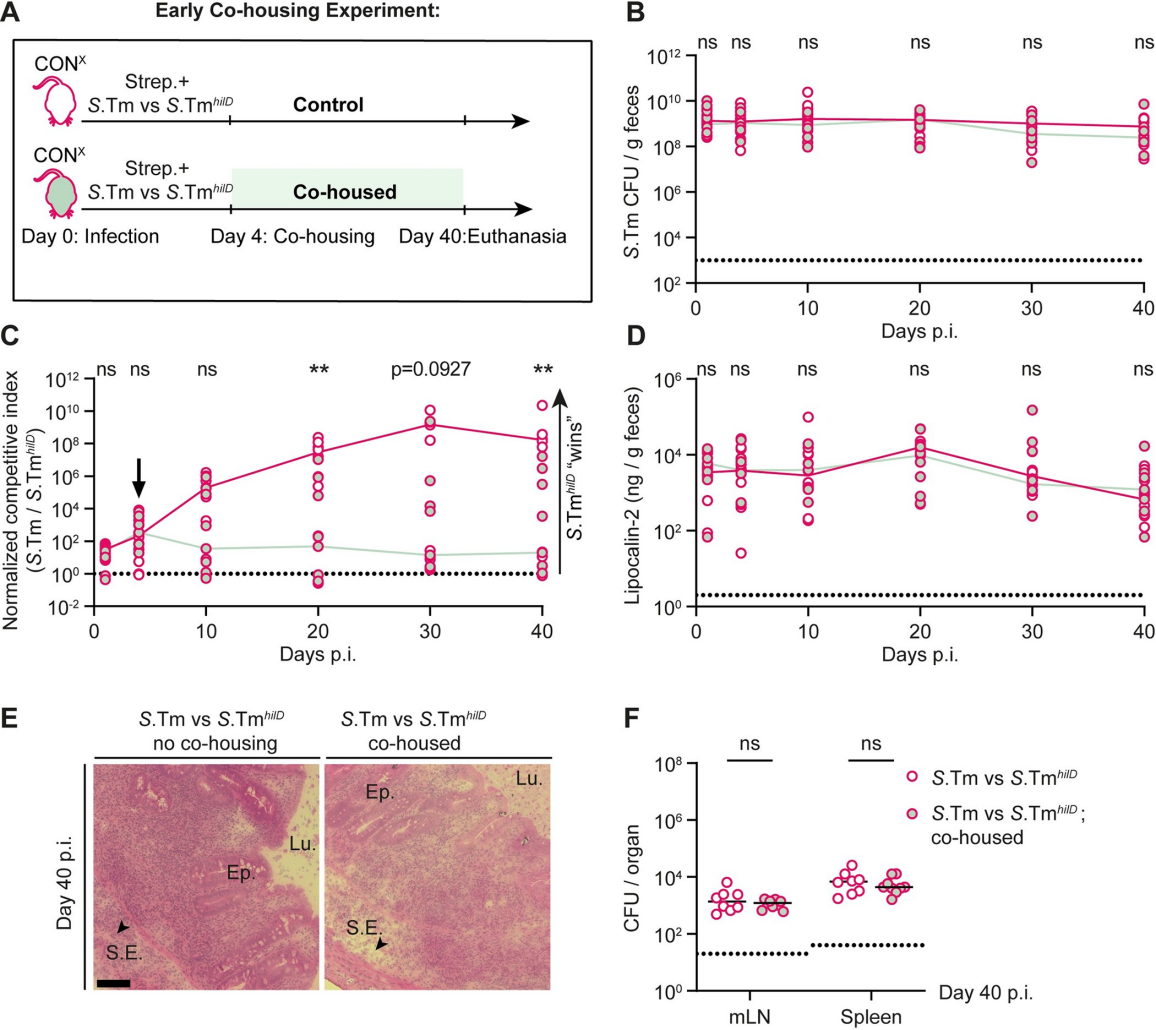
Early microbiota transfer experiment to test the effect of the microbiota transfer before *hilD* mutants dominate the fecal population. (A) Experimental scheme. (B–F) Streptomycin pretreated CON^X^ mice (*n* = 8 or *n* = 9 per group) were infected with a 10^6^:1 mixture of wild-type *S*. Typhimurium and an isogenic *hilD* mutant (5 × 10^7^ CFU, by gavage). The first group remained in hygienic isolation (control; pink empty circles; *n* = 8), while the second group was co-housed from day 4 on with a naïve CON^X^ mouse (pink, light green filled circles; *n* = 9). The groups were kept as such until the end of the experiment (day 40 p.i.). (B) Total *Salmonella* loads detected in the feces by selective plating. Dotted lines indicate the detection limit. Colored lines connect the medians. (C) The C.I. as determined using MacConkey plates with selective antibiotics and shown for wild-type *S*. Typhimurium versus *S*.Tm^*hilD*^. The dotted line indicates a C.I. of 1. (D) An ELISA for fecal lipocalin-2 was used to compare gut inflammation between the 2 groups. (E) Representative images of HE-stained cecum tissue sections; scale bar 100 μm. (F) Total *Salmonella* organ loads. Lines indicate the median. mLN = mesenteric lymph node. The data shown was obtained from 2 independent experiments including comparing both groups. Two-tailed Mann–Whitney U tests were used for statistical analysis (*p* ≥ 0.05 not significant (ns), *p* < 0.05 (*), *p* < 0.01 (**), *p* < 0.001 (***), *p* < 0.0001 (****)).Source data can be found in S1 Data file.

Our results so far show that (i) gut inflammation is necessary for efficient gut colonization; (ii) but also selects for attenuated *hilD* mutants; while (iii) microbiota transfer from a naïve or recovered donor mouse clears *S*.Tm population dominated by *hilD* mutants from the gut lumen, which can indirectly contribute to maintenance of the wild-type *S*. Typhimurium genotype. Next, we asked whether a complex microbiota is also capable of preventing the rise of *hilD* mutants in the first place during the conditions in which the gut milieu is shaped by wild-type *S*. Typhimurium-triggered inflammation. To address this, we performed microbiota transfer experiments during the acute phase of the infection in our streptomycin pretreated mouse model and monitored the kinetics of selection for *hilD* mutants (Fig 5A).

To this end, we infected 2 groups of streptomycin pretreated CON^X^ mice for 40 days with a 10^6^:1 mixture of wild-type *S*. Typhimurium and *S*.Tm^*hilD*^. By reducing the initial fraction of *hilD* mutants in the inoculum as low as possible, we aimed to mimic spontaneous rise of these mutants at a low frequency. The first group remained under hygienic isolation (i.e., no co-housing) throughout the infection, while the second group got access to unperturbed microbiota via co-housing with a naïve CON^X^ mouse. The CON^X^ mice were added at day 4 p.i., when gut inflammation is still very pronounced (S2 Fig; [44]), but after the streptomycin has been washed out and the extremely bactericidal phase of the wild-type *S*. Typhimurium infection (between days 1 to 2 p.i. [48]) is over. We reasoned that this would promote efficient microbiota transfer and allow us to disentangle the effect of the microbiota from the inflammatory environment on the selection for *hilD* mutants. Strikingly, while the total *S*.Tm gut population remained at the carrying capacity for 40 days in both groups, only in the control group with no co-housing, the *hilD* mutant dominated the total pathogen population in the feces by day 40 p.i. (Fig 5B and 5C). In stark contrast, most of the mice that received microbiota transfer at day 4 p.i. were colonized by wild-type *S*. Typhimurium at the end of the infection (Fig 5B and 5C). Thus, a complex microbiota transfer during the acute phase of the enteric disease is sufficient to prevent the rise and accumulation of *hilD* mutants without compromising the gut colonization efficiency of the pathogen.

We next checked if the reason for lack of selection for *hilD* mutants in the co-housed group was attributable to absence of gut inflammation. Surprisingly, gut inflammation was apparent and comparably high in both groups (Fig 5D and 5E). Furthermore, pathogen loads in the systemic organs (mLN and spleen) were also comparable between both groups. Thus, the microbiota from a naïve donor mouse can effectively alleviate the selective advantage of *hilD* mutants during *S*. Typhimurium gut infection, even in the face of continued pronounced gut inflammation.

Altogether, we conclude that the gut milieu shaped by wild-type *S*. Typhimurium-triggered gut inflammation and supplemented by the transfer of complex microbiota prevents the selection for attenuated *hilD* mutants while permitting a robust gut-luminal growth of the pathogen.

### Long-term microbiota perturbation in mice infected with wild-type *S*. Typhimurium

Our data so far suggests that, under hygienic conditions, wild-type *S*. Typhimurium-triggered enteric disease in antibiotic pretreated mice selects for attenuated *hilD* mutants. This happens unless the mice are supplemented with an unperturbed microbiota from a naïve donor. On the other hand, enteric disease triggered by *S*.Tm* does not lead to attenuation of virulence since the regrowing microbiota is able to prevent the accumulation of *hilD* mutants. Therefore, we next investigated how the composition of the microbiota had changed during both infection conditions. To identify correlations between microbiota changes during wild-type *S*. Typhimurium and *S*.Tm* infection, the dynamics of *Salmonella* gut colonization, and the selection for mutants with reduced virulence, we performed 16S community sequencing on fecal samples taken from mice in Fig 1A–1D. It should be noted that the experiment shown in Fig 1A–1D relied on the initial microbiota perturbation by streptomycin pretreatment; previous work had established that this disturbs the microbiota composition and reduces its diversity transiently [12,58–60]. However, antibiotic treatment alone can only suppress CR for 1 to 3 days. Unless virulence-elicited inflammation suppresses microbiota regrowth, *Salmonella* loads in the gut lumen will decline from more than 10^9^ to about 10^7^ CFU per gram cecum content by day 4 p.i. [12]. This had been established for the wild-type *S*. Typhimurium infection and for the infection with the avirulent double-mutant lacking functional TTSS-1 and TTSS-2 (*S*.Tm^Avir^; see above) that did not cause any enteropathy [12]. Here, we sought to assess if the *S*.Tm* infection and the associated gut-luminal milieu allows the regrowth of microbiota at day 20 to 70 p.i. We reasoned that such data might help explain why *hilD* mutant-dominated *Salmonella* populations that arise transiently during *S*.Tm* infections are displaced and why that gut microbiota retains its capacity to displace *hilD* mutant-dominated pathogen populations from wild-type *S*. Typhimurium infected mice.

For the analysis, we assessed fecal microbiota composition at day 0 and compared these to the fecal microbiota composition at day 10 p.i. We chose day 10 p.i., as *Salmonella* loads were at carrying capacity in both groups of infected mice (≈10^9^ CFU/g feces; Fig 1B), concentrations of the inflammation marker lipocalin-2 were elevated and roughly equal (≈10^4^ ng/g feces; Fig 1C), and clones without detectable SipC expression were still a minority in the feces from most of the mice (Fig 1D). We also assessed day 70 p.i., as fecal *Salmonella* loads and gut inflammation differed strongly between both groups of mice (Figs 1B, 1C, and S3). Moreover, by day 70, the clones without detectable SipC expression dominated the feces of all mice infected with wild-type *S*. Typhimurium (but absent from *S*.Tm* infected mice; Fig 1D). Also, the day 70 p.i. data would be particularly valuable, as mice from that time point were used in the microbiota transfer experiment shown in Fig 3.

**Fig 6:**
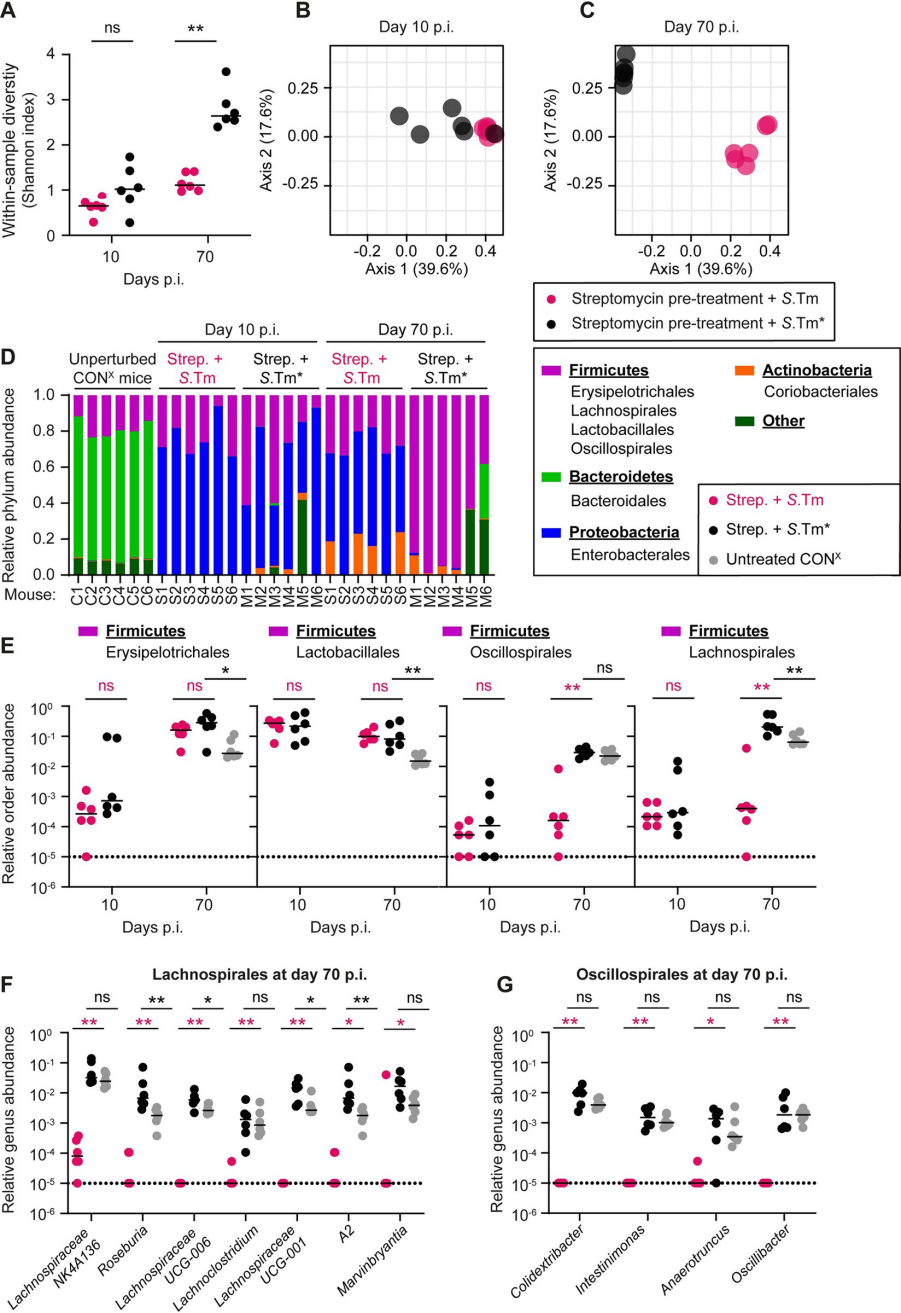
Long-lasting effect of wild-type *S*. **Typhimurium on the gut microbiota composition of streptomycin pretreated CON**^**X**^ **mice.** 16S sequencing was performed to analyze the microbial community in fecal samples from unperturbed CON^X^ mice sampled at day 10 and day 70 p.i. in the experiment shown in Fig 1B–1D. (A) Within-sample diversity measured using Shannon Index (pink circles: wild-type *S*. Typhimurium infection; black circles: *S*.Tm* infection). (B, C) Principal coordinate analysis based on Bray–Curtis dissimilarities between samples (after the square-root transformation of abundances). Data points represent individual mice, and a colored border defines the grouping of data points within each sample group. (PERMANOVA R^2^ = 0.397 and *p* = 0.0045 for day 10 p.i. and R^2^ = 0.661 and *p* = 0.0024 for day 70 p.i. (D) Relative abundances ASVs at the phylum level. The 4 most abundant phyla are shown, with the rest of the community shown as “other”. (E) Relative abundances of most abundant orders belonging to the phylum of Firmicutes. Orders were compared at day 10 and 70 p.i. Fecal microbiota from unperturbed CON^X^ mice (gray circles), which served as donors in Fig 3, were used as controls. (F, G) Relative abundances of most abundant genera belonging to the orders of Lachnospirales and Oscillospirales, respectively. Genera were compared at day 10 and 70 p.i. between wild-type *S*. Typhimurium and S.Tm*-infected mice; fecal microbiota from unperturbed CON^X^ mice (gray circles) were used as controls. (E–G) Dotted line indicates the detection limit. Lines indicate the median. Two-tailed Mann–Whitney U tests were used to compare wild-type *S*. Typhimurium to *S*.Tm*-infected samples (Panel A black stars, Panel E–G pink stars) or *S*.Tm* infected to untreated CON^X^ mice (Panel E–G; black stars) (*p* ≥ 0.05 not significant (ns), *p* < 0.05 (*), *p* < 0.01 (**), *p* < 0.001 (***), *p* < 0.0001 (****)). Source data can be found in S1 Data file.

At day 10 p.i., the within-sample diversity (as quantified by the Shannon index) was similar between wild-type *S*. Typhimurium and *S*.Tm*-infected mice, and lower than in naïve CON^X^ mice (compare Fig 6A to the gray symbols in Fig 3E). While the within-sample diversity remained low in wild-type *S*. Typhimurium infections, it returned almost to “unperturbed” levels in *S*.Tm*-infected mice by day 70 p.i. (Figs 6A and S13; compare to the gray symbols in Fig 3E). In *S*.Tm*-infected mice, the rise of microbiota complexity between days 10 to 70 p.i. correlated temporally with the decline of the total *Salmonella* loads in the feces (Fig 1B) and the decline of clones without detectable SipC expression (between days 20 to 70 p.i.; Fig 1D). Importantly, microbiota compositions were distinct between wild-type *S*. Typhimurium- and *S*.Tm*-infected mice at both time points, as shown by principal coordinate analysis (PCoA) based on Bray–Curtis dissimilarities (Fig 6B and 6C). Of note, identical observations were made when we excluded the *Salmonella* reads from this analysis. Thus, *S*.Tm* caused a transient reduction of the microbiota complexity that was restored despite the hygienic isolation during these 70 days of infection. In contrast, the impact of wild-type *S*. Typhimurium on microbiota complexity was distinct and long lasting.

To identify taxa that might be responsible for displacing mutant-dominated gut-luminal *Salmonella* populations, we first investigated the gut microbiota communities at higher taxonomic resolution. The unperturbed CON^X^ microbiota consists of 4 major phyla: Firmicutes, Bacteroidetes, Proteobacteria, and Actinobacteria (Fig 6D). Our analysis of microbiota compositions over time revealed striking differences in the relative abundance of taxa between the mice infected with wild-type *S*. Typhimurium or *S*.Tm*. The members of the phylum Proteobacteria were prominent in the *S*.Tm*- and wild-type *S*. Typhimurium infected mice at day 10 p.i. and >99% of the proteobacterial 16S sequences were attributable to the *Salmonella* strains used for infecting these mice (S7–S10 Tables). In line with the high fecal *Salmonella* loads (Fig 1B), the Proteobacteria sequences remained prominent until day 70 in the wild-type *S*. Typhimurium-infected mice, while they declined drastically in *S*.Tm*-infected animals (Fig 6D; blue bars; day 70 p.i.).

The phylum Bacteroidetes was suppressed by day 10 p.i. in both groups of mice (Figs 6D and S13E). Bacteroidales were the major order of this phylum in our mice and declined from a relative abundance of 69% (based on the 16S sequence reads from the feces of untreated CON^X^ mice; median) to about 0.1% and remained low until day 70 p.i. in both groups (Figs 6D and S13E). This suggested that the composition of the gut microbiota does not completely return to its initial state even in *S*.Tm*-infected mice. Furthermore, these data suggest that the Bacteroidales are likely not required for displacing the luminal *Salmonella* populations in our experiments.

The phylum Actinobacteria was present in low abundance in unperturbed animals and appeared at more variable abundances in wild-type *S*. Typhimurium and *S*.Tm*-infected mice at day 70 p.i. (Fig 6D). This pattern of abundance may suggest that the presence or absence of high Actinobacteria loads will likely have little effect on the gut colonization by *hilD* mutants.

In contrast to the Bacteroidetes, members of the phylum Firmicutes remained well-represented accounting for 10% to 30% of all 16S sequences at day 10 p.i. in most animals from both groups (Fig 6D). By day 70 p.i., the Firmicutes accounted for up to >95% of all 16S sequences in the *S*.Tm*-infected mice, while they remained at approximately 10% to 30% in the wild-type *S*. Typhimurium-infected animals. At higher taxonomic resolution, there were pronounced differences between the infected and the non-infected mice, and also between the groups infected with *S*.Tm* and wild-type *S*. Typhimurium (Fig 6E–6G). At the order level, we observed 3 patterns of change in response to the infection with *S*.Tm* or wild-type *S*. Typhimurium: (i) orders featuring an increased abundance in mice infected with either *S*.Tm* or wild-type *S*. Typhimurium at day 70 p.i.; (ii) orders that declined in both groups by day 10 p.i., but regrew by day 70 p.i. in the *S*.Tm*- (but not the wild-type *S*. Typhimurium-) infected mice; (iii) orders declining by day 10 p.i. but blooming to higher abundance than in the unperturbed microbiota by day 70 specifically in the *S*.Tm*-infected mice (Figs 6E and S13H). Taxa showing the latter 2 response patterns were of particular interest, as they might include key strains that displace *Salmonella* spp. and create an environment that selects against mutant overgrowth, as observed in the *S*.Tm*-infected mice (day 70, Fig 1B and 1D) or after microbiota transfer (Figs 3B, 3C, 4B and 4C).

The orders Oscillospirales and Lachnospirales were depleted by day 10 of infection with either strain, but regrew by day 70 p.i. with *S*.Tm*-, but not in the *S*.Tm-infected mice (Fig 6E). The underlying mechanism has remained unclear. On the one hand, the luminal environment of the convalescent gut (as in *S*.Tm*-infected mice at day 70 p.i.) might select for Oscillospirales and Lachnospirales. They may thus represent indicator strains for that gut-luminal milieu. Alternatively, the dominance of the Oscillospirales and Lachnospirales might hint at a more active function of these taxa in suppressing gut luminal *Salmonella* growth and selecting against attenuated *Salmonella* mutants when acute colitis resolves. In principle, such an “active” role might be exerted via short chain fatty acids (SCFAs) such as butyrate, as these microbiota metabolites have much higher concentrations in the cecum lumen of mice with high CR (such as CON^X^ mice), than in mice without CR (such as germ-free mice; S14 Fig). SCFA modulate the expression of the HilD-regulon [61–63], and Oscillospirales and Lachnospirales were shown to produce such metabolites. Regardless, even in the *S*.Tm*-infected mice, the microbiota composition did not return to the naïve state, as the Bacteroidales order was mostly replaced by orders from the Firmicutes phylum (mainly Lachnospirales; Figs 6D, 6E, and S13E), hinting that the post-colitis microbiota might have distinct functional features (with distinct effects on virulence regulation by enteropathogens, altered SCFA outputs, etc.) than the naïve microbiota.

To identify genera of the normal mouse gut microbiota that might be responsible for restricting long-term colonization by *hilD*-mutants, we investigated the orders Oscillospirales and Lachnospirales at the suborder level (Fig 6F and 6G). We compared the most abundant taxa in the gut of mice that had recovered from *S*.Tm* infection at day 70 p.i. with those of mice infected for 70 days with wild-type *S*. Typhimurium and with untreated mice (Fig 6F and 6G). Of note, Lachnospiraceae NK4A136, *Roseburia*, Lachnospiraceae UCG-006, *Lachnoclostridium*, Lachnospiraceae UCG-001, *A2*, *and Marvinbryantia* from Lachnospirales, and *Colidextribacter*, *Intestinimonas*, *Anaerotruncus*, and *Oscillibacter* from Oscillospirales were significantly elevated in mice which had recovered from *S*.Tm* infection (Fig 6F and 6G). We hypothesized that particular members or consortia that include groups of these taxa may create a gut-luminal environment selecting against *hilD* mutants (Fig 6F and 6G). To test this hypothesis, we focused on taxa that were initially depleted, but regrew by day 70 p.i. with *S*.Tm*. To this end, we chose cultivatable strains from Lachnospirales, Oscillospirales, and Erysipelotrichales.

In a first experiment, we infected streptomycin pretreated CON^X^ mice and inoculated these animals at days 0, 1, and 3 with the indicated microbiota strain (S15A and S15B Fig). This timing of microbiota inoculation seemed reasonable, as streptomycin is washed out of the gut within approximately 24 to 36 h, and *S*. Typhimurium gut infection has a pronounced effect on the gut-luminal bacterial community between days 1 and 2 p.i. [48,49], which starts to recover after day 2. However, neither the introduction of individual strains nor the supplementation of propionate or butyrate in the drinking water of mice (as examples for prominent microbiota fermentation products) prevented the selection for *S*.Tm*^hilD^ (S15B Fig).

A second experiment was performed in germ-free mice, as these animals lack any microbiota selecting against *hilD* mutants or displacing *hilD* mutant-dominated *Salmonella* populations from the gut lumen (see Fig 4B and 4C). Thus, adding suitable microbiota strains may restore these microbiota-mediated functions. To this end, we took competitively infected germ-free C57BL/6 mice (100:1 mixture of *S*.Tm* versus *S*.Tm* ^*hilD*^) from Fig 4 (at day 40 p.i.; green symbols), and inoculated these mice with a microbiota strain cocktail composed of *Actualibacter muris* KB18, *Clostridium clostridioforme* YL32, *Flavonifractor plautii* YL31, *Blautia coccoides* YL58, and *Faecalibaculum rodentium* DSM 103405 at days 40, 45, or 50 p.i. (S16A Fig). However, the microbiota inoculations did not affect the dominance of *S*.Tm* ^*hilD*^ (S16B Fig). The reasons for this remain unclear. We speculate that the 16S data from Figs 6 and S13 might have been insufficient to identify strains that can establish the proper gut-luminal environment. Alternatively, such environmental conditions may be an emergent property requiring a particular combination of microbiota strains (including strains which we have failed to add to our microbiota mix). Also, we cannot rule out that the Oscillospirales, Lachnospirales, or Erysipelotrichales do not play an active role in selecting against *hilD* mutants or displacing mutant-dominated *Salmonella* populations. Instead, they may just be indicator strains which dwell in the gut-luminal milieu of a convalescent mouse. These questions will be a topic for future work.

In summary, our data suggested that emergent properties of the full post-recovery community might be responsible for suppressing the spread of *hilD* mutants in *S*.Tm*-infected mice (Fig 1B and 1D) and after microbiota transfer into wild-type *S*. Typhimurium-infected animals (Fig 4B and 4C).

### Microbiota conferring intermediate levels of CR tend to prevent selection for *hilD* mutants

The data above suggested that the gut microbiota is critical for conditioning a gut luminal milieu that selects against *hilD* mutants. The superior gut-luminal colonization by wild-type *S*. Typhimurium (compared to *S*.Tm^*hilD*^) was particularly evident after a mild diet-mediated microbiota perturbation (Figs 2 and S6) and in mice harboring microbiotas which confer lower levels of CR compared to the unperturbed complex microbiota (that is CON^X^ or CON^E^). In those initial experiments, we had infected the mice with either wild-type *S*. Typhimurium or with *S*.Tm^*hilD*^ alone. However, it remained unclear if wild-type virulence would be selected for or against in competition with a *hilD* mutant in the presence of a microbiota that conferred intermediate CR. Therefore, we performed 2 competitive infection experiments in mouse models with intermediate CR.

**Fig 7:**
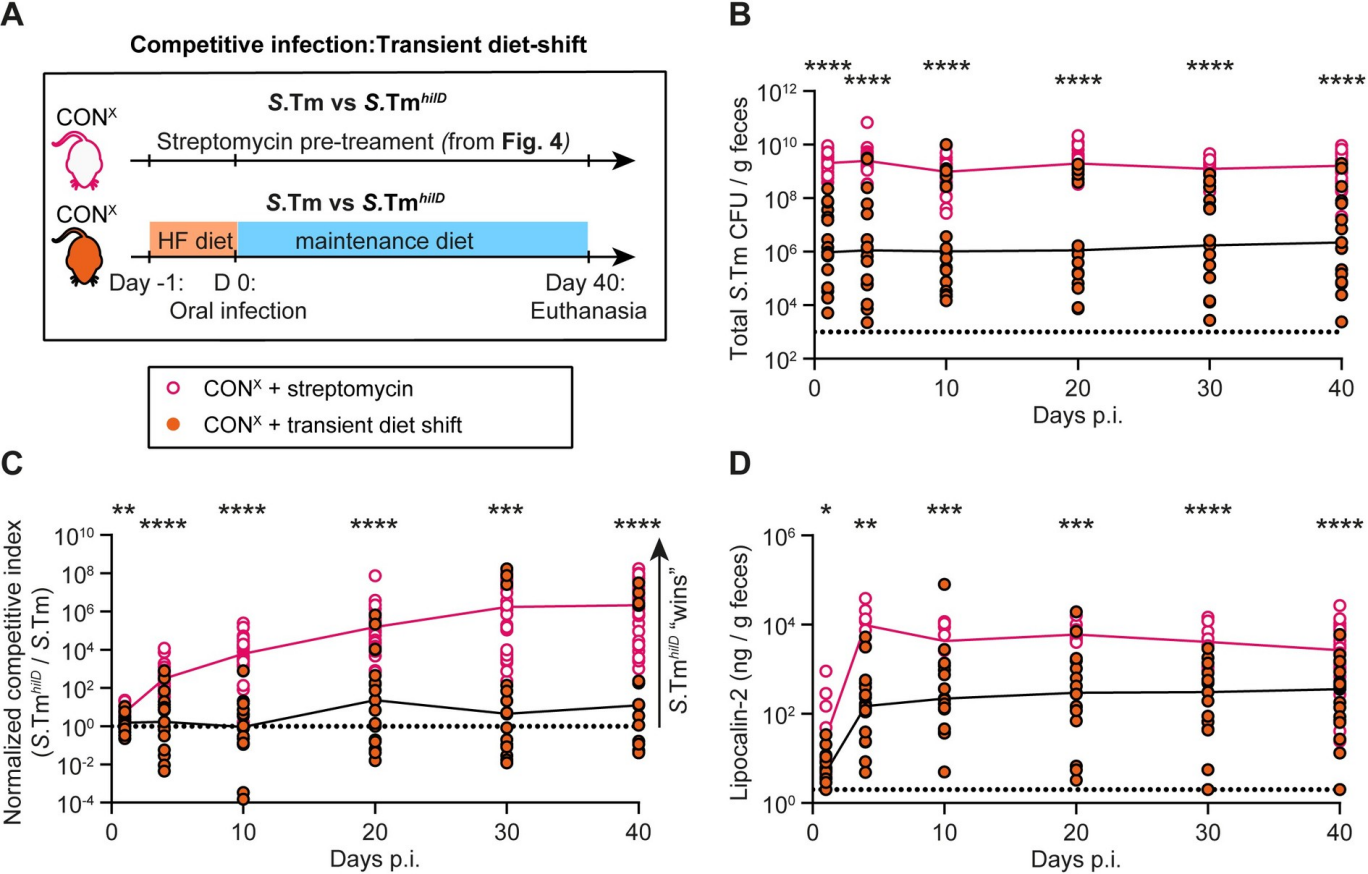
After high-fat diet shift, selection for *hilD* mutants is reduced compared to streptomycin pretreated mice. (A) Experimental scheme. (B–D) CON^X^ were shifted from their normal plant based diet to a high-fat diet for the day before the infection (and switched back to the normal diet) with a 100:1 mixture of wild-type *S*. Typhimurium vs. *S*.Tm^*hilD*^ (orange symbols; 5 × 10^7^ CFU, by gavage; *n* = 17 mice 2 independent experiments). The equivalent data for streptomycin pretreated mice is shown as a control (black symbols; re-plotted from Fig 4A). (B) Total *Salmonella* loads detected in the feces by selective plating. Dotted lines indicate the detection limit. Colored lines connect the medians. (C) Normalized C.I. as determined using MacConkey plates with selective antibiotics for wild-type *S*. Typhimurium vs. *S*.Tm^*hilD*^. The dotted line indicates a C.I. of 1. (D) An ELISA for fecal lipocalin-2 was used to compare gut inflammation between the 2 groups. The data shown was obtained from 2 independent experiments including comparing both groups. Two-tailed Mann–Whitney U tests were used for statistical analysis (*p* ≥ 0.05 not significant (ns), *p* < 0.05 (*), *p* < 0.01 (**), *p* < 0.001 (***), *p* < 0.0001 (****)). Source data can be found in S1 Data file.

In the first experiment, we competed wild-type *S*. Typhimurium against *S*.Tm^*hilD*^ in CON^X^ mice that had been exposed to a high-fat diet shift to transiently alleviate CR (Fig 7A). We hypothesized that this mouse model might offer conditions that prevented selection for *hilD* mutants since our data already indicated that such a moderate disturbance of a complex microbiota might condition the intestinal milieu to favor virulence (S6J Fig). To this end, the CON^X^ mice were shifted to high-fat diet for 24 h before infection with a 100:1 mixture of wild-type *S*. Typhimurium versus *S*.Tm^*hilD*^ and we analyzed gut luminal growth of the *Salmonella* strains and fecal lipocalin-2 concentrations for 40 days. The median total *Salmonella* loads in the feces remained at ≈10^6^ CFU/g throughout our experiment, which is higher than expected for mice with strong CR, like unperturbed CON^X^ mice (S6K Fig), but significantly lower than in streptomycin pretreated CON^X^ mice (Fig 7B, replotted from Fig 4A). In these high-fat diet shifted CON^X^ mice, the average selection for the *hilD* mutant was extremely mild or absent (Fig 7C). In fact, in some animals, we observed selection for the *hilD*-proficient wild-type *S*. Typhimurium strain. This selection for wild-type *S*. Typhimurium appeared to be most pronounced in those animals with the lowest gut luminal pathogen loads (10^3^–10^7^ CFU per g feces). To assess this in more detail, we re-analyzed the infection data separately for the subgroups of mice featuring total fecal *Salmonella* loads of >10^6^ CFU/g or of <10^6^ CFU/g at day 10 p.i. (Figs 7 and S17A–S17D). In the former subgroup, the *S*.Tm^*hilD*^ was selected for strongly (C.I. ≈10^6^ by days 30 to 40 p.i.). In contrast, the latter subgroup did not select for *S*.Tm^*hilD*^ (C.I. ≈1 at days 1 to 40 p.i.; S17C Fig, gray circles). This split into 2 distinct subgroups might be related to the high animal-to-animal variability rooted in the relatively mild effect of the high-fat diet shift on CR (as compared to the streptomycin pretreatment) which results in a high animal-to-animal variation in gut colonization and the degree of enteropathy (which was also observed above (S6E and S6J Fig) and in earlier work [25]). We hypothesize that in those animals with fecal *Salmonella* densities of <10^6^ CFU/g, the microbiota was only minimally disturbed: just enough to partially alleviate CR but too little to fully alleviate the microbiota’s capacity to condition the gut luminal milieu and thereby facilitate selection for *S*.Tm^*hilD*^. Strikingly, the selection for *S*.Tm^*hilD*^ was prevented in this subgroup of mice, despite significant levels of gut inflammation as indicated by fecal lipocalin-2 concentrations of ≈10^2^ ng/g from days 3 to 40 p.i. (Figs 7 and S17D, gray symbols). This is in keeping with the data in Fig 5 and suggests that the parts of the microbiota that are critical for conditioning the selective gut-luminal milieu remained intact in that subgroup of mice and that this can prevent the selection for *hilD* mutants even in the inflamed gut.

In the second experiment, we analyzed the competitive infection of *hilD*-proficient versus -deficient *Salmonella* strains in gnotobiotic C57BL/6 mice, which are associated with a low complexity microbiota (termed LCM). The LCM mice harbor a microbiota consortium composed of 8 strains and confer an intermediate level of CR. Upon orogastric infection in this mouse model, wild-type *S*. Typhimurium takes 3 to 4 days to grow to carrying capacity and 2 to 4 days to elicit pronounced enteropathy [4,46,47], and our new data shows that a *hilD* mutant is attenuated (S6C and S6H Fig). In order to assess the selection for (or against) virulence over 40 days, we used an isogenic strain pair that carries a *ssaV* mutation, as this prevents death from systemic spread (as discussed above). The LCM mice were infected with a 100:1 mix of *S*.Tm* and *S*.Tm*^hilD^ to allow the virulent strain *S*.Tm* to condition the inflammatory milieu in the gut lumen during the first days of the infection and to approximate the low abundance of *hilD* mutants that spontaneously arise in natural *Salmonella* populations (S18 Fig). In the LCM mice, *S*.Tm*^hilD^ was selected for during the first 10 days, as indicated by the C.I. which rose from 10^0^ to ≈10^2^ (S18D Fig). This period was associated with mild gut inflammation, as indicated by fecal lipocalin-2 concentrations of ≈10^2^ ng/g feces (S18E Fig). Importantly, the C.I. of *S*.Tm*^hilD^ dropped by >10,000-fold between days 10 and 40 of the experiment (S18D Fig), when lipocalin-2 concentrations returned to normal levels as typically observed in unperturbed mice. At the same time, the gut-luminal densities of the *hilD*-proficient strain remained very high (≈10^9^ CFU/g feces; S18C Fig). Thus, the gut-luminal environment in LCM mice strongly selected for the virulent strain, at least during days 10 to 40 of our experiment.

Together, these data support the hypothesis that the gut microbiota is critical for conditioning the milieu in the infected gut and that microbiota conferring intermediate levels of CR may tend to select for wild-type *S*. Typhimurium virulence.

## Discussion

We studied wild-type *S*. Typhimurium infections in mice to decipher the role of the microbiota in virulence evolution of enteropathogens. Within-host evolution, microbiota transfer and infection assays in mice with different levels of CR established that the gut microbiota has a key role in conditioning the gut-luminal milieu with important consequences for the selection for mutants with reduced virulence. If the microbiota is absent or disrupted beyond recovery (Figs 1, 3, 4, 6 and S16), virulence-attenuated *hilD* mutants replace the parental wild-type strain, in particular in the inflamed gut. Thereby virulence declines. This is prevented if the microbiota recovers during convalescence (Figs 1, 3, 4 and S18), or if it is replenished by transfer (Figs 3–5). Overall, this identifies a trade-off between the costs of *hilD*-dependent virulence expression, its role in disturbing the gut microbiota, and the role of the microbiota in preventing selection for *hilD* mutants, which determines the within-host evolution of wild-type *S*. Typhimurium. We hypothesize that this gut milieu may represent the natural habitat which has selected for the evolution and the maintenance of virulence by this enteropathogenic bacterium. Notably, most natural *Salmonella* isolates retain a functional HilD-regulon [41,64]. Hence, we propose that using mouse infection models selecting for the retention of the HilD-regulon may allow us to optimize our experimental approaches and identify conditions similar to the pathogen’s natural habitat. These conditions could serve as a testing ground to discover vulnerabilities of the pathogen. Unraveling how microbiotas shape the gut environment that favors virulence may lead to therapies and preventive measures that restore the microbiome and reinstate CR without promoting virulence (Supplementary discussion B in S1 Text).

Inflammation is a critical parameter conditioning the milieu in the infected gut [4]. Inflammation has 2 inter-related effects on the pathogen population. One is that it fuels the selection of *hilD* mutants over wild-type *S*. Typhimurium. Without gut inflammation, the *hilD* mutant and the isogenic wild-type strain grow equally well in the antibiotic pretreated mouse gut lumen (S11 Fig). The second is that gut inflammation disrupts the microbiota, which allows the pathogen to grow at higher densities over longer periods of time. This is true for wild-type *S*. Typhimurium, as initially discovered in [12] (though without noticing the *hilD* mutants) and confirmed by the present study. Our new data show that this is also true for *S*.Tm^*hilD*^ (Figs 1 and 4). The microbiota disruption beyond return (as triggered by the enteric disease during the first days of a wild-type *S*. Typhimurium infection) explains why *hilD* mutant-dominated pathogen populations can populate the gut over long times (as in Fig 1). Both effects are interrelated, as both (microbiota disruption and the selection for *hilD* mutants) are mediated by the gut-luminal milieu of the inflamed gut and as the microbiota can condition the inflamed gut in a way that halts the selection for *hilD* mutants (Fig 5).

Wild-type *S*. Typhimurium infection in antibiotic pretreated mice represents an extreme case of microbiota disruption during enteric infection, and this helped to uncover 2 phenomena important for within-host evolution of the pathogen: Under these extreme conditions, the high severity of enteropathy of the wild-type pathogen can lead to short-sighted evolution towards virulence attenuation (Figs 1, 2, S5 and S6) as indicated by the selection for attenuated mutants and their massive fecal shedding over weeks and months (Figs 1B, 1D, 1E, 3B and 3C). This is strikingly different from our earlier observations in *S*.Tm*-infected mice and the experimental controls in our present study, wherein the regrowing gut microbiota halted the evolution towards reduced virulence [29]. Second, as shown here, the extreme conditions triggered by the wild-type pathogen could identify a trade-off between virulence-driven alleviation of microbiota-mediated CR and the key role of the microbiota in conditioning a gut-luminal milieu that selects for the maintenance of *Salmonella* virulence. In line with earlier work [12], the pronounced enteropathy elicited by wild-type *S*. Typhimurium in streptomycin pretreated mice is a prerequisite for long-term gut colonization of mice with complex gut microbiotas. Therefore, as long as host viability remains unchanged, higher virulence would be expected to correlate with enhanced transmission, and this should select for the evolution of virulence or its maintenance by the wild-type pathogen. However, our new data presented here show that the pronounced disruption of the microbiota by wild-type *S*. Typhimurium (“beyond return”; Figs 6 and S13) results in the selection for attenuated mutants (Figs 1, 4, S5, S6, S8 and S12). Together, these data demonstrate the trade-off between virulence-triggered microbiota suppression and the virulence-selecting effect of the microbiota. In other words, “more inflammation” is not always “better” when considering the maintenance of virulence in *Salmonella* populations. The key role of the microbiota in promoting the selection for virulence and therefore coincidentally preventing short-sighted evolution for virulence loss in pathogens is best illustrated by our microbiota transfer experiments after 40 or 70 days of infection with wild-type *S*. Typhimurium (Figs 3 and 4), and by the transfer experiment in which we co-housed mice at day 4 of the infection (Fig 5). Additional support for the role of the gut microbiota in selecting for the maintenance of virulence comes from the gut colonization defects of *hilD* mutants in mice with intermediate levels of CR (Figs 2 and S6). In hosts with transient food-mediated disturbance of CR, microbiotas conferring intermediate levels of CR, and in cases where pronounced enteropathy is combined with microbiota transfer, the pathogen can exploit virulence-elicited inflammation to (partially) suppress the microbiota and accelerate *Salmonella* growth, without selection for virulence-attenuated mutants. We hypothesize that this would promote transmission and thereby enhance the fitness of the wild-type pathogen [65]. During spread in a host population, on the other hand, additional factors come into play in the selection for *Salmonella* virulence. For example, the transmission bottleneck between hosts selects for virulent *S*.Tm cells that are able to trigger gut inflammation in the next host [52,66], as attenuated mutants will be unable to. We believe that our results from within-host evolution experiments introduce another layer to this concept: unperturbed or regrowing microbiota selects against attenuated mutants and thereby increases the total number of wild-type pathogen cells that are shed into the feces and can infect new hosts. This process would eventually increase the epidemiological fitness of the virulent *S*.Tm population in nature [67]. Consequently, within-host selection of virulence, which is promoted by microbiota, can contribute to the evolution of virulence within-population. However, additional data should be obtained to assess the potential of the pathogen to spread across host populations (that is fitness at evolutionary timescales) in a quantitative fashion.

The key role of the gut microbiota in preventing the selection for *hilD* mutants is further supported by our new data from germ-free mice. In contrast to our previous work in antibiotic pretreated CON^X^ or CON^E^ mice, *S*.Tm*^hilD^ was selected for over the *hilD*-proficient strain (that is *S*.Tm*) in germ-free animals (Fig 4, green symbols). Moreover, the mutant-dominated *Salmonella* population was maintained and shed at high densities over many weeks. Our data suggest that this striking difference to streptomycin pretreated CON^X^ mice resides in the failure of *S*.Tm* to disrupt the complex gut microbiota beyond return (Figs 6 and S13) and indicate that this is attributable to the reduced degree of enteropathy that is elicited by *S*.Tm* compared to wild-type *S*. Typhimurium (S2 Fig). This reduced virulence of *S*.Tm* has also been well documented in previous work [20,23]. These observations provide additional support for the trade-off between the virulence-triggered microbiota suppression and the virulence-selecting effect of the microbiota. In face of a complex microbiota (and its transient alleviation by antibiotic treatment, diet shifts, etc.), moderate enteropathy can also entail the transient appearance of attenuated mutants. However, these are later displaced by regrowing microbiota (see *S*.Tm* data in Figs 1, 4 and 6), as key members of the microbiota have survived the disease.

What else can we learn from the antibiotic pretreated mouse model and its tendency to select for HilD-regulon mutants? A body of previous work shows that it represents an extreme but highly instructive model for deciphering inflammation-triggering mechanisms [68–71], studying *Salmonella* growth under full-blown inflammation [12,31,32], and assessing why HilD-regulon expression can be so costly at times of severe microbiota perturbation [29,38,52]. These costs explain why mutants with HilD-regulon defects become dominant in *Salmonella* populations when the host’s microbiota is disrupted by the combined action of antibiotics plus wild-type *S*. Typhimurium infection and when microbiota transfer is prohibited by hygienic isolation (Figs 1 and 4). The spectrum of mutations is strikingly similar to those observed in the genome sequences of natural *Salmonella enterica* isolates [41]. This suggests that the antibiotic pretreated mouse model is representative of a distinct (though small and poorly characterized) natural niche characterized by a milieu selecting for HilD-regulon mutants. In the majority of hosts, the microbiota confers either strong CR (preventing colonization and evolution towards reduced virulence) or intermediate levels of CR, selecting for an intact HilD-regulon [41]. Even after the pronounced microbiota disruption by antibiotics plus wild-type *S*. Typhimurium infection, microbiota transfer can terminate the selection for *hilD* mutants (Fig 5) or the gut colonization by mutant-dominated pathogen populations (Figs 3 and 4). Such microbiota transfer should occur in most natural infections. This raises the question about the nature of the natural niche that selects for mutants with reduced virulence, which had been observed by Joshua Cherry [41]. So far, this niche has not been identified. Our data suggest that rare incidences of an extreme disruption of the mature complex microbiota (e.g., by food, antibiotics, and/or massive inflammation) and a limited access to microbiota transfer by strict hygiene measures might explain some of these cases. Evidence from an infant mouse infection model [72] (which is known to select for flagella mutants over wild-type *S*. Typhimurium) [73] indicates that incomplete CR provided by infant gut microbiota might also select for mutants with reduced virulence. However, further work is needed to substantiate the relevance of these niches or identify more relevant niches selecting for reduced *Salmonella* virulence.

How does enteropathy affect the gut microbiota? Interestingly, the degree of enteropathy appears to determine how microbiota communities shift (Figs 6 and S13). Compared to *S*.Tm*, the pronounced enteropathy elicited by wild-type *S*. Typhimurium entails more neutrophil recruitment into the gut lumen, exacerbated epithelial damage, altered metabolite levels in the gut lumen, and altered spatial organization of the cecal microenvironment (S2 and S14 Figs). Previous work established that the enteropathy elicited by wild-type *S*. Typhimurium in streptomycin pretreated mice not only prevents gut microbiota regrowth, but also it has particularly strong bactericidal effects at days 1 to 2 p.i. [48,49], which are strong enough to reduce the gut-luminal loads of the inflammation-adapted pathogen by 10 to 10,000-fold at day 2 of the infection [48,49], before it can regrow to carrying capacity (≈10^9^ CFU/g in cecum content or feces). The co-housing experiment in Fig 5 provides further evidence that microbiota can (re)-establish in the infected gut as early as day 4 p.i. These observations suggest that days 1 to 2 are most critical for the disruption of the microbiota beyond return in wild-type *S*. Typhimurium-infected mice. We hypothesize that tissue-destructive inflammation marked by elevated IFN-γ levels might disrupt microbiota preserving niches in the gut mucosa like crypts, Peyer’s patches, or mucus-associated communities [74]. Our experiments were limited to longitudinal sampling of gross fecal microbiota composition, rather than assessing subtle differences in spatial organization. It was suggested that differences in sampling technique might result in underrepresentation of tissue-associated bacteria [75]. Further analysis of the relevant microbiota members in these niches and the underlying molecular mechanisms facilitating their long-term suppression or regrowth may broaden our understanding of specific functions of particular microbiota strains and be of interest as biotherapeutics, e.g., to reduce an infection-related predisposition, or alleviate auto-inflammatory sequelae like in IBD (see Supplementary discussion C in S1 Text). Moreover, this could highlight specific competitive effects of different microbiota members which may condition the gut to reverse colonization by enteropathogenic bacteria (to be used as probiotics). However, care must be taken to avoid microbiota members that may coincidentally select for *Salmonella* virulence.

Multiple studies have reported the evolution of microbial symbionts from mutualism to parasitism or virulence and vice versa, dependent on host associations or the environmental context (reviewed in [7,9]). Previous work on the fungal pathogen *C*. *albicans* had established a critical role of the microbiota in selecting against mutants with impaired hyphal virulence [10,11,76–78]. Here, we demonstrate the role of the microbiota in the maintenance of virulence by a wild-type enteropathogenic bacterium. In contrast to the *C*. *albicans* data, our work established that wild-type *S*. Typhimurium virulence has a dual relationship with the microbiota that results in a trade-off between virulence-mediated microbiota disruption and the role of the microbiota in maintaining *Salmonella* virulence. As many other gut pathogens such as *C*. *rodentium*, *V*. *cholerae*, *or C*. *difficile* use virulence-mediated host exploitation as a strategy to increase their transmission akin to *S*. Typhimurium, we suggest that the microbiota might play a similar role for the maintenance of virulence in such bacterial pathogens [12–16]. This both provides an experimental framework and opens up a major field of exploration to understand the role and mechanisms of how the microbiota influences the evolutionary dynamics of virulence across multiple enteric pathogens.

## Materials and methods

### Ethics statement

All mouse experiments were done according to the legal requirements and performed as approved by the responsible authority (Tierversuchskommission, Kantonales Veterinäramt Zürich, license approval numbers: ZH158/2019, ZH108/2022, and ZH109/2022). Sample size was not predetermined and mice were randomly assigned to treatment group. In co-housing experiments, female mice were predominantly used. In all other experiments, we used mice of 8 to 12 weeks of age of both sexes.

### Bacterial strains and growth conditions

In all experiments, *Salmonella* Typhimurium SL1344 (SB300, Sm^R^) or the indicated mutant versions were used (summarized in Table 1). Desired genetic constructs were transferred into the appropriate background strain using P22 HT105/1 int-201 phage transduction [79]. Antibiotic resistance cassettes were removed using the heat inducible FLP recombinase encoded on pCP20, if desired [80]. For in vivo mouse infections, bacteria were grown in lysogeny broth (LB) media containing the appropriate antibiotics (50 μg/ml streptomycin (AppliChem); 15 μg/ml chloramphenicol (AppliChem); 50 μg/ml kanamycin (AppliChem); 100 μg/ml ampicillin (AppliChem)) at 37°C for 12 h and subcultured in 1:20 LB without antibiotics for 4 h. Cells were washed and resuspended in cold PBS (BioConcept).

**Table 1:** Strains used in this study.

Strain name used in the study	Strain number	Relevant genotype	Resistance#	Reference
*S*.Tm (wild-type *S*.Tm)	SB300	Wild-type, *S*. Typhimurium SL1344	Sm	[81]
*S*.Tm^#^ (*S*.Tm *ssaV*)	M2730	SL1344 *ΔssaV*	Sm	[82]
*S*.Tm^Avir^ (*S*.Tm *invGssaV*)	M2702	SL1344 *ΔssaV*; Δ*invG*	Sm	[82]
*S*.Tm^*hilD*^ (*hilD* mutant)	T247	SL1344 *ΔhilD; aphT*	Sm, Kan	This study
*S*.Tm Cm^R^	T176	SL1344 *WITS*::*cat*	Sm, Cm	This study
*S*.Tm^#^^*hilD*^	T132	SL1344 *ΔssaV; hilD*::*cat*	Sm, Cm	This study
*S*.Tm^#^ Kan^R^	T156	SL1344 *ΔssaV*; *aphT*	Sm, Kan	This study
*S*.Tm^Avir *hilD*^	T134	SL1344 *ΔssaV*; Δ*invG; hilD*::*cat*	Sm, Cm	This study
*S*.Tm^Avir^	Z6832	SL1344 *ΔssaV*; Δ*invG; WITS*::*aphT*	Sm, Kan	This study

^#^ Relevant resistances only: Sm = ≥50 μg/ml streptomycin; Cm = ≥15 μg/ml chloramphenicol; Kan = ≥50 μg/ml kanamycin.

### Infection experiments

Infection experiments followed protocols derived from the streptomycin pretreated mouse model for *S*.Tm oral infection [19]. Experiments were performed in 8- to 12-week-old male or female mice. Different mouse models with varying CRs were used throughout the study. Germ-free (GF) mice have no CR [83]. LCM [46] and Oligo-MM^12^ [57] mice are ex-GF mice that are stably colonized by 8 and 12 representative microbiota species, respectively. They confer intermediate CR. Specified pathogen free 129/SvEv mice (CON^X^) and C57BL/6 (CON^E^) feature complete CR [4], unless pretreated with antibiotics, and 129/SvEv mice were used for *S*.Tm infections >4 days, since they contain a functional *Nramp1* allele (also known as *Slc11a1*). This allows resistance to systemic *S*.Tm disease and therefore allow for long-term infections [44,84]. For all infection experiments, the inocula were prepared as follows. Overnight cultures of *S*.Tm in LB with streptomycin were subcultured for 4 h (1:20 dilution) in LB without antibiotics. The subcultured strains were washed with PBS, and mice were given a dose of approximately 5 × 10^7^ CFU *S*.Tm by oral gavage. Feces were collected in pre-weighed tubes containing 1 ml PBS and homogenized with a steel ball for 2 min at 25 Hz using TissueLyser (Qiagen). Mice were euthanized at the time point indicated in the figure legends. The organs were collected aseptically, ensuring that dissection tools were disinfected with ethanol in between organ collection to minimize cross-contamination. The mesenteric lymph nodes (mLNs), spleen, liver, gall bladder, kidney, lung, brain, heart, and stomach are reported per organ. Approximately 100 μl of blood was aspirated from the heart immediately after euthanasia and collected in PBS with 2% BSA and 1 mM EDTA. All organs were homogenized in PBS with a steel ball for 3 min at 30 Hz.

### Single infections in streptomycin pretreated mice

Mice were pretreated with 20 mg streptomycin by oral gavage, and 24 h later, mice were infected with an inoculum of approximately 5 × 10^7^ CFU *S*.Tm by oral gavage.

### Competitive infections

Mice were pretreated with 20 mg streptomycin by oral gavage, and 24 h later, mice were infected with an inoculum consisting of a mix of the competing strains tagged with different antibiotic resistance markers in the chromosome (approximately 5 × 10^7^ CFU *S*.Tm total inoculum) by oral gavage. Competing strains were grown and subcultured individually and combined only after washing with PBS. The competitive index is calculated as the ratio between 2 strains, normalized by the ratio of the strains in the inoculum (as confirmed by selective plating).

### Single infections in western-diet shifted mice

Mice were switched to western diet (BioServ, S3282; 60% kcal fat; irradiated; per weight: 36% fat, 20.5% protein, 35.7% carbohydrates, 0% fiber) [25] from maintenance diet (Kliba Nafag, 3537; autoclaved; per weight: 4.5% fat, 18.5% protein, approximately 50% carbohydrates, 4.5% fiber), and 24 hours later, mice were switched back to maintenance diet and infected with an inoculum of approximately 5 × 10^7^ CFU *S*.Tm by oral gavage. Cages were changed in between diet switches to limit feeding on residual diet in the cage and carryover of microbiota.

### Fecal transmission experiments

Fecal suspensions from mice given *S*.Tm were stored in 20% glycerol/LB at −80°C until use (S6 Fig). Fecal suspensions were inoculated directly from the glycerol stock into LB containing streptomycin and allowed to grow for 5 h. Cells were centrifuged and washed with PBS twice, before introducing approximately 5 × 10^7^ CFU total into mice pretreated with 20 mg streptomycin 24 h prior.

### Fecal transmission experiments

Fecal pellets from mice given *S*.Tm were collected at day 3 p.i., resuspended in 1 ml PBS (S5 Fig). From this suspension, 100 μl was given to the next mouse by oral gavage.

### Co-housing experiments

Female mice infected with *S*.Tm to be co-housed were split into fresh individual cages (in 1 case a cage of 2 mice) at the time point of co-housing. Mice were randomly assigned into co-housing groups (or left in their original cage). Female mice to be use as co-housing donors were added to the cage (1 donor mouse per cage). Mice added to the cage were either untreated CON^X^ mice (no perturbation before addition to the cage) or infected with *S*.Tm^SPI-2^ for 40 days.

### Microbiota gavage experiments

Germ-free mice or streptomycin pretreated CON^X^ mice were gavaged via orogastric route with a cocktail of representative microbiota strains (frozen glycerol stocks; [85,86]). Oligo-MM^12^ strains were grown in anaerobic Akkermansia medium (AAM; 18.5 g/l brain heart infusion (BHI), 5 g/l yeast extract, 15 g/l trypticase soy broth, 2.5 g/l K2HPO4, 1 mg/l haemin, 0.5 g/l glucose, 0.4 g/l Na2CO3, 0.5 g/l cysteine hydrochloride, 5 mg/l menadione, 3% complement-inactivated fetal calf serum) anaerobically. The successful growth of the cultures was confirmed by OD measurements and 16S PCR. Strains from DSMZ (German Collection of Microorganisms and Cell Cultures GmbH) were ordered as active cultures (grown in their respective media according to DSMZ culture conditions) and glycerol stocks were prepared anaerobically.

### Immunofluorescence

Cecal tissue sections from mice were fixed with 4% paraformaldehyde, saturated in PBS containing 20% sucrose, and snap-frozen in optimal cutting temperature compound (OCT; Sakura USA). Samples were stored at −80°C until further analysis. Samples to be stained were cut in 10 μm cross-sections and mounted on glass slides (Superfrost++, Thermo Scientific). For staining, cryosections on the glass slides were air-dried, rehydrated with PBS, and permeabilized using a 0.5% TritonX-100 solution in PBS. Sections were blocked using 10% normal goat serum (NGS)/PBS before staining with primary and secondary antibodies. The following antibodies and dilutions were used for the staining of different samples: 1:200 EpCam/CD326 (clone G8.8, Biolegend), 1:200 α-*S*.Tm LPS (O-antigen group B factor 4–5, Difco), 1:200 α-Ki67 (ab15580, Abcam Biochemicals), or 1:200 α-Ly6B.2 (clone 7/4, BioRad) in combination with the respective secondary antibodies, i.e., α-rabbit-AlexaFluor488 (Abcam Biochemicals), α-rat-Cy3 (Jackson), fluorescent probes, i.e., CruzFluor488-conjugated Phalloidin (Santa Cruz Biotechnology), AlexaFluor647-conjugated Phalloidin (Molecular Probes), and/or DAPI (Sigma Aldrich). Stained sections were then covered with a glass slip using Mowiol (VWR International AG) and kept in dark at room temperature (RT) overnight. For Confocal Microscopy Imaging, a Zeiss Axiovert 200 m microscope with 10 to 100× objectives or a spinning disc confocal lased unit (Visitron) with 10 to 100× objectives were used. Manual quantification was done blindly on 2 different sections (3 to 5 regions per section) from the same mouse according to the indicated parameters. The number of neutrophils per 63× field of view was counted on epithelium where half of the field included the lumen close to the epithelium to include freshly transmigrated neutrophils.

### qRT-PCR

Cecal tissue sections were snap-frozen in RNAlater solution (Thermo Fisher Scientific) after extensive washing of the content in PBS and stored at −80°C until downstream analysis. Total RNA was extracted using RNeasy Mini Kit (Qiagen) and converted to cDNAs employing RT^2^ HT First Strand cDNA Kit (Qiagen). qPCR was performed with FastStart Universal SYBR Green Master reagents (Roche) and Ct values were recorded by QuantStudio 7 Flex FStepOne Plus Cycler. Primers used either were from Qiagen as pre-validated primer assays for *Cxcl9*, *il1a*, *Ccl2*, *Adgre1 (*F4/80*)*, or designed using the NCBI primer-designing tool (see Table 2). The mRNA expression levels were plotted as relative gene expression (2^-ΔΔCt)^) and comparisons are specified in the figure caption.

**Table 2:** Primer sequences used for real time qRT-PCR.

Gene name		Primer sequence (5`➔3`)
β-actin (mouse)	**F** **R**	AGAGGG1TCGTGCGTGAC CAATAGTGATGACCTGGCCGT
Cxcl1 (mouse)	**F** **R**	CCGCTCGCTTCTCTGTGC CTCTGGATGTTCTTGAGGTGAATC
Cxcl10 (mouse)	**F** **R**	GGAT6TCTCGCAAGGA ATCGTGGCAATGATCTCAACA
Ifng (mouse)	**F** **R**	TTCTTCAGCAACAGCAAGGC TCAGCAGCGACTCCTTTTCC
Il1β (mouse)	**F** **R**	GCAACTGTTCCTGAACTCAACT ATCTTTTGGGGTCCGTCAACT
Il6 (mouse)	**F** **R**	CCTCTGGTCTTCTGGAGTACC ACTCCTTCTGTGACTCCAGC
Reg3g (mouse)	**F** **R**	ATGGCTCCTATTGCTATGCC GATGTCCTGAGGGCCTCTT
Tnfa (mouse)	**F** **R**	ATGAGCACAG1GCATGA AGTAGACAGAAGAGCGTGGT

### Lipocalin-2 ELISA

Lipocalin-2 ELISA (R & D Systems) was performed on feces according to the manufacturer’s instructions. Fecal pellets were suspended in PBS, diluted 1:20, 1:400, or undiluted, and the concentrations were determined by curve fitting using a Four-Parametric Logistic Regression in GraphPad Prism version 7.

### Colony protein blots

A colony protein blot for SipC (as a proxy of TTSS-1 expression) was used to quantify the proportion of clones that can express SipC in the feces. For a detailed protocol, see [50]. Colonies on MacConkey agar from diluted fecal suspensions were replica transferred to nitrocellulose membranes, placed face-up onto LB agar without antibiotics and allowed to grow at 37°C overnight. The original MacConkey plates were also re-incubated and then stored at 4°C. A series of Whatman filter papers soaked with buffers were used to lyse colonies and hybridize cellular material to the membrane. Membranes were placed on Petri dishes containing soaked Whatman filer papers in the following conditions: 10 min on 10% SDS, 10 min on denaturation solution (0.5 M NaOH, 1.5 M NaCl), twice for 5 min on neutralization solution (1.5 M NaCl, 0.5 M Tris-HCl (pH 7.4)), and 15 min on 2× SSC (3 M NaCl, 0.3 M sodium citrate (pH 7)). TBS washing (10 mM Tris-HCl, 150 mM NaCl (pH 7.4)) and gentle scraping the surface of the membrane with a folded Whatman paper removed excess cellular debris. TBS containing 3% BSA was used to block membranes for 1 h at RT. Overnight incubation in a moist chamber at 4°C on a rocking platform with 5 ml (per membrane) of blocking solution containing a 1:4,000 dilution of anti-SipC rabbit antibody provided by Virotech Diagnostics GmbH (reference number: VT110712) was used to stain the membranes. Washing with TBS-T (20 mM Tris-HCl, 500 mM NaCl, 0.05% Tween 20, 0.2% Triton X-100 (pH 7.5)) once and with TBS twice removed nonspecific binding. Membranes were then incubated with secondary antibodies (1:2,500 dilution of goat anti-rabbit IgG conjugated to HRP; Sigma; catalogue number A0545-1ML; in 5 ml blocking solution) at RT on a rocking platform for 2 to 4 h. Additional washes (3×) with TBS were performed before adding 5 ml of substrate per membrane: a 30 mg tablet of 4-chloro-1-naphthol (Sigma) dissolved in 10 ml of methanol, mixed with H_2_O_2_ (0.06% w/v) in 50 ml of TBS. Once the desired intensity was observed, the reaction was stopped with dH_2_O.

Clones without detectable SipC-specific signal were identified by comparing the colony blot assay data with the colony pattern on the original MacConkey agar plates. Desired isolates were matched to the original MacConkey plate, picked from those plates and inoculated in LB containing streptomycin. Isolates were then stored in 20% glycerol/LB at −80°C until whole-genome bacterial sequencing was performed.

It should be noted that this assay is well suited to identify clones with genetic defects in the genes required for *sipC* expression (which include *hilD*). However, as signal to noise ratios are in some cases quite small, there is a small chance for false-negatives that is clones which are in principle able to express SipC on the plate, but which fail to yield sufficient signal in the given assay. This might be the case in a subgroup of the 18% of all clones from the wild-type *S*. Typhimurium that did not express SipC, but which retained a functional *hilD* gene (see Fig 1E). In conclusion, the colony blot assay is useful to monitor within-host evolution of mutants with reduced virulence. However, competitive infection assays with isogenic strain pairs should be performed to confirm fitness effects.

### Whole-genome sequencing

Clones stored in 20% glycerol/LB at −80°C were regrown in LB with streptomycin. Genomic DNA was extracted using a QIAamp DNA Mini Kit (Qiagen). The Functional Genomics Centre Zurich or Novogene (Cambridge) performed Illumina MiSeq sequencing to generate 150 bp paired-end reads with at least 50× coverage across the genome. Analysis was performed using CLC Genomics Workbench versions 11, 12, and 20. Paired-end reads were mapped to the SL1344 chromosome reference (NCBI accession FQ312003.1). Basic variant detection was performed to detect variants that occurred in a minimum of 70% of reads and detect amino acid changes. S1–S6 Tables contain summaries of non-synonymous mutations in coding sequences that were absent in the ancestor strains (SL1344 and SL1344^*ssaV*^. S6 Table contains mutations detected in the ancestral strains.

### Gut microbiota community analysis using 16S rRNA sequencing

Fecal pellets were collected from mice and immediately stored at −20°C. Genomic DNA was extracted from the fecal pellet using the AllPrep DNA/RNA Mini Kit (Qiaqen) with a modified protocol for homogenization and disruption of the bacterial membranes. Approximately 600 ml of RLT buffer and two 3 mm metal beads were used for bead-beating at 25 Hz for 3 min using the TissueLyser (Qiagen). After a brief centrifugation step, supernatants containing the bacteria were transferred to a tube containing 0.9 mg of 0.1 mm zirconia beads (OPS Diagnostics) for homogenization (twice at 30 Hz for 3 min with 5 min incubation between each homogenization run). The samples then transferred to the DNA-binding columns following centrifugation at full speed for 3 min to pellet the cell debris. The supernatants were loaded onto the DNA-binding columns and DNA was eluted in 100 μl elution buffer (EB). Exceptionally for 4 samples (*S*.Tm; day 160; mouse 1–4), DNA was extracted from fecal samples resuspended in PBS due to technical reasons.

The concentration of genomic DNA was determined by Qubit measurements, and 5 ng of DNA was used as input to create a sequencing library using a [87] two-step PCR approach. Locus-specific degenerative primers 515f (5′-GTGCCAGCMGCCGCGGTAA-3′) and 806r (5′-GGACTACHVGGGTWTCTAAT-3′) were used to amplify the V4 region of the 16S rRNA gene. The first PCR ran under the following conditions, using the Q5 High-Fidelity DNA polymerase (BioConcept, New England BioLabs): Initial denaturation step at 98°C for 30 s, followed by denaturation for 10 s at 98°C, annealing for 30 s at 56°C, and extension for 30 s at 72°C. The steps after initial denaturation were repeated 15 times, followed by a final extension step at 72°C for 2 min, after which the temperature was held constant at 4°C. Following the first PCR step, PCR products were purified using 0.8 × reaction volumes of CleanNGS magnetic beads (LABGENE SCIENTIFIC SA) and eluted in 13 μl EB. Cleaned PCR products were used as input for the second PCR step with identical conditions as the first PCR step but running 20 instead of 15 cycles. During this step, unique dual-index barcodes were added to each sample to enable multiplexing. The quantity and quality of the final PCR products (approximately 450 bp) was assessed using a fragment analyzer (Advanced Analytical). The final library was pooled equimolarly depending on the concentration of the fragment analyzer peak that represented the target fragment. The final library was sequenced using paired-end read sequencing on the MiSeq platform at the Genome Engineering and Measurement Lab (GEML, Zürich). Lengths of the amplicons were 300 bp. A total of 34,412,752 sequencing reads from 246 samples (median = 64,486) served as input for the inference of ASVs using dada2 [88]. Primer sequences were removed using cutadapt [87] and only inserts that contained both primers and were as least 75 bases were kept for downstream analysis. Next, reads were quality filtered using the filterAndTrim function of the dada2 package (maxEE = 2, truncQ = 3, trimRight = (40, 40)). The learnErrors and dada functions were used to calculate sample inference using pool = pseudo as parameter. Reads were merged using the mergePairs function and bimeras were removed with the removeBimeraDenovo function (method = pooled). Remaining ASVs were then taxonomically annotated using the IDTAXA classifier [89] in combination with the Silva v138 database [90]. The final table (after excluding negative and positive controls) contained 1,081 ASVs. Individual library sizes ranged from 18,634 to 140,867, with a median of 45,669. We rarefied all samples to an even depth of 18,634 reads. Differentially enriched ASVs in different conditions are listed in S7–S10 Tables.

### Statistical analysis

For mouse experiments, nonparametric statistical testing was performed using GraphPad Prism 8 for Windows. Analysis of 16S amplicon sequencing was performed in R version 4.1.0 [91]. The Shannon index of diversity was computed as the main alpha-diversity metric and diversity differences between groups of samples were statistically tested with two-sample Wilcoxon rank sum tests. Permutational MANOVA based on Bray–Curtis dissimilarity after square-root transformation of abundances was used to infer differences between microbial communities of different groups of mice. Enrichment of individual ASVs was inferred using differential abundance testing using the DESeq2 package (version 1.28.1) with shrunken log2 fold changes using the apeglm method [92].

## Supporting information

S1 Data Excel spreadsheet containing, in separate sheets for each figure, the underlying numerical data and statistical analysis used for Figs 1B–1E, 2B, 2C, 3B–3E, 4B–4D, 5B–5D, 5F, 6A–6G, 7B–7D, S2A–S2C, S2E, S3A, S3C, S3D, S4A–S4C, S5A, S5C, S6B–S6K, S7A, S7B, S7D, S8A, S9A, S9B, S11B, S11C, S12B–S12D, S13A–S13H, S14A, S14B, S15B, S16B, S17A–S17D and S18B–S18E. (XLSX)

S1 Text Supplementary discussions A,B, and C to extend the arguments discussed in the main manuscript text and to give a more comprehensive summary of perspectives on the related topics. (DOCX)

S1 Fig Signals, benefits, and costs of the HilD-regulon expression. (A) The HilD regulon (center) has a complex architecture (for details, see [34,35]) and computes responses to a variety of environmental signals. Many of these signals are derived or controlled by the microbiota and the host. They provide environmental cues for controlled expression of *S*. Typhimurium virulence factors and physiological adaptations ensuring growth and survival such that the associated costs occur only at those moments of the infection cycle when the respective virulence factors are needed. The HilD regulon limits their costly expression to the gut lumen and shuts them off after mucosa invasion [36]. Moreover, the HilD regulon limits these costs of virulence expression to a subpopulation of the gut-luminal *S*. Typhimurium cells by ensuring that only a subset of the pathogen population is expressing *hilD* (bistable expression) [28,29]. Thus, in hosts with severely disrupted microbiota, the main function of the HilD regulon seems to reside in minimizing the costs associated with the triggering of gut inflammation by TTSS-1-, flagella-, and/or Sii-adhesin-dependent mucosa invasion. This host response promotes pathogen blooms in the gut lumen and thereby enhances transmission. The exact molecular nature of these costs is still being explored. In antibiotic pretreated mice, these costs entail reduced growth rates as observed ex vivo [37], death from innate host defenses encountered after tissue invasion [28,93], an altered sensitivity towards SCFA-mediated growth restriction [62,94], or a reduced energy level and an increased sensitivity of the regulon-expressing cells towards outer membrane disruption [95]. The sheer number of genes and phenotypes controlled by the HilD regulon [96] has been an obstacle in deciphering the cause of its expression costs. Our work presented here suggests that the gut microbiota are playing a critical role, by conditioning the gut milieu and thereby affect the relative costs and benefits of the HilD-regulon outputs. This explains why mice with intermediate CR tend to select for *S*. Typhimurium clones retaining an intact HilD-regulon. (B) HilD-regulon expression phenotypes of the key *Salmonella* strains used in this study. It should be noted that colonies derived from strains featuring an intact HilD regulon will express low levels of virulence factors when growing as colonies on plates. Therefore, these colonies will yield a positive western blot signal in the colony protein blot assay, as they secrete the TTSS-1 translocon and effector protein SipC [29,50].
(TIF)

S2 Fig Analysis and comparison of gut inflammation in streptomycin pretreated *S*.Tm- or *S*.Tm*-infected CON^E^ or CON^X^ mice. Streptomycin pretreated C57BL/6 (CON^E^) were infected with either wild-type *S*.Typhimurium (pink; *n* = 15, *n* = 9 euthanized at day 1 p.i. and *n* = 6 at day 4 p.i.; 5 independent experiments) or *S*.Tm* (pink; *n* = 16, *n* = 9 euthanized at day 1 p.i. and *n* = 7 at day 4 p.i.; 5 independent experiments) or *S*.Tm^Avir^ (purple; *n* = 13, *n* = 7 euthanized at day 1 p.i., *n* = 6 at day 4 p.i.; 4 independent experiments) with total CFU of 5 × 10^7^. Of note, all the inoculums contained 2.5 × 10^6^ CFUs (1:20) of *S*.Tm^Avir^ (*tsr*- vs. *tsr*+) as a reporter for the type of inflammation [97]. (A) Fecal populations of *S*.Tm (*n* = 15 mice analyzed), *S*.Tm* (*n* = 16 mice analyzed), and *S*.Tm^Avir^ (*n* = 9 mice analyzed) were determined by selective plating at day 1 or day 4 p.i. (B) mLN counts of *S*.Tm^WT^ (*n* = 15 mice analyzed), *S*.Tm* (*n* = 16 mice analyzed), and *S*.Tm^Avir^ (*n* = 9 mice analyzed) at day 1 or day 4 p.i. (C) Spleen counts of *S*.Tm (*n* = 6 mice analyzed) vs. *S*.Tm* (*n* = 6 mice analyzed) at day 4 p.i. (D) Representative images of hematoxylin and eosin (HE) stained cecal tissue sections from mice infected with *S*.Tm or *S*.Tm* at day 1 or day 4 p.i. Lu. = Lumen. S.E. = Submucosal edema. Black arrows indicate collapse of upper mucosa. Scale bar = 100 μm. (E) Histopathology scoring of the sections from panel D (*n* = 6 at day 1 and *n* = 6 for day 4 p.i. for *S*.Tm; *n* = 6 at day 1 and *n* = 5 for day 4 p.i. for *S*.Tm*). (F) Representative images of HE-stained cecum tissue sections (from 3 different mice) from streptomycin pretreated 129/SvEv (CON^X^) mice that were infected with either wild-type *S*.Typhimurium (*S*.Tm:*S*.Tm^*hilD*^; 1,000:1 ratio) or *S*.Tm*(*S*.Tm*:*S*.Tm*^hilD^; 1,000:1 ratio) for 4 days; Lu. = Lumen. S.E. = Submucosal edema. Black arrows indicate collapse of upper mucosa. Scale bar = 100 μm. Dotted lines indicate the detection limit. Lines indicate the median. Two-tailed Mann–Whitney U tests were used to compare *S*.Tm to *S*.Tm* (*p* > 0.05 not significant (ns), *p* < 0.05 (*), *p* < 0.01 (**), *p* < 0.001 (***), *p* < 0.0001 (****)). Source data can be found in S1 Data file. Interpretation: As expected in this model, both *S*.Tm and *S*.Tm* colonized these mice to the carrying capacity for 4 days, and *S*.Tm achieved higher systemic organs loads than *S*.Tm*. In line with previous work in C57BL/6 mice, *S*.Tm elicited a more pronounced colitis than *S*.Tm*, while *S*.Tm*-infected mice showed signs of epithelial recovery from mucosal inflammation at day 4 p.i., marked by crypt hyperplasia, the return of mucus containing goblet cells, and reduced epithelial cell expulsion.
(TIF)

S3 Fig Additional analysis of gut inflammation and *Salmonella* pathogen loads in the systemic organs of CON^X^ mice infected with wild-type *S*. Typhimurium or *S*.Tm* (animals from Fig 1A–1D). (A) RTqPCR analysis of the pro-inflammatory gene expression in the cecum tissue of mice infected for 70 days with wild-type *S*. Typhimurium (pink; *n* = 7 mice analyzed) or *S*.Tm* (black; *n* = 7 mice analyzed). The results are plotted as fold-induction over the average of mice infected with *S*.Tm*. (B) Representative images of cecal sections at day 70 p.i. Nuclei (blue; DAPI), neutrophils (red; Ly6B.2), and actin (white; Phalloidin) are stained. Lu. = Lumen. White arrows point to neutrophils. Scale bar = 50 μm. HPF; high field of view. (C) Quantification of neutrophils in cecal sections. Each data point is the average of 5 fields of view (FOV) per mouse (*n* = 7 mice per group). Lines indicate the median. (D) *Salmonella* organ loads of mice euthanized on day 70 p.i. (*n* = 7 for both groups) with wild-type *S*. Typhimurium (*n* = 7) or *S*.Tm* (*n* = 7). Lines indicate the median. mLN = mesenteric lymph node. Blood is reported as CFU per ml. Lines indicate the median. Two-tailed Mann–Whitney U tests were used to compare the data between the groups infected with wild-type *S*. Typhimurium and *S*.Tm* (*p* ≥ 0.05 not significant (ns), *p* < 0.05 (*), *p* < 0.01 (**), *p* < 0.001 (***)). These data show that innate and adaptive immunity are controlling the infection by both strains about equally well by day 70 p.i. Source data can be found in S1 Data file.
(TIF)

S4 Fig Further analysis of the colony protein blot data from Fig 1D. (A) Fraction of the fecal *Salmonella* population which yielded colonies where SipC was detected. (B) Estimate of the total size of the *Salmonella* population which yielded colonies where no SipC was detected. (C) Estimate of the total size of the *Salmonella* population which yielded colonies where SipC was detected. (A–C) The black dotted line indicates the conservative detection limit. Colored lines connect medians. Transparent pink dots (at >20 days p.i.) indicate that the calculation of the absolute sizes of the respective *Salmonella* populations is not very reliable as the majority of mice have >95% clones where no SipC was detected. Two-tailed Mann–Whitney U tests were used to compare wild-type *S*.Typhimurium to *S*.Tm* infected groups (*p* ≥ 0.05 not significant (ns), *p* < 0.01 (**), *p* < 0.001 (***), *p* < 0.0001 (****)). Interpretation: During the first 20 days, *S*.Tm*-infected mice shed more pathogen cells capable of SipC expression in their feces than the mice infected with wild-type *S*. Typhimurium (S4C Fig; 9.5 × 10^8^ CFU per g feces per day vs. 6.5 × 10^8^ CFU per g feces per day; average over 20 days). After 20 days of infection with wild-type *S*. Typhimurium, clones capable of expressing SipC were rarely detected in the feces, which prevented a reasonable estimation of the total fecal pathogen loads that remained capable of expressing SipC between days 20–70 p.i. In contrast, *S*.Tm*-infected mice shed low but confidently detectable loads of clones that remained capable of expressing SipC (>10^4^ CFU/g feces) until day 70 p.i. (S4C Fig). Thus, over the course of 70 days, *S*.Tm*-infected mice shed higher loads of clones capable of SipC expression (approximately >1.5-fold) and lower loads of clones incapable of SipC expression (approximately 10^4^-fold less) in their feces compared to wild-type *S*. Typhimurium-infected mice. Source data can be found in S1 Data file. Of note, the comparison of the total population sizes that yield colonies with detectable SipC expression is not informative after day 20 p.i., since in mice infected with wild-type *S*. Typhimurium most fecal samples contained >95% clones where SipC was not detected (of the plated clones). Due to the limited number of colonies per plate, the absolute number cells which would yield colonies where SipC could be detected could not be measured with our assay. More accurate analysis of the population sizes could be obtained in C.I. experiments performed later on in this study, where detection is based on selective plating (e.g., Fig 4).
(TIF)

S5 Fig Reduced virulence of fecal pathogen populations sampled after 70 days of infection with wild-type *S*. **Typhimurium (from mice in Fig 1B–1D).** (A) Experimental scheme. Fecal suspensions from mice in Fig 1A–1D (CON^X^ 70 days after infection with wild-type *S*. Typhimurium) were transferred into streptomycin pretreated CON^X^ mice (*n* = 6 mice; gray; 100 μl of 1 ml fecal suspension in PBS, by gavage). This provided us with a means to assess virulence after transmission. In contrast to the transmission by co-housing, the gavage of fecal suspensions allowed a more precise timing and dosing of the infection. The infection dynamics were compared to the original infection with wild-type *S*. Typhimurium in Fig 1A–1D at days 1–3 p.i. (*n* = 19 mice; pink). (B) Fecal *Salmonella* populations were enumerated by selective plating. (C) Lipocalin-2 concentrations in the feces, as determined by ELISA. Colored lines connect medians between time points. Dotted lines indicate the detection limits. Two-tailed Mann–Whitney U tests were used to compare the 2 groups at each time points (*p* ≥ 0.05 not significant (ns), *p* < 0.05 (*), *p* < 0.001 (***)). Source data can be found in S1 Data file. It should be noted that the experimental group developed gut Inflammation much later (days 3–4 p.i.), than the mice infected with the original wild-type *S*. Typhimurium strain (day 1–2 p.i.). This is consistent with a loss of TTSS-1 inflicted gut inflammation by the evolved *Salmonella* population. We hypothesize that the residual capacity to elicit enteropathy by this evolved population is attributable to TTSS-2-mediated pathogen growth in the gut tissue, which can elicit a delayed form of enteropathy in streptomycin pretreated mice (that is termed “alternative pathway”; [22]).
(TIF)

S6 Fig Infection kinetics of wild-type *S*. **Typhimurium and *S*.Tm**^***hilD***^ **in mouse models conferring different degrees of CR (extends evidence related to Fig 2).** (A) Experimental scheme. Germ-free mice confer no CR, can be infected via the orogastric route (this does not require any type of pretreatment) and wild-type *S*. Typhimurium will bloom up to carrying capacity (10^9^–10^10^ CFU/g feces) and to elicit pronounced enteropathy within 12 h of infection [4,44]. LCM mice harbor a microbiota consortium composed of 8 strains and confer an intermediate level of CR. Upon orogastric infection, wild-type *S*. Typhimurium takes 3–4 days to grow to carrying capacity and 2–4 days to elicit pronounced enteropathy [4,46,47]. OligoMM^12^ mice harbor a defined microbiota composed of 12 representative microbiota strains [57]. They confer an intermediate level of CR. Upon orogastric infection, wild-type *S*. Typhimurium takes 3–4 days to grow to carrying capacity and to elicit pronounced enteropathy [4,25,57]. The complex SPF microbiota of CON^E^ mice confers strong CR and prevents in most animals gut luminal growth of wild-type *S*. Typhimurium and of attenuated *Salmonella* mutants, alike. Thus, after orogastric infection, the *Salmonella* loads in the feces remain low in most CON^E^ mice (typically <10^6^ CFU/g feces) and no enteropathy develops within the first 5 days of infection (similar to CON^X^ mice). For comparison, we also re-plot the data for CON^X^ (from Fig 2) that were shifted from their normal plant based diet to a high-fat diet for the day before the infection. The 24 h exposure to high-fat diet reduces the degree of CR in the CON^E^ mice so that wild-type *S*. Typhimurium can grow up to densities of ≈10^8^ CFU/g gut-luminal content and elicits enteropathy by day 3–4 in most animals [4,25]. (B–K) Groups of the indicated mice were infected either with wild-type *S*. Typhimurium (5 × 10^7^ CFU, by gavage; gray circles; *n* = 6 for GF, *n* = 4 for LCM, and *n* = 6 for Oligo-MM^12^; *n* = 10 for CON^E^; *n* = 13 for transient diet shift) or with an isogenic *hilD* mutant (*S*.Tm^*hilD*^; 5 × 10^7^ CFU, by gavage; red circles; *n* = 7 for GF, *n* = 5 for LCM, and *n* = 6 for Oligo-MM^12^; *n* = 10 for CON^E^; *n* = 12 for transient diet shift). The models are represented in an order of lowest to highest CR (left to right). (B–F) Fecal lipocalin-2 was measured by an ELISA as a measure for *Salmonella*-induced enteropathy in these mice. (G–K) Fecal *Salmonella* loads were determined by selective plating. Dotted lines indicate the detection limit. Colored lines connect the medians. Two-tailed Mann–Whitney U tests were used to compare wild-type *S*. Typhimurium to *S*.Tm^*hilD*^-infected groups at each time point (*p* ≥ 0.05 not significant (ns), *p* < 0.05 (*), *p* < 0.01 (**), *p* < 0.001 (***), *p* < 0.0001 (****)). Source data can be found in S1 Data file. Interpretation: In mice with strong CR (such as unperturbed CON^E^ mice), neither the wild-type nor the mutant pathogen can grow in the gut lumen or elicit enteropathy, as indicated by low fecal pathogen loads and lipocalin-2 concentrations below 10^2^ ng/g feces. In mice with intermediate levels of CR, the wild-type *S*. Typhimurium can grow up to higher gut-luminal densities within 2–4 days (as indicated by pathogen loads of >10^8^ CFU/g in the feces) and elicit more pronounced enteropathy than *S*.Tm^*hilD*^. This supports that *S*.Tm^*hilD*^ has a reduced virulence in the gut-luminal environment associated with intermediate levels of CR. In germ-free mice, both, wild-type *S*. Typhimurium and *S*.Tm^*hilD*^ can grow up to high gut-luminal densities (as measured by pathogen loads of ≈10^10^ CFU/g in the feces). This is explained by the lack of CR which obliviates the need for inflammation-mediated microbiota suppression. Nevertheless, wild-type *S*. Typhimurium elicits more pronounced enteropathy than *S*.Tm^*hilD*^, as indicated by the reduced fecal lipocalin-2 concentrations. These data confirm that *S*.Tm^*hilD*^ has a reduced virulence compared to the isogenic wild-type strain.
(TIF)

S7 Fig Pathogen loads in the organs and quantitative analysis of cecum tissue inflammation for mice at the end of the microbiota transfer experiment shown in Fig 3A–3D. Mice had been infected with wild-type *S*. Typhimurium (as described in Fig 3) and co-housed (red circles with gray filling; *n* = 6 mice per group) or not (red filled circles; *n* = 6 mice per group) with an unperturbed CON^X^ donor mouse from day 70 p.i. on. (A) Organ loads at day 160 p.i. (*n* = 6 mice per group). Lines indicate the median. mLN = mesenteric lymph node. Blood is reported as CFU per ml. (B) Pro-inflammatory gene expression analysis at day 160 p.i. (*n* = 6 mice per group). The results are plotted as fold induction from cecal tissue of mice w/o co-housing over the average of mice co-housed with a CON^X^ donor mouse. (C) Representative immunofluorescence images of cecal tissue sections from mice of both groups at day 160 p.i. Nuclei (blue; DAPI), neutrophils (red; Ly6B.2), and actin (white; Phalloidin) are stained. Lu. = Lumen. White arrows indicate neutrophil recruitment to the cecal epithelium. Scale bar = 50 μm. (D) Quantification of neutrophils in cecal tissue sections as shown in panel C (*n* = 6 mice per group). (A–D) Two-tailed Mann–Whitney U tests were used to compare *S*.Tm to *S*.Tm + CON^X^ (*p* ≥ 0.05 not significant (ns), *p* < 0.05 (*), *p* < 0.01 (**)). Source data can be found in S1 Data file.
(TIF)

S8 Fig Re-sequencing of clones isolated from wild-type *S*. **Typhimurium infected mice at day 160 p.i. (relates to Fig 3A–3D).** Genome re-sequencing was performed on clones isolated from mice in Fig 2A–2E. A complete overview of the non-synonymous mutations is summarized in S3 Table. Genes mutated clones without detectable SipC expression were recovered from the control mice infected with wild-type *S*. Typhimurium 160 days without co-housing. Genes mutated only in *mutS* mutant mutator strains are excluded from this graph. Only non-synonymous mutations are shown. Genes are sorted according to the frequency of mutations found in the respective gene (*n* = 20 clones total; dotted line indicates the percentage that corresponds to a mutation in only 1 clone). *hilD* mutations were detected in 87% of all analyzed clones and the frequency of *hilD* mutations was even higher (and additional mutations accumulated) compared to the clones isolated at days 50–70 p.i. (S2 Table). Source data can be found in S1 Data file.
(TIF)

S9 Fig Additional analyses of the microbiota composition in the mice from Fig 3. (A) Principal coordinate analysis based on Bray–Curtis dissimilarities between samples (after the square-root transformation of abundances). Data points represent individual mice at day 160 p.i. PERMANOVA R^2^ = 0.284 and *p* = 0.017 for day 120 p.i. and R^2^ = 0.4034 and *p* = 0.009. (B) 16S community sequencing analysis of fecal samples at day 120 and day 160 p.i. from control or co-housed mice (*n* = 6 fecal microbiotas were analyzed for each group). Source data can be found in S1 Data file. The bar plot shows the relative abundance of the dominant phyla. The four most abundant phyla are shown, with the rest of the community shown as “other.”
(TIF)

S10 Fig Cecum tissue pathology of the mice from Fig 4. (A) Representative images of hematoxylin and eosin (HE) stained cecal tissue sections from mice infected with *S*.Tm*:*S*.Tm*^hilD^ for 40 days, or *S*.Tm:*S*.Tm^*hilD*^ for 80 days, or infected with *S*.Tm:*S*.Tm^*hilD*^ for 40 days and co-housed with a recovered mouse for another 40 days Lu. = Lumen. S.E. = Submucosal edema. Black arrows indicate submucosal edema. Scale bar = 100 μm.
(TIF)

S11 Fig *hilD* mutants are selected for by the inflammation elicited by wild-type *S*. **Typhimurium and this happens in invasive and non-invasive strain backgrounds.** Streptomycin pretreated C57BL/6 mice harboring a complex specified pathogen free microbiota (termed CON^E^ mice in this work) were infected with the following mixtures of *Salmonella* strains (5 × 10^7^ CFU, by gavage): (1) *S*.Tm^Avir^:*S*.Tm^AvirhilD^: ***S*.Tm**^**Avir**^ (1:1:10.000; *n* = 4 mice per group) (Avirulent conditioned); (2) *S*.Tm:*S*.Tm^hilD^: ***S*.Tm**^**Avir**^ (1:1:10.000; *n* = 3 mice per group) (Avirulent conditioned); (3) *S*.Tm^Avir^:*S*.Tm^AvirhilD^: ***S*.Tm** (1:1:10.000; *n* = 3 mice per group) (wild-type conditioned); (4) *S*.Tm:*S*.Tm^hilD^: ***S*.Tm** (1:1:10.000; *n* = 3 mice per group) (wild-type conditioned) for 4 days. (A) Experimental scheme. (B) The competitive index (C.I.), as calculated by the ratio of the isogenic *hilD*-deficient vs. *hilD*-proficient strain pairs. The dotted line indicates a C.I. of 1. (C) Lipocalin-2 ELISA was performed on feces to compare the level of gut inflammation between the different groups. The dotted line indicates the detection limit. The color code indicates the degree of gut inflammation ([Lcn2]>200–1,000 ng/g feces; mild gut inflammation, [Lcn2] = 1,000–1,000.000 ng/g feces, pronounced gut inflammation). Source data can be found in S1 Data file.
(TIF)

S12 Fig Serial transmission experiment in streptomycin pretreated CON^E^ vs. **untreated OligoMM**^**12**^. (A) Experimental scheme. Streptomycin pretreated CON^E^ mice (strongly disrupted CR; *n* = 5 mice; 2 independent experiments; pink symbols) and Oligo-MM^12^ mice (intermediate CR; *n* = 5 mice; 2 independent experiments; blue symbols) were infected with the same wild-type *S*. Typhimurium strain (5 × 10^7^ CFU, by gavage). Fecal pellets were collected every third day, resuspended and used to infect the next group of mice (2 mice per 1 infected mouse pellet) by oral gavage at the day of collection. Mice from the last (fourth) transmission were infected for 4 days (*n* = 10 for each group). (B) Fecal *Salmonella* loads as determined using MacConkey plates with selective antibiotics. (C) Lipocalin-2 concentrations in the feces, as determined by ELISA. (D) Fraction of clones without detectable SipC, as determined by the colony protein blot assay. Two-tailed Mann–Whitney U tests were used to compare CON^E^ + streptomycin to Oligo-MM^12^ at each time point (*p* ≥ 0.05 not significant (ns), *p* < 0.05 (*), *p* < 0.01 (**), *p* < 0.001 (***), *p* < 0.0001 (****)). Source data can be found in S1 Data file. Interpretation: In line with previous work, wild-type *S*. Typhimurium elicited enteropathy (as measured by lipocalin-2 ELISA) in both groups of mice. However, the disease had faster kinetics and was more pronounced in the streptomycin pretreated CON^E^ mice than in the Oligo-MM^12^ mice develop moderate colitis in the first 4 days of the infection [85]. As we transmitted fecal suspensions into naive mice every third day (for 4 times), we ensured that OligoMM^12^ would not develop proceed to pronounced colitis (which would be expected to occur at day 4 p.i.). This design had 2 important advantages: We could achieve more vs. less pronounced colitis over the entire course of the experiment while using the exact same wild-type *S*. Typhimurium strain. And we could avoid life-threatening degrees of systemic pathogen spread, which would occur beyond days 5–6 after high-level gut colonization in streptomycin pretreated C57BL/6 mice. At day 3 of the first round of infection, mice from both groups shed *S*.Tm populations consisting of 100% clones with detectable SipC expression. In contrast, by the end of the fourth transmission, we detected clones without detectable SipC expression in all animals of the antibiotic pretreated CON^E^ mice, while no such clones were detected in the feces of the Oligo-MM^12^ mice (median = 20% vs. 0% clones without SipC expression). This experiment further substantiated that a high degree of gut inflammation is correlated with the selection for *S*. Typhimurium mutants with reduced virulence.
(TIF)

S13 Fig Additional analysis of microbiota diversity and community composition in mice from Fig 1B–1D (relates to Fig 6). 16S community sequencing analysis of fecal samples at day 10 and day 70 p.i. from mice in Fig 1B–1D. (A–C) Principal coordinate analysis based on Bray–Curtis dissimilarities between samples (after the square-root transformation of abundances). Data points represent individual mice, and a colored border defines the grouping of data points within each sample group. PERMANOVA R2 = 0.067 and *p* = 0.589 for day 0 p.i., R^2^ = 0.397 and *p* = 0.0045 for day 10 p.i., and R^2^ = 0.660 and *p* = 0.002 for day 70 p.i. (D) Within-sample diversity as measured by the number of observed species. (pink circles: wild-type *S*. Typhimurium *S*.Tm; black circles: *S*.Tm*). (E–G) Relative order abundances compared at day 10 and 70 p.i. Fecal microbiota from unpertubed CON^X^ mice served as reference point for the normal CON^X^ microbiota. (E) Bacteroidales of the phylum Bacteroidetes. (F) Enterobacterales of the phylum Proteobacteria. (G) Coriobacterales of the phylum Actinobacteria. (H) Relative abundances of the most abundant genera belonging to the order Erysipelotrichales. Genera were compared at 70 p.i. between mice infected with wild-type *S*. Typhimurium vs. *S*.Tm*. Fecal microbiota from unpertubed CON^X^ mice served as reference point for the normal CON^X^ microbiota (gray circles). Dotted line indicates the detection limit. Lines indicate the median. Two-tailed Mann–Whitney U tests were used to compare wild-type *S*. Typhimurium to *S*.Tm*-infection or *S*.Tm*-infections to untreated CON^X^ (*p* ≥ 0.05 not significant (ns), *p* < 0.05 (*), *p* < 0.01 (**), *p* < 0.001 (***), *p* < 0.0001 (****)). Source data can be found in S1 Data file. Interpretation: A striking dominance of genus *Turicibacter* in post-colitis gut was observed in both experimental groups in dependent of the level of gut inflammation, suggesting a superior adaptation of these bacteria to the inflamed gut. Besides, genus *Faecalibaculum* was differentially enriched only in the post-colitis gut of the *S*.Tm*-infected mice. Which could be interpreted as that these bacteria can regrow back only in the gut conditions with resolving inflammation.
(TIF)

S14 Fig Analysis of SCFAs and other gut luminal metabolites in the cecum content of untreated CON^X^, OligoMM^12^, germ-free, and *S*.Tm-infected CON^X^ mice. This analysis focused on the cecum content, as this is the site where most *Salmonella* and microbiota growth occurs. The cecum content from untreated CON^X^ (*n* = 5 animals), OligoMM^12^ (*n* = 5 animals), and germ-free mice (*n* = 5 animals) was collected. Streptomycin pretreated CON^X^ mice (*n* = 8 animals) were infected with a 10^6^:1 mixture of wild-type *S*. Typhimurium and *S*.Tm^*hilD*^ and the cecal content was collected at day 40 p.i. The bacteria were removed by centrifugation and we collected the supernatants to quantify the metabolites accessible in the liquid content of the cecum lumen. Metabolite concentrations were determined by mass spectrometry with respect to internal standards for the SCFAs acetate, propionate, and butyrate or with respect to ^13^C labeled succinate for the other metabolites, such as lactate, malate, pyruvate, 2-oxoglutarate, succinate, and fumarate. (A) Concentrations of acetate, propionate, and butyrate. Dotted line indicates the limit of quantification (LOQ), values on the dotted line are below LOQ. (B) Relative abundance of lactate, malate, pyruvate, 2-Oxoglutarate, succinate, and fumarate as measured by the log2 transformed area ratio normalized to labeled succinate. Values on the X-axis are below the detection limit. Two-tailed Mann–Whitney U tests were used to compare untreated CON^X^ mice to CON^X^ mice at day 40 p.i. with wild-type *S*. Typhimurium (*p* ≥ 0.05 not significant (ns), *p* < 0.05 (*), *p* < 0.01 (**), *p* < 0.001 (***), *p* < 0.0001 (****)). Source data can be found in S1 Data file. Interpretation: The drop in the SCFA concentrations confirms that the wild-type *S*. Typhimurium infection disrupts the normal physiology of the CON^X^ microbiota quite strongly and over long periods of time, as indicated by the day 40 p.i. data.
(TIF)

S15 Fig Test of candidate microbiota strains and microbiota metabolites to assess their effect on the selection against *S*.Tm^*hilD*^. (A) Experimental scheme. (B) Streptomycin pretreated CON^X^ mice (*n* = 35) were infected with a mixture of *S*.Tm* and isogenic *hilD* mutant (*S*.Tm*:*S*.Tm*^hilD^; 10^3^:1 ratio) at day 0 and we analyzed pathogen gut colonization for 4 days. The first group remained in hygienic isolation (control; black circles; *n* = 14). The second and third group also remained under hygienic isolation and received drinking water supplemented with 25 mM Na-propionate or 50 mM Na-butyrate. The remaining groups were inoculated with glycerol stock of overnight cultures of the indicated strains on days 0, 1, 3 p.i. (ca. 5 × 10^5^ to 5 × 10^7^CFU, by gavage; *n* = 3 (except for DSM17677, *n* = 4)). The coloring indicates the tested microbiota strain. The normalized C.I. is plotted as the ratio of the isogenic *hilD*-deficient vs. *hilD*-proficient strain pairs, determined by selective plating. Source data can be found in S1 Data file. Interpretation: These results suggest that the selection against the rise of *hilD* mutants is a result of a combined effect of diverse microbiota members or associated metabolites.
(TIF)

S16 Fig Competitive infection experiment testing the effect of a mixture of defined microbiota strains on the selection for *hilD* mutants. (A) Experimental scheme. Germ-free mice were taken from the end of the experiment shown in Fig 4. At this point, these germ-free mice had been infected for 40 days with a 100:1 mixture of *S*.Tm* and *S*.Tm* ^*hilD*^ (5 × 10^7^ CFU, by gavage; *n* = 5). On days 40, 45, and 50 after infection, the mice were inoculated, with glycerol stocks of o/n cultures from strains of OligoMM^12^ mice or from DSM collection (Cocktail A: KB18-YL32-YL31-YL58-DSM 103405; using an established protocol [57]). These microbiota strains were chosen to represent microbiota members that regrew by day 70 of infection with *S*.Tm* and which may therefore contribute to displacing mutant-dominated pathogen populations from the gut lumen. (B) Competitive index (C.I.) of *S*.Tm* ^*hilD*^ vs. *S*.Tm*, as determined using MacConkey plates with selective antibiotics. The C.I. is normalized to the inoculum. The dotted line indicates a C.I. of 1. The data for days 0–40 is re-plotted from Fig 4. Source data can be found in S1 Data file. Interpretation: The inoculation with the microbiota mixture had no detectable effect on the selection for or against *S*.Tm* ^*hilD*^ (compare days 20–40 to day 40–55 data). The reasons for this remain unclear. We speculate that this might be linked to our failure to identify the correct competitive microbiota strains, as our procedure to choose strains relied on 16S sequence information that does not provide any information on strain-specific traits that might be important for conditioning the gut-luminal milieu.
(TIF)

S17 Fig Additional analysis of fecal samples from the transient high-fat diet shift experiment (Fig 7). We re-analyzed the data from the experiment shown in Fig 7B–7D by splitting the mice into 2 subgroups based on the pathogen loads in the feces at day 10 p.i. High shedders (black symbols) had >10^7^ CFU/g feces; low shedders (gray symbols) had <10^7^ CFU/g feces at day 10 p.i. (A) Total *Salmonella* loads detected in the feces of high- and low shedders by selective plating. Dotted line indicates the detection limit. Black lines connect the medians. (B) Pathogen loads in the mLN and the spleen, as determined by plating. (C) The normalized competitive index (C.I.) high- and low shedders was determined by selective plating and is shown for wild-type *S*. Typhimurium versus *S*.Tm^*hilD*^. The dotted line indicates a C.I. of 1. (D) An ELISA for fecal lipocalin-2 was used to compare gut inflammation between the 2 groups. Two-tailed Mann–Whitney U tests were used for statistical analysis (*p* ≥ 0.05 not significant (ns), *p* < 0.05 (*), *p* < 0.01 (**), *p* < 0.001 (***), *p* < 0.0001 (****)). Source data can be found in S1 Data file. Interpretation: This model nicely summarizes the trade-off between induction of gut inflammation and reduced virulence. While higher inflammation supports increased number of shedding pathogen cells (i.e., high shedders), this comes with the problem of losing virulence (increased *hilD* mutant accumulation). Thus, neither too high nor too low is optimal for *S*.Tm gut colonization.
(TIF)

S18 Fig Competitive infection of *S*.Tm* and *S*.Tm*^hilD^ in LCM mice. (A) Experimental scheme. (B–E) LCM mice were infected with a 100:1 mixture of *S*.Tm* and the isogenic *hilD* mutant *S*.Tm* ^*hilD*^ (5 × 10^7^ CFU, by gavage; *n* = 7 mice) for 40 days. (B) Total fecal *Salmonella* loads, as determined using MacConkey plates with selective antibiotics. (C) Fecal *S*.Tm* and *S*.Tm* ^*hilD*^ loads, as determined using MacConkey plates with selective antibiotics. (D) Competitive index (C.I.) of *S*.Tm* ^*hilD*^ vs. *S*.Tm*, as determined using MacConkey plates with selective antibiotics. The C.I. is normalized to the inoculum. The dotted line indicates a C.I. of 1. (E) Lipocalin-2 ELISA as performed on the feces. We analyzed a minimum *n* = 3 fecal pellets per time point. Dotted lines indicate the detection limit. Lines connect the median values at the days of analysis. Source data can be found in S1 Data file. Interpretation: In the LCM mice, we observed a selection for the *hilD*-deficient strain during the first 10 days of the infection. This is likely attributable to the inflamed gut-luminal milieu, which selected for the *hilD* mutant. Also, the inflammation has likely disturbed the LCM microbiota such that it cannot establish conditions selecting against the *hilD* mutant during these 10 days. The gut inflammation was apparently resolved by days 20–40, as indicated by the lipocalin-e ELISA data. In this period of the experiment, the selective advantage of the *hilD*-deficient strain was reversed. We hypothesize that this is attributable to the re-establishment of the LCM microbiota and that this accounts for the 10,000-fold selection of the *hilD*-proficient over the *hilD*-deficient strain (C.I. = 10^2^ at day 10; versus C.I. = 10^−2^ at day 30).
(TIF)

S1 Table Summary of mutations detected in evolved clones isolated from mice infected with wild-type *S*. **Typhimurium at day 10–13 p.i.** For all clones (*n* = 16; 12 where SipC was detected, and 4 without detectable SipC expression; taken from mice in Fig 1A–1D), the SipC phenotype from our colony protein blot assay [29,50] (as a proxy for TTSS-1 expression) is indicated by a symbol and by the color-code of the column; + and the blue color code indicate positive detection of a SipC signal; +/- and the yellow color code indicate intermediate SipC expression (for the purposes of this study, we considered these SipC expressing clones);—and the red color code indicate the lack of detectable SipC expression. The table shows non-synonymous mutations that are absent from the ancestral strain (S6 Table). The position in the SL1344 reference chromosome (NCBI accession FQ312003.1) is indicated along with the allele change. If blank, the region of the genome contains no mutations. If more than 1 mutation is observed in that gene, we simply show the number of variants present in that gene. Percentages indicate the fraction of isolates, which show a particular mutation. Mutations that occur only in clones with a *mutS* mutation (mutator strains) are listed in a separate sub-table labeled “Mutator-specific mutations.”
(XLSX)

S2 Table Summary of mutations detected in evolved clones isolated from mice infected with wild-type *S*. **Typhimurium at day 47–70 p.i.** For all clones (*n* = 22; 11 where SipC was detected, and 11 without detectable SipC expression; taken from mice in Fig 1A–1D), the SipC phenotype from our colony protein blot assay [29,50] (as a proxy for TTSS-1 expression) is indicated by a symbol and by the color-code of the column; + and the blue color code indicate positive detection of a SipC signal; +/- and the yellow color code indicate intermediate SipC expression (for the purposes of this study, we considered these SipC expressing clones);—and the red color code indicate the lack of detectable SipC expression. The table shows non-synonymous mutations that are absent from the ancestral strain (S6 Table). The position in the SL1344 reference chromosome (NCBI accession FQ312003.1) is indicated along with the allele change. If blank, the region of the genome contains no mutations. If more than 1 mutation is observed in that gene, we simply show the number of variants present in that gene. Percentages indicate the fraction of isolates, which show a particular mutation. Mutations that occur only in clones with a *mutS* mutation (mutator strains) are listed in a separate sub-table labeled “Mutator-specific mutations.”
(XLSX)

S3 Table Summary of mutations detected in evolved clones isolated from mice infected with wild-type *S*. **Typhimurium at day 157–160 p.i.** For all clones (*n* = 20; 5 where SipC was detected, and 15 without detectable SipC expression; taken from mice in Fig 3B–3D), the SipC phenotype from our colony protein blot assay [29,50] (as a proxy for TTSS-1 expression) is indicated by a symbol and by the color-code of the column; + and the blue color code indicate positive detection of a SipC signal; +/- and the yellow color code indicate intermediate SipC expression (for the purposes of this study, we considered these SipC expressing clones);—and the red color code indicate the lack of detectable SipC expression. The table shows non-synonymous mutations that are absent from the ancestral strain (S6 Table). The position in the SL1344 reference chromosome (NCBI accession FQ312003.1) is indicated along with the allele change. If blank, the region of the genome contains no mutations. If more than 1 mutation is observed in that gene, we simply show the number of variants present in that gene. Percentages indicate the fraction of isolates, which show a particular mutation. Mutations that occur only in clones with a *mutS* mutation (mutator strains) are listed in a separate sub-table labeled “Mutator-specific mutations.”
(XLSX)

S4 Table Summary of mutations detected in evolved clones isolated from mice infected with *S*.Tm* at day 10–12 p.i. For all clones (*n* = 21; 14 where SipC was detected, and 7 without detectable SipC expression; taken from mice in Fig 1A–1D), the SipC phenotype from our colony protein blot assay [29,50] (as a proxy for TTSS-1 expression) is indicated by a symbol and by the color-code of the column; + and the blue color code indicate positive detection of a SipC signal; +/- and the yellow color code indicate intermediate SipC expression (for the purposes of this study, we considered these SipC expressing clones);—and the red color code indicate the lack of detectable SipC expression. The table shows non-synonymous mutations that are absent from the ancestral strain (S6 Table). The position in the SL1344 reference chromosome (NCBI accession FQ312003.1) is indicated along with the allele change. If blank, the region of the genome contains no mutations. Percentages indicate the fraction of isolates, which show a particular mutation.
(XLSX)

S5 Table Summary of mutations detected in evolved clones isolated from mice infected with *S*.Tm* at day 47–70 p.i. For all clones (*n* = 12; 7 where SipC was detected, and 5 without detectable SipC expression; taken from mice in Fig 1A–1D), the SipC phenotype from our colony protein blot assay [29,50] (as a proxy for TTSS-1 expression) is indicated by a symbol and by the color-code of the column; + and the blue color code indicate positive detection of a SipC signal; +/- and the yellow color code indicate intermediate SipC expression (for the purposes of this study, we considered these SipC expressing clones);—and the red color code indicate the lack of detectable SipC expression. The table shows non-synonymous mutations that are absent from the ancestral strain (S6 Tables). The position in the SL1344 reference chromosome (NCBI accession FQ312003.1) is indicated along with the allele change. If blank, the region of the genome contains no mutations. Percentages indicate the fraction of isolates, which show a particular mutation.
(XLSX)

S6 Table Summary of variants in the ancestral wild-type S. Typhimurium strain or *S*.Tm* strains used in this study. Non-synonymous mutations in SB300 (SL1344 WT; *S*.Tm) and M2730 (SL1344 Δ*ssaV*; *S*.Tm*) used to infected mice in this study are compared to the wild-type *S*. Typhimurium SL1344 reference genome NCBI accession FQ312003.1. The SipC phenotype in our colony protein blot assay (as a proxy for TTSS-1 expression) is shown; + and blue color code indicates that SipC is detected in the colony protein blot assay. In order to generate S.Tm* (termed M2730 in the laboratory strain collection), we have used P22 transduction followed by removal of the resistance cassette (e.g., *ssaV*). The deletion is indicated by the “Δ” symbol in S6 Table.
(XLSX)

S7 Table ASVs differentially enriched on days 120 p.i. **and 160 p.i. in the feces of wild-type *S*. Typhimurium-infected control mice (no co-housing) compared to mice co-housed with a CON**^**X**^ **mouse.** ASVs were identified in samples from mice on day 120 and 160 p.i. (*n* = 6 mice for each, wild-type *S*. Typhimurium kept under hygienic isolation and mice co-housed with CON^X^ animals; from Fig 3). Measures of within-sample diversity, beta-diversity and order or phylum diversity are shown in Figs 3E and S9. Significantly enriched ASVs, determined by a Wald test are ranked by log_2_ fold change (*S*.Tm and *S*.Tm + CON^X^). Shown are ASVs that are significantly more abundant in *S*.Tm with a p_adjusted_ cut-off of *p* < 0.05 and a log_2_ fold change cut-off of >1 (indicated as a blue shade in the table). BaseMean represents the average read counts that were normalized by size factors over all samples.
(XLSX)

S8 Table ASVs differentially enriched in the feces of wild-type *S*. **Typhimurium-infected control mice (no co-housing) or mice co-housed with a CON**^**X**^ **(on day 160 p.i.) compared to CON**^**X**^ **mice on day 0 p.i.** ASVs were identified in samples from mice on day 160 p.i. (*n* = 6 mice for each; from Fig 3). Significantly enriched ASVs, determined by a Wald test are ranked by log_2_ fold change (*S*.Tm + CON^X^ over CON^X^). Shown are ASVs that are significantly more abundant in co-housed mice with a p_adjusted_ cut-off of *p* < 0.05 and a log_2_ fold change cut-off of >1 (indicated as a blue shade in the table). BaseMean represents the average read counts that were normalized by size factors over all samples.
(XLSX)

S9 Table ASVs differentially enriched in the feces of mice infected with wild-type *S*. Typhimurium compared to mice infected with *S*.Tm* on days 0, 10, and 70 p.i. ASVs were identified in samples from mice on days 0, 10, and 70 p.i. (*n* = 6 mice for each; from Fig 1B–1D). Measures of within-sample diversity, beta-diversity, and order or phylum or family diversity are shown in Figs 6C and S13. Significantly enriched ASVs, determined by a Wald test are ranked by log_2_ fold change (*S*.Tm over *S*.Tm*). Shown are ASVs that are significantly more abundant in *S*.Tm with a p_adjusted_ cut-off of *p* < 0.05 and a log_2_ fold change cut-off of >1 (indicated as a blue shade in the table). BaseMean represents the average read counts that were normalized by size factors over all samples.
(XLSX)

S10 Table ASVs differentially enriched in the feces of mice infected with *S*.Tm* on day 70 p.i. **(from Fig 1B–1D) to CON**^**X**^ **mice on day 0 p.i. from Fig 3.** ASVs were identified in samples from mice on day 70 p.i. (*n* = 6 mice for each; Fig 1B–1D) and on day 0 (*n* = 6; donor mice from Fig 3). Significantly enriched ASVs, determined by a Wald test are ranked by log_2_ fold change (*S*.Tm* over CON^X^). Shown are ASVs that are significantly more abundant in *S*. Typhimurium* infected mice with a p_adjusted_ cut-off of *p* < 0.05 and a log_2_ fold change cut-off of >1 (indicated as a blue shade in the table). BaseMean represents the average read counts that were normalized by size factors over all samples.
(XLSX)

## Abbreviations

AAM: anaerobic Akkermansia medium
BHI: brain heart infusion
CR: colonization resistance
EB: elution buffer
GF: germ-free
LB: lysogeny broth
LCM: low complexity microbiota
mLN: mesenteric lymph node
NGS: normal goat serum
PCoA: principal coordinate analysis
RT: room temperature
SCFA: short chain fatty acid
TTSS: type III secretion system
