# Supplementary material for: The microbiota conditions a gut milieu that selects for wild-type Salmonella Typhimurium virulence
Source: PLoS Biol. 2023 Aug 31;21(8):e3002253. doi: 10.1371/journal.pbio.3002253 (PMC10499267; doi:10.1371/journal.pbio.3002253)
Supplement: S1 Text — (DOCX) [file pbio.3002253.s002.docx]

**Supplementary discussion A:**

**Within-host adaptation to the gut through nonsynonymous mutations in coding sequences**

In our experiments, within-host evolution was readily observed through our colony blot assay (**Figs 1D and S4)**. We previously demonstrated that *hilD* is the genetic locus most commonly disrupted in attenuated mutants selected for within the infected host [1]. However, *hilC* mutants can also emerge and these mutants yield “dim” SipC expression signals in our assay [2]. We sought to confirm this observation in these experiments. But due to the longer duration of our current experiments, we also expected that additional mutations could be selected for during the 70 days of infection. Therefore, we performed whole-genome sequencing on isolates obtained from three distinct time-points from the experiment:

1. A time-point corresponding to day 10-13 p.i. for both wild-type *S*. Typhimurium and *S.*Tm*. We expected, that this should yield results equivalent to those from our previous work, which had studied the within-host evolution of *S*.Tm* for up to 10 days.
2. A time-point corresponding to day 47-70 for both wild-type *S*. Typhimurium and *S.*Tm*. This would represent the mutations prevalent in the gut luminal *Salmonella* population before we have started the microbiota transfer experiment (as described, blow in **Fig 3**)
3. A time-point corresponding to the end of our microbiota transfer experiment into wild-type *S*. Typhimurium infected mice (or the control groups w/o microbiota transfer) which is depicted in **Fig 3** (day 157-160 p.i.).

Importantly, we chose isolates based on their SipC phenotype in our colony protein blot assay. The SipC phenotypes were classified as either “strong” (indicated by a “+” sign and highlighted in blue), “dim” (indicated by a “+/-“sign and highlighted in yellow), or “absent” (indicated by a “-“ sign and highlighted in red) (**S1-S6 Tables**). At each time-point, we sequenced at least 10 clones from mice infected with wild-type *S*. Typhimurium or *S.*Tm*, covering multiple independent mice, independent cages, and independent experiments, to allow the identification of adaptive mutations.

As expected, we uncovered several mutations that occurred in genes in the HilD-regulon [3]. Non-synonymous mutations in *hilD* were most frequently observed (**Figs 1E and S8 and S1-S6 Tables**), but we also observed mutations in *hilC*, *barA*, and *sirA/uvrY* (**S1-S5 Tables**). Importantly, mutations in genes that occurred in the HilD-regulon correlated well with the SipC phenotype determined through colony blot in most cases (either SipC dim or absent phenotypes; **S1-S6 Tables**). 82% of all clones without detectable SipC had such *hilD* mutations (**Fig 1E**), while 18% did not. Some of the latter clones had mutations in other parts of the regulon. However, we cannot exclude that some of the clones "without detectable SipC" were actually false negatives and attributable to the limited signal-to-noise ratio of our colony protein blot assay. Therefore, we needed to perform follow-up experiments with defined mutants that carry antibiotic resistance markers to verify the within-host selection for mutants of interest. Regardless, *hilD* remained the most frequently mutated gene in the analyzed strains, and our data suggested that within-host adaptation through virulence attenuation can also proceed through loss of other regulators in the HilD-regulon.

We then explored if additional adaptive mutations might have been selected for across all time-points in mice infected with *S.*Tm or *S.*Tm*. We observed the progressive accumulation of mutations in clones of *S.*Tm over time (**Fig 3 and S1-S3 Tables**), likely as a result of the selection for mutants (which randomly arise during the growth of the *Salmonella* population) within the infected host. At day 160 p.i., the genes most frequently mutated (besides the HilD-regulon) in the sequenced clones were genes associated with metabolism (e.g. *glpT, melR or melB,* and *ybaO)*, O-antigen or LPS biosynthesis (e.g. *oafA, rfc/wzyB, rfaK/waaK,* and *rfbP/wbaP*), or flagella/motility/chemotaxis (e.g. *fliC. fliB, tsr,* and *flhB*) (**Fig 3**). Mutations in genes associated with metabolism such as *glpT*, encoding a glycerol-3-phosphate transporter [4], *melR* or *melB*, encoding genes important in melibiose utilization [5], and *ybaO*, encoding a regulator that controls L-cysteine detoxification [6], imply the importance of fine-tuning metabolism to optimize during long-term growth in the chronically inflamed gut (inflammation shown in **Figs 1B, 3D and S3**). The nutrient landscape could be different, or new toxic intermediates or by-products of other metabolic pathways may accumulate, which would create a milieu that selects for mutants that are better adapted to this milieu, than the original wild-type strain. For example, such adaptive mutations may occur in genes encoding transporters that import such toxic compounds (or their precursors), or genes encoding pleiotropic regulators that confer a substantial fitness cost. Further work will be needed to elucidate the relative importance of this in adaptation to the inflamed gut.

Adaptive secretory IgA responses account for the observed mutants in LPS O-antigen genes (**Fig 1E** and **S1-S3 Tables**). In vaccinated mice, *S.*Tm that modified their O-antigen by mutations in *oafA* or *rfc/wzyB* were shown to be selected for in the gut lumen their wild-type counterparts [7]. This is because intestinal IgA is strongly directed towards the LPS O-antigen of *S*.Tm [8-10]. Correspondingly, the *oafA, rfc/wzyB, rfaK/waaK,* and *rfbP/wbaP* mutants detected in our experiments likely resulted from selection by the adaptive immune response, which commences by days 10-20 of infection [8-10]. Flagella can also serve as a target for the adaptive immune response [11]. This could explain why we have found diverse mutations (sometimes even in the same clone) in *fliC* encoding the flagellin of *S.*Tm (**S2-S3 Tables).**

Strikingly, in one mouse, all clones contained mutations in *mutS*, encoding a DNA mismatch repair protein. Mutants in *mutS* are known to increase mutation rates by 100-fold (termed “mutators”) [12]. Correspondingly, in clones from this mouse, a far greater number of genes were mutated than in isolates from other mice that featured an intact *mutS* gene (**S1-S3 Tables**). Increased mutation rates appear to speed up the emergence of clones with adaptive mutations that would be beneficial in a new environment, but at the risk that deleterious mutations can hitchhike in the background of advantageous ones, as demonstrated previously for adaptation of *E. coli* to the mouse gut [13]. The sheer number of genes mutated in these strains suggests the likelihood of some deleterious mutations which would be strongly attenuated after microbiota transfer (as performed later in this study) or after transmission.

In general, more mutations accumulated in wild-type *S*. Typhimurium compared to *S.*Tm* over time. In fact, at day 70 p.i., fewer non-synonymous mutations were found in clones isolated from mice infected with *S.*Tm* (median 2 mutations; **S5 Table**). This could likely be explained by a lower population size (compared to the wild-type *S*. Typhimurium infections) which decreases the likelihood of mutations emerging, or a more competitive environment that selects against such strongly mutated strains. While both may likely contribute, the striking prevention of long-term colonization by *hilD* mutants in *S.*Tm* infected mice **(Figs 1C and S4**) suggests that the regrowing microbiota (**Fig 6**) may provide a selective pressure against key adaptive mutations observed in wild-type *S*. Typhimurium infected mice which remain under hygienic isolation (**S1-S3 Tables**). In line with this, at day 10-13 post infection (when the microbiota are equally disturbed in the *S*.Tm* and the wild-type infected mice), there was no significant difference in the number of genes mutated in clones from wild-type *S*. Typhimurium or *S.*Tm*-infected mice (**S1 and S4 Tables**). However, there were some differences in the types and the frequencies of adaptive mutations between the two strains (**S1 and S4 Tables**). This may be related to differences in the gut-luminal milieu that are caused by the higher disease intensity caused by the wild-type *S*. Typhimurium infection as compared to *S*.Tm*, or by its stronger impact on the resident microbiota (as described later in this work). Further work will be needed to elucidate exactly how the different types of host responses shape the selective landscape.

Altogether, our analysis of the mutants re-isolated from the infected host gut under hygienic isolation highlights some key adaptive features, and our observations hinted that this selection for mutants with reduced virulence (and their fecal shedding over long periods of time) might be related to the microbiota. Importantly, mutations in genes identified in our experiments show a striking overlap with the spectrum of mutations observed in natural or clinical isolates. For example, *hilD*, *melR*, and mutants relevant for O-antigen modification have been observed in patients [14] and in bioinformatic analysis of more than 100,000 *Salmonella* genomes [15]. Moreover, the latter study found evidence for the positive selection of *tsr, glpT, hilC, barA, sirA/uvrY* in some small natural niche. This strong overlap with the mutations selected for during the infection of our antibiotic pretreated mice suggests that this mutant-selecting natural niche might be related to cases of severe microbiota disruption (for example in cases with pronounced inflammatory diarrhea) or by microbiotas lacking relevant competitor strains.

**Supplementary Discussion B**:

**Microbiota engineering can be an effective tool to control virulence evolution in enteric pathogens, but it may also open the way to "super-virulent" bugs**

As antibiotic resistance is on the rise, leveraging the microbiota becomes an attractive alternative path[16] . In the case of antibiotics, they are useful in limiting the disease, but inefficient killing can select for persisters and resistant bacteria, and promote the emergence of “super-resistant bugs” resistant to several drugs [17-21]. Analogous to this, it seems reasonable to speculate that the emergence of enteric pathogens such as *Salmonella* spp. and the maintenance of their virulence may result from selection within hosts. Our data suggest that this selection is critically affected by the gut microbiota. It should be emphasized that this selection for virulence is not an evolved function of the microbiota. Much rather, it is the microbiota's effects on the gut-luminal milieu, which co-incidentally affect the pathogen's virulence evolution. Our data suggest that microbiota transfer after severe microbiota disruption (as in **Fig 5**) and hosts with intermediate levels of CR might (as in **Figs 7, S6** and **S18**) offer selective conditions, as pathogen growth is possible (to higher or lesser extents), while the microbiota can condition the gut-luminal milieu to select against *hilD* mutants or displace *hilD* mutant-dominated pathogen populations at later phases of an infection.

Cases of incomplete CR may emerge after transient perturbations such as abrupt changes of diet composition, industrial farming (which is often associated with antibiotic exposure or food-shifts) and antibiotic therapies[22]. Of note, such therapeutic antibiotic treatments are normally much milder than the antibiotic treatment that is applied in our streptomycin pre-treatment model, which applies one very high dose of streptomycin to permit *Salmonella* colonization of the gut lumen up to carrying capacity[23]. This does not happen in mice that are treated with lower doses of streptomycin. Finally, newborns may also represent cases of incomplete CR, as they are still lacking a mature microbiota conferring high level CR. Identifying natural niches where incomplete CR of a given microbiota or microbiota transfer after the disruption of CR will affect the selection for wild-type *Salmonella* virulence will be an interesting task for future research.

Our data may also provide some insights about the importance of the HilD regulon and of the gut microbiota in *Salmonella* virulence evolution. During its evolution from an ancestral *E. coli* strain (likely a part of the microbiome) to “modern *Salmonella*”[24], the bacterium has acquired SPI-1 and SPI-2 sequentially. Our data suggests that it has been important that the virulence factor genes were placed under the control of response circuits responsive to various microbiota-derived signals. This had two important effects. First, the TTSS-1 and TTSS-2, which are encoded in SPI-1 and SPI-2 provided a selective advantage by enabling *Salmonella* spp. to elicit gut inflammation and thereby alleviate CR at least in some host animals (possibly those with incomplete CR, as discussed above). At the same time, the control by the HilD regulon has allowed to alleviate (or at least minimize) the costs associated with virulence factor expression by limiting the gene expression to those moments (or sites), where they are needed to successfully navigate the complex environment of the host's intestine (via flagella-driven motility) and interact with the cells of the host's tissue (via TTSS-1 or TTSS-2). It is conceivable that placing the virulence factors under the control of the HilD regulon was critical to minimize the costs, maximize fitness benefits and thereby prevent (or at least minimize) the selection against mutants with reduced virulence (like the *hilD* mutants studied, here). Deciphering the signals that control HilD regulon expression may pave the way to better control over pathogen evolution. However, before implementing such microbiota engineering strategies to fend off enteric pathogens, it would seem wise to gain a deeper understanding of the complex host-microbiota-pathogen interactions which are governing this system. This may help to avoid accidentally creating niches selecting for “super-virulent” enteropathogens.

**Supplementary Discussion C**:

**Microbiota members that may contribute to resolution of enteropathy and the clearance of gut-luminal pathogen populations**

In the current work, we show that antibiotic pretreated mice infected with wild-type *S*. Typhimurium will feature long-lasting microbiota perturbation and develop long-lasting enteropathy when kept under strict hygienic isolation, while mice infected with *S.*Tm* return to a state of remission (**Figs 1, 3, 4, 6, S9 and S13**). The transfer of a complex microbiota displaced the gut-luminal *Salmonella* population selected for during wild-type *S*. Typhimurium infection and accelerated the resolution of enteropathy. This suggested that the gut microbiota or certain microbiota members may condition the gut luminal milieu and thereby facilitate the termination of pathogen gut colonization, the selection for the wild-type pathogen or mutants with reduced virulence and the resolution of the enteric disease pathology. Identifying specific microbiota members that are involved in either process could be of interest for designing therapeutic approaches for diseases associated with chronic gut inflammation that can occur after episodes of acute enteropathogen infection [25].

To reveal the microbiota members and possible mechanisms by which they restrict long-term colonization by *hilD* mutants, we analyzed the communities in detail and designed microbiota gavage experiments (**S15 and S16 Figs**). Our microbiota analyses have indicated that the genera belonging to Lachnospirales, Oscillospirales, and Erysipelotrichales might enhance the selection against *hilD* mutants. Among these, members of Lachnospirales and Oscillospirales were shown to be butyrate producing taxa and might be associated with resolution of *Salmonella*-inflicted enteropathy, as butyrate production is associated with induction of regulatory T-cells [26-30]. Besides, several reports indicated the involvement of butyrate and other SCFAs (acetate and propionate) in regulation of virulence expression in *Salmonella* strains [31-33]. Strikingly, infection with wild-type *S*. Typhimurium resulted in the drop of propionate and butyrate concentrations below the detection limit, and significantly reduced the acetate concentrations (CON^X^ vs *S*.Tm + Strep. + CON^X^ at day 40 p.i.; **S14 Fig**). This is likely caused by the pronounced disruption of the microbiota community structure (and the associated change in microbiota physiology) that we have observed in the wild-type *S*. Typhimurium infected mice (**Figs 3E**, 6, **S9** **and S13**). Our data suggest that these effects of pronounced microbiota disruption (and its failure to become re-established by transmission under our conditions of hygienic isolation) are critical also for the selection for *hilD* mutants and the failure to displace the mutant-dominated *Salmonella* populations which arise during the course of the infection. In our study, we used several approaches to experimentally demonstrate the above hypotheses. However, current techniques that we would employ to study specific functions of particular gut microbe strains were not sufficient to derive conclusive data about the identity of the key microbiota strains, how they control mucosal disease, condition the gut-luminal milieu or contribute to excluding the pathogen or its mutated populations. Therefore, our findings have remained correlative in this regard and further work will be required to gain a mechanistic understanding. Thus, we are left with speculation if we simply failed to identify the key microbiota strain, or if emergent properties of a community when it contains specific members is more important than any individual strain.

Regardless, our data show that streptomycin treatment and the infection with either wild-type *S*. Typhimurium or S.Tm* depletes the members of the order Bacteroidales, communities belonging to the orders Lactobacillales and Erysipelotrichales are increasing in abundance (**S9 and S13 Figs**). This suggests that the gut-luminal milieu of the infected gut has surprisingly specific effects on different members of the microbiota. Instead of indiscriminate suppression of all gut microbiota, some may dwell in this environment. Understanding the consequences of these community shifts could be of interest for many fields. For example, Muribaculaceae (belonging to the order Bacteroidales) were previously reported to be involved in colonization resistance to *Salmonella* infection [34] and a recent study revealed a potential functional importance of Muribaculaceae in the mammalian gut [35]. Hence, we propose that further investigation of this family might reveal important insights regarding strategies used by microbiome members to confer colonization resistance to enteric pathogens. On the other hand, we observed an increased abundance of members of the genera *Dubosiella, Faecalibaculum, Turicibacter* (of Erysipelotrichales) and *Lactobacillus and Enterococcus* (of Lactobacillales) in the inflamed gut, suggesting that these members might condition the gut in an "unfavorable" fashion which may promote colonization by *Salmonella* spp. and possibly other enteropathogens. Interestingly, lactate produced by *Lactobacillus* spp. was shown to promote *S*. Typhimurium growth in the inflamed gut[36]. Strikingly, levels of lactate were elevated in the infected mice (**S14 Fig**), in line with an increase of order Lactobacillales in the inflamed gut. Lactate fermentation by the infected gut tissue may also contribute to the elevated lactate concentrations[36]. Regardless, it is tempting to speculate that resolution or propagation of inflammation and pathogen elimination from the gut lumen may be context-dependent and could be achieved by several different phylotypes and that these may function in conjunction with the altered host physiology of the diseased gut.

Our experiments in the current work were limited to longitudinal sampling which prevented us to investigate the subtle differences in the microbiota compositions and spatial organization of these members with respect to the *Salmonella* cells in the mucosa during the different stages of the enteric infection. We suggest that a detailed analysis of microbiota species in the lumen compared to those in tissue-associated niches might be more informative and could point to specific roles of some microbiota members. Besides, our knowledge about mechanisms used by microbiota to establish a long-lasting reservoir and the identity of factors which could force re-establish such niches is very limited. Our current models and the microbiota transfer experiments provide a working platform to tackle these questions. Developing techniques for assessing the localization of the relevant microbiota members in 3D in the gut before [37] the infection, during acute disease and after its resolution could shed light on how the microbiota could maintain their niches in the gut. This way, we might learn from an extreme case of colitis about how to eliminate some unwanted microbes from the gut and develop microbiota engineering approaches for the future to design optimized "personal microbiota therapies".

There has been extensive research in the field of inflammatory bowel disease (IBD) to develop possible therapeutics which are based on the possible benefits of microbiome-modulating interventions [38-41]. We hypothesize that members of the microbiota discussed here could have a potential as therapeutics in IBD patients. In the cases of chronic inflammation following a diarrheal disease, loss of certain microbiota members could be the underlying reason for delayed resolution from gut inflammation. In our model system, the prolonged enteropathy that resulted from the wild-type *S*. Typhimurium infection was associated with the depletion of a group of microbiota families. The same may apply to IBD cases that develop after an episode of acute infection. Therefore, we propose further studies testing these members as therapeutics or for testing prevention strategies in IBD models.

**References:**

1. Diard M, Garcia V, Maier L, Remus-Emsermann MN, Regoes RR, Ackermann M, et al. Stabilization of cooperative virulence by the expression of an avirulent phenotype. Nature. 2013;494(7437):353-6. Epub 2013/02/22. doi: 10.1038/nature11913

nature11913 [pii]. PubMed PMID: 23426324.

2. Bakkeren E, Dolowschiak T, M RJD. Detection of Mutations Affecting Heterogeneously Expressed Phenotypes by Colony Immunoblot and Dedicated Semi-Automated Image Analysis Pipeline. Front Microbiol. 2017;8:2044. doi: 10.3389/fmicb.2017.02044. PubMed PMID: 29104568; PubMed Central PMCID: PMCPMC5655795.

3. Erhardt M, Dersch P. Regulatory principles governing Salmonella and Yersinia virulence. Front Microbiol. 2015;6:949. doi: 10.3389/fmicb.2015.00949. PubMed PMID: 26441883; PubMed Central PMCID: PMCPMC4563271.

4. Hengge R, Larson TJ, Boos W. sn-Glycerol-3-phosphate transport in Salmonella typhimurium. J Bacteriol. 1983;155(1):186-95. Epub 1983/07/01. doi: 10.1128/JB.155.1.186-195.1983. PubMed PMID: 6408060; PubMed Central PMCID: PMCPMC217668.

5. Ethayathulla AS, Yousef MS, Amin A, Leblanc G, Kaback HR, Guan L. Structure-based mechanism for Na(+)/melibiose symport by MelB. Nat Commun. 2014;5:3009. Epub 2014/01/07. doi: 10.1038/ncomms4009. PubMed PMID: 24389923; PubMed Central PMCID: PMCPMC4026327.

6. Shimada T, Tanaka K, Ishihama A. Transcription factor DecR (YbaO) controls detoxification of L-cysteine in Escherichia coli. Microbiology (Reading). 2016;162(9):1698-707. Epub 2016/07/21. doi: 10.1099/mic.0.000337. PubMed PMID: 27435271.

7. Diard M, Bakkeren E, Lentsch V, Rocker A, Bekele NA, Hoces D, et al. A rationally designed oral vaccine induces immunoglobulin A in the murine gut that directs the evolution of attenuated Salmonella variants. Nat Microbiol. 2021;6(7):830-+. doi: 10.1038/s41564-021-00911-1. PubMed PMID: WOS:000655565300001.

8. Moor K, Diard M, Sellin ME, Felmy B, Wotzka SY, Toska A, et al. High-avidity IgA protects the intestine by enchaining growing bacteria. Nature. 2017. doi: 10.1038/nature22058. PubMed PMID: 28405025.

9. Endt K, Stecher B, Chaffron S, Slack E, Tchitchek N, Benecke A, et al. The microbiota mediates pathogen clearance from the gut lumen after non-typhoidal Salmonella diarrhea. Plos Pathog. 2010;6(9):e1001097. doi: 10.1371/journal.ppat.1001097. PubMed PMID: 20844578; PubMed Central PMCID: PMCPMC2936549.

10. Macpherson AJ, Yilmaz B, Limenitakis JP, Ganal-Vonarburg SC. IgA Function in Relation to the Intestinal Microbiota. Annu Rev Immunol. 2018;36:359-81. doi: 10.1146/annurev-immunol-042617-053238. PubMed PMID: WOS:000433486000014.

11. Tran HQ, Ley RE, Gewirtz AT, Chassaing B. Flagellin-elicited adaptive immunity suppresses flagellated microbiota and vaccinates against chronic inflammatory diseases. Nat Commun. 2019;10(1):5650. Epub 2019/12/13. doi: 10.1038/s41467-019-13538-y. PubMed PMID: 31827095; PubMed Central PMCID: PMCPMC6906489.

12. Sniegowski PD, Gerrish PJ, Lenski RE. Evolution of high mutation rates in experimental populations of E. coli. Nature. 1997;387(6634):703-5.

13. Giraud A, Matic I, Tenaillon O, Clara A, Radman M, Fons M, et al. Costs and benefits of high mutation rates: adaptive evolution of bacteria in the mouse gut. Science. 2001;291(5513):2606-8.

14. Marzel A, Desai PT, Goren A, Schorr YI, Nissan I, Porwollik S, et al. Persistent Infections by Nontyphoidal Salmonella in Humans: Epidemiology and Genetics. Clinical infectious diseases : an official publication of the Infectious Diseases Society of America. 2016;62(7):879-86. doi: 10.1093/cid/civ1221. PubMed PMID: 26740515; PubMed Central PMCID: PMC4787607.

15. Cherry JL. Selection-Driven Gene Inactivation in Salmonella. Genome Biol Evol. 2020;12(3):18-34. doi: 10.1093/gbe/evaa010. PubMed PMID: 32044996; PubMed Central PMCID: PMCPMC7144821.

16. Sanders ME. Probiotics: Considerations for human health. Nutr Rev. 2003;61(3):91-9. doi: DOI 10.1301/nr.2003.marr.91-99. PubMed PMID: WOS:000186295200002.

17. Bakkeren E, Diard M, Hardt WD. Evolutionary causes and consequences of bacterial antibiotic persistence. Nat Rev Microbiol. 2020;18(9):479-90. doi: 10.1038/s41579-020-0378-z. PubMed PMID: WOS:000558806900005.

18. Nikaido H. Multidrug Resistance in Bacteria. Annu Rev Biochem. 2009;78:119-46. doi: 10.1146/annurev.biochem.78.082907.145923. PubMed PMID: WOS:000268069200007.

19. Levin-Reisman I, Ronin I, Gefen O, Braniss I, Shoresh N, Balaban NQ. Antibiotic tolerance facilitates the evolution of resistance. Science. 2017;355(6327):826-30. doi: 10.1126/science.aaj2191. PubMed PMID: WOS:000395127600038.

20. Peko L, Barakat-Johnson M, Gefen A. Protecting prone positioned patients from facial pressure ulcers using prophylactic dressings: A timely biomechanical analysis in the context of the COVID-19 pandemic. Int Wound J. 2020;17(6):1595-606. doi: 10.1111/iwj.13435. PubMed PMID: WOS:000544974800001.

21. Berni A, Malandrino D, Parenti G, Maggi M, Poggesi L, Peri A. Hyponatremia, IL-6, and SARS-CoV-2 (COVID-19) infection: may all fit together? J Endocrinol Invest. 2020;43(8):1137-9. doi: 10.1007/s40618-020-01301-w. PubMed PMID: WOS:000535418300001.

22. Kreuzer M, Hardt WD. How Food Affects Colonization Resistance Against Enteropathogenic Bacteria. Annu Rev Microbiol. 2020;74:787-813. doi: 10.1146/annurev-micro-020420-013457. PubMed PMID: WOS:000613937400037.

23. Barthel M, Hapfelmeier S, Quintanilla-Martinez L, Kremer M, Rohde M, Hogardt M, et al. Pretreatment of mice with streptomycin provides a Salmonella enterica serovar typhimurium colitis model that allows analysis of both pathogen and host. Infect Immun. 2003;71(5):2839-58. doi: 10.1128/Iai.71.5.2839-2858.2003. PubMed PMID: WOS:000182501500061.

24. Diard M, Hardt WD. Evolution of bacterial virulence. Fems Microbiol Rev. 2017;41(5):679-97. doi: 10.1093/femsre/fux023. PubMed PMID: WOS:000412014200007.

25. Axelrad JE, Olen O, Askling J, Lebwohl B, Khalili H, Sachs MC, et al. Gastrointestinal Infection Increases Odds of Inflammatory Bowel Disease in a Nationwide Case-Control Study. Clin Gastroenterol H. 2019;17(7):1311-+. doi: 10.1016/j.cgh.2018.09.034. PubMed PMID: WOS:000468432400024.

26. Meehan CJ, Beiko RG. A phylogenomic view of ecological specialization in the Lachnospiraceae, a family of digestive tract-associated bacteria. Genome Biol Evol. 2014;6(3):703-13. Epub 2014/03/15. doi: 10.1093/gbe/evu050. PubMed PMID: 24625961; PubMed Central PMCID: PMCPMC3971600.

27. Arpaia N, Campbell C, Fan X, Dikiy S, van der Veeken J, deRoos P, et al. Metabolites produced by commensal bacteria promote peripheral regulatory T-cell generation. Nature. 2013;504(7480):451-5. doi: 10.1038/nature12726. PubMed PMID: 24226773; PubMed Central PMCID: PMCPMC3869884.

28. Furusawa Y, Obata Y, Fukuda S, Endo TA, Nakato G, Takahashi D, et al. Commensal microbe-derived butyrate induces the differentiation of colonic regulatory T cells. Nature. 2013;504(7480):446-50. doi: 10.1038/nature12721. PubMed PMID: 24226770.

29. Smith PM, Howitt MR, Panikov N, Michaud M, Gallini CA, Bohlooly YM, et al. The microbial metabolites, short-chain fatty acids, regulate colonic Treg cell homeostasis. Science. 2013;341(6145):569-73. Epub 2013/07/06. doi: 10.1126/science.1241165. PubMed PMID: 23828891; PubMed Central PMCID: PMCPMC3807819.

30. Atarashi K, Tanoue T, Shima T, Imaoka A, Kuwahara T, Momose Y, et al. Induction of colonic regulatory T cells by indigenous Clostridium species. Science. 2011;331(6015):337-41. Epub 2011/01/06. doi: 10.1126/science.1198469. PubMed PMID: 21205640; PubMed Central PMCID: PMCPMC3969237.

31. Hung CC, Garner CD, Slauch JM, Dwyer ZW, Lawhon SD, Frye JG, et al. The intestinal fatty acid propionate inhibits Salmonella invasion through the post-translational control of HilD. Mol Microbiol. 2013;87(5):1045-60. doi: 10.1111/mmi.12149. PubMed PMID: WOS:000315399500008.

32. Hockenberry AM, Micali G, Takacs G, Weng J, Hardt WD, Ackermann M. Microbiota-derived metabolites inhibit Salmonella virulent subpopulation development by acting on single-cell behaviors. Proc Natl Acad Sci U S A. 2021;118(31). Epub 2021/08/01. doi: 10.1073/pnas.2103027118. PubMed PMID: 34330831.

33. Golubeva YA, Ellermeier JR, Chubiz JEC, Slauch JM. Intestinal Long-Chain Fatty Acids Act as a Direct Signal To Modulate Expression of the Salmonella Pathogenicity Island 1 Type III Secretion System. Mbio. 2016;7(1). doi: ARTN e02170-15

10.1128/mBio.02170-15. PubMed PMID: WOS:000373933100030.

34. Jacobson A, Lam L, Rajendram M, Tamburini F, Honeycutt J, Pham T, et al. A Gut Commensal-Produced Metabolite Mediates Colonization Resistance to Salmonella Infection. Cell Host Microbe. 2018;24(2):296-307 e7. doi: 10.1016/j.chom.2018.07.002. PubMed PMID: 30057174; PubMed Central PMCID: PMCPMC6223613.

35. Lagkouvardos I, Lesker TR, Hitch TCA, Galvez EJC, Smit N, Neuhaus K, et al. Sequence and cultivation study of Muribaculaceae reveals novel species, host preference, and functional potential of this yet undescribed family. Microbiome. 2019;7(1):28. doi: 10.1186/s40168-019-0637-2. PubMed PMID: 30782206; PubMed Central PMCID: PMCPMC6381624.

36. Gillis CC, Hughes ER, Spiga L, Winter MG, Zhu WH, de Carvalho TF, et al. Dysbiosis-Associated Change in Host Metabolism Generates Lactate to Support Salmonella Growth. Cell Host & Microbe. 2018;23(1):54-+. doi: 10.1016/j.chom.2017.11.006. PubMed PMID: WOS:000419707000010.

37. Nguyen J, Pepin DM, Tropini C. Cause or effect? The spatial organization of pathogens and the gut microbiota in disease. Microbes Infect. 2021;23(6-7):104815. Epub 2021/03/30. doi: 10.1016/j.micinf.2021.104815. PubMed PMID: 33775859.

38. Khan I, Ullah N, Zha L, Bai Y, Khan A, Zhao T, et al. Alteration of Gut Microbiota in Inflammatory Bowel Disease (IBD): Cause or Consequence? IBD Treatment Targeting the Gut Microbiome. Pathogens. 2019;8(3). Epub 2019/08/16. doi: 10.3390/pathogens8030126. PubMed PMID: 31412603; PubMed Central PMCID: PMCPMC6789542.

39. Glassner KL, Abraham BP, Quigley EMM. The microbiome and inflammatory bowel disease. J Allergy Clin Immunol. 2020;145(1):16-27. Epub 2020/01/09. doi: 10.1016/j.jaci.2019.11.003. PubMed PMID: 31910984.

40. Kostic AD, Xavier RJ, Gevers D. The microbiome in inflammatory bowel disease: current status and the future ahead. Gastroenterology. 2014;146(6):1489-99. Epub 2014/02/25. doi: 10.1053/j.gastro.2014.02.009. PubMed PMID: 24560869; PubMed Central PMCID: PMCPMC4034132.

41. Damman CJ, Miller SI, Surawicz CM, Zisman TL. The microbiome and inflammatory bowel disease: is there a therapeutic role for fecal microbiota transplantation? Am J Gastroenterol. 2012;107(10):1452-9. Epub 2012/10/05. doi: 10.1038/ajg.2012.93. PubMed PMID: 23034604.
