# Supplementary figures and images for: The microbiota conditions a gut milieu that selects for wild-type Salmonella Typhimurium virulence
Source: PLoS Biol. 2023 Aug 31;21(8):e3002253. doi: 10.1371/journal.pbio.3002253 (PMC10499267; doi:10.1371/journal.pbio.3002253)

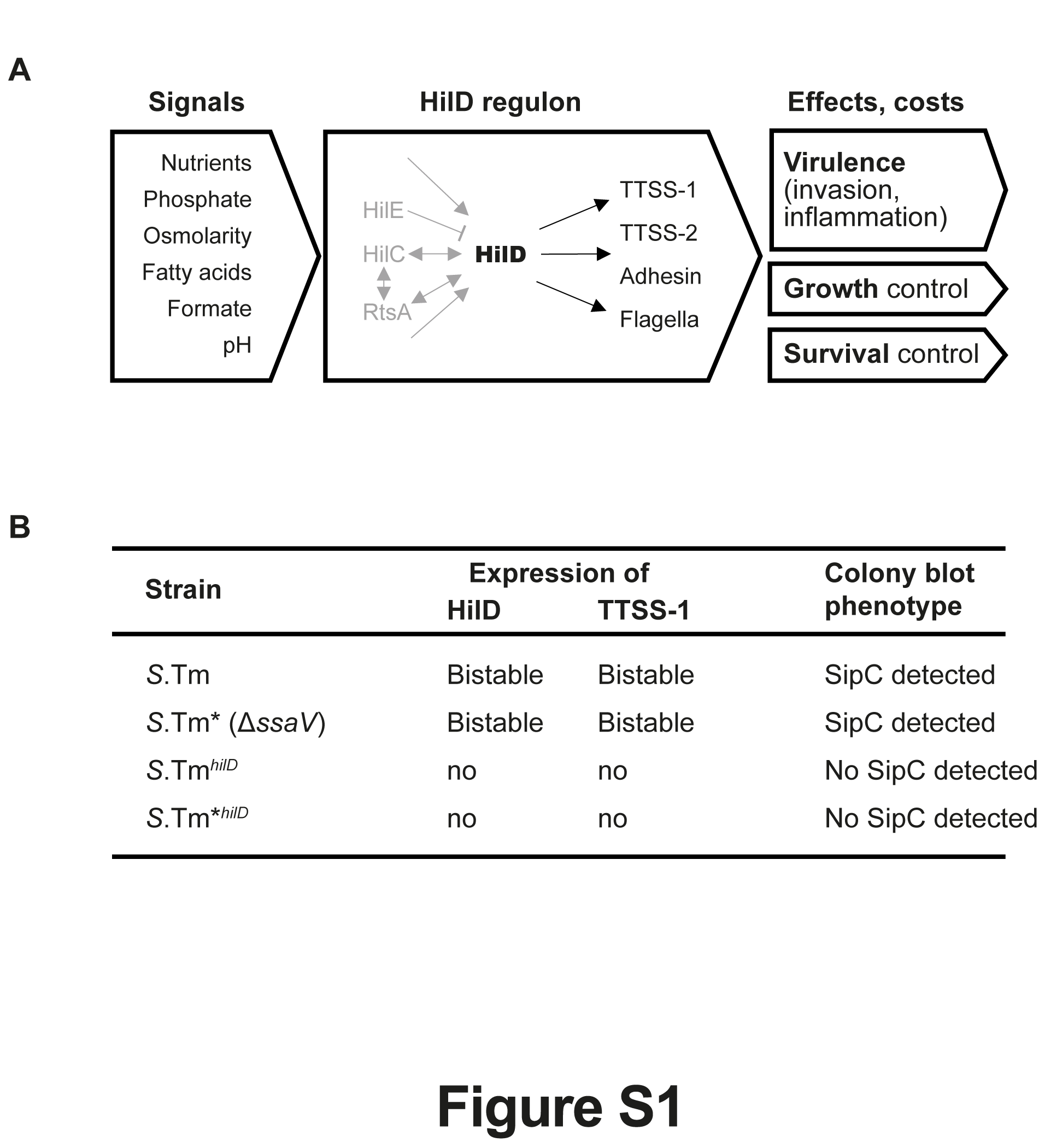

Supplement: S1 Fig — (A) The HilD regulon (center) has a complex architecture (for details, see [34,35]) and computes responses to a variety of environmental signals. Many of these signals are derived or controlled by the microbiota and the host. They provide environmental cues for controlled expression of S. Typhimurium virulence factors and physiological adaptations ensuring growth and survival such that the associated costs occur only at those moments of the infection cycle when the respective virulence factors are needed. The HilD regulon limits their costly expression to the gut lumen and shuts them off after mucosa invasion [36]. Moreover, the HilD regulon limits these costs of virulence expression to a subpopulation of the gut-luminal S. Typhimurium cells by ensuring that only a subset of the pathogen population is expressing hilD (bistable expression) [28,29]. Thus, in hosts with severely disrupted microbiota, the main function of the HilD regulon seems to reside in minimizing the costs associated with the triggering of gut inflammation by TTSS-1-, flagella-, and/or Sii-adhesin-dependent mucosa invasion. This host response promotes pathogen blooms in the gut lumen and thereby enhances transmission. The exact molecular nature of these costs is still being explored. In antibiotic pretreated mice, these costs entail reduced growth rates as observed ex vivo [37], death from innate host defenses encountered after tissue invasion [28,93], an altered sensitivity towards SCFA-mediated growth restriction [62,94], or a reduced energy level and an increased sensitivity of the regulon-expressing cells towards outer membrane disruption [95]. The sheer number of genes and phenotypes controlled by the HilD regulon [96] has been an obstacle in deciphering the cause of its expression costs. Our work presented here suggests that the gut microbiota are playing a critical role, by conditioning the gut milieu and thereby affect the relative costs and benefits of the HilD-regulon outputs. This explai [file pbio.3002253.s003.tif]

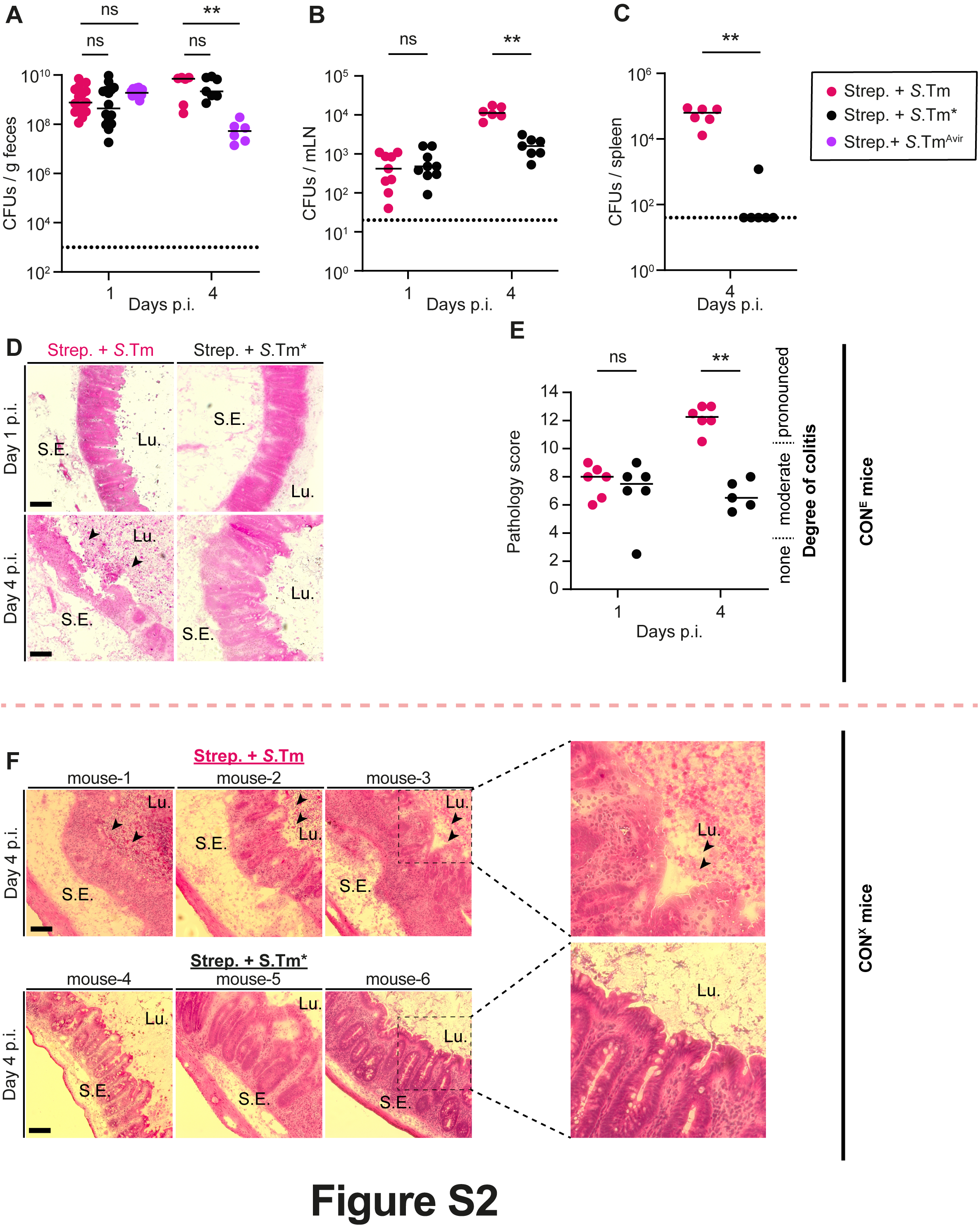

Supplement: S2 Fig — Streptomycin pretreated C57BL/6 (CONE) were infected with either wild-type S.Typhimurium (pink; n = 15, n = 9 euthanized at day 1 p.i. and n = 6 at day 4 p.i.; 5 independent experiments) or S.Tm* (pink; n = 16, n = 9 euthanized at day 1 p.i. and n = 7 at day 4 p.i.; 5 independent experiments) or S.TmAvir (purple; n = 13, n = 7 euthanized at day 1 p.i., n = 6 at day 4 p.i.; 4 independent experiments) with total CFU of 5 × 10^7^. Of note, all the inoculums contained 2.5 × 10^6^ CFUs (1:20) of S.TmAvir (tsr- vs. tsr+) as a reporter for the type of inflammation [97]. (A) Fecal populations of S.Tm (n = 15 mice analyzed), S.Tm* (n = 16 mice analyzed), and S.TmAvir (n = 9 mice analyzed) were determined by selective plating at day 1 or day 4 p.i. (B) mLN counts of S.TmWT (n = 15 mice analyzed), S.Tm* (n = 16 mice analyzed), and S.TmAvir (n = 9 mice analyzed) at day 1 or day 4 p.i. (C) Spleen counts of S.Tm (n = 6 mice analyzed) vs. S.Tm* (n = 6 mice analyzed) at day 4 p.i. (D) Representative images of hematoxylin and eosin (HE) stained cecal tissue sections from mice infected with S.Tm or S.Tm* at day 1 or day 4 p.i. Lu. = Lumen. S.E. = Submucosal edema. Black arrows indicate collapse of upper mucosa. Scale bar = 100 μm. (E) Histopathology scoring of the sections from panel D (n = 6 at day 1 and n = 6 for day 4 p.i. for S.Tm; n = 6 at day 1 and n = 5 for day 4 p.i. for S.Tm*). (F) Representative images of HE-stained cecum tissue sections (from 3 different mice) from streptomycin pretreated 129/SvEv (CONX) mice that were infected with either wild-type S.Typhimurium (S.Tm:S.TmhilD; 1,000:1 ratio) or S.Tm*(S.Tm*:S.Tm*hilD; 1,000:1 ratio) for 4 days; Lu. = Lumen. S.E. = Submucosal edema. Black arrows indicate collapse of upper mucosa. Scale bar = 100 μm. Dotted lines indicate the detection limit. Lines indicate the median. Two-tailed Mann–Whitney U tests were used to compare S.Tm to S.Tm* (p > 0.05 not significant (ns), p < 0.05 (*), p < 0.01 (**), p < 0.001 (***), p < 0.0001 (**** [file pbio.3002253.s004.tif]

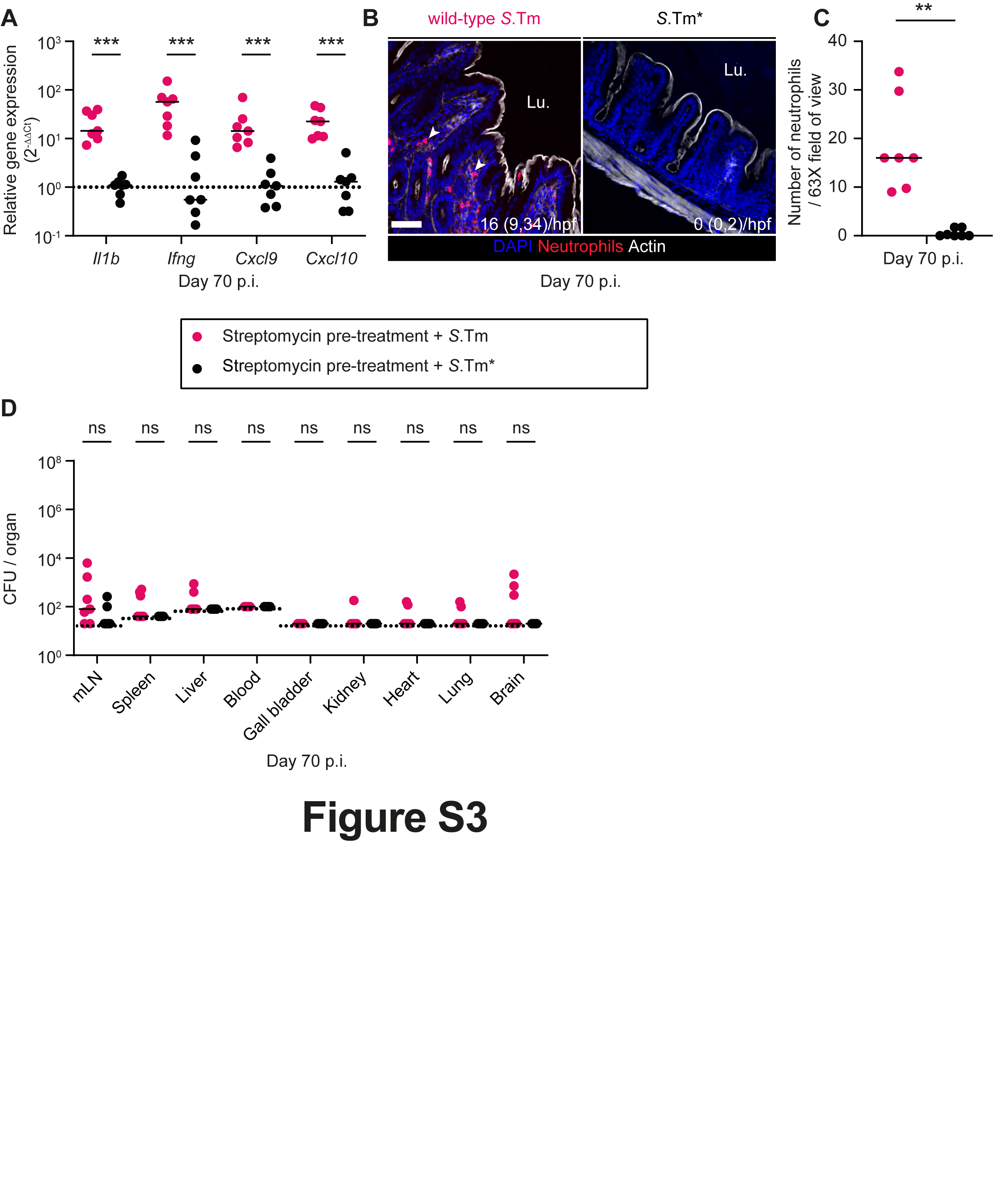

Supplement: S3 Fig — Typhimurium or S.Tm* (animals from Fig 1A–1D). (A) RTqPCR analysis of the pro-inflammatory gene expression in the cecum tissue of mice infected for 70 days with wild-type S. Typhimurium (pink; n = 7 mice analyzed) or S.Tm* (black; n = 7 mice analyzed). The results are plotted as fold-induction over the average of mice infected with S.Tm*. (B) Representative images of cecal sections at day 70 p.i. Nuclei (blue; DAPI), neutrophils (red; Ly6B.2), and actin (white; Phalloidin) are stained. Lu. = Lumen. White arrows point to neutrophils. Scale bar = 50 μm. HPF; high field of view. (C) Quantification of neutrophils in cecal sections. Each data point is the average of 5 fields of view (FOV) per mouse (n = 7 mice per group). Lines indicate the median. (D) Salmonella organ loads of mice euthanized on day 70 p.i. (n = 7 for both groups) with wild-type S. Typhimurium (n = 7) or S.Tm* (n = 7). Lines indicate the median. mLN = mesenteric lymph node. Blood is reported as CFU per ml. Lines indicate the median. Two-tailed Mann–Whitney U tests were used to compare the data between the groups infected with wild-type S. Typhimurium and S.Tm* (p ≥ 0.05 not significant (ns), p < 0.05 (*), p < 0.01 (**), p < 0.001 (***)). These data show that innate and adaptive immunity are controlling the infection by both strains about equally well by day 70 p.i. Source data can be found in S1 Data file. (TIF) [file pbio.3002253.s005.tif]

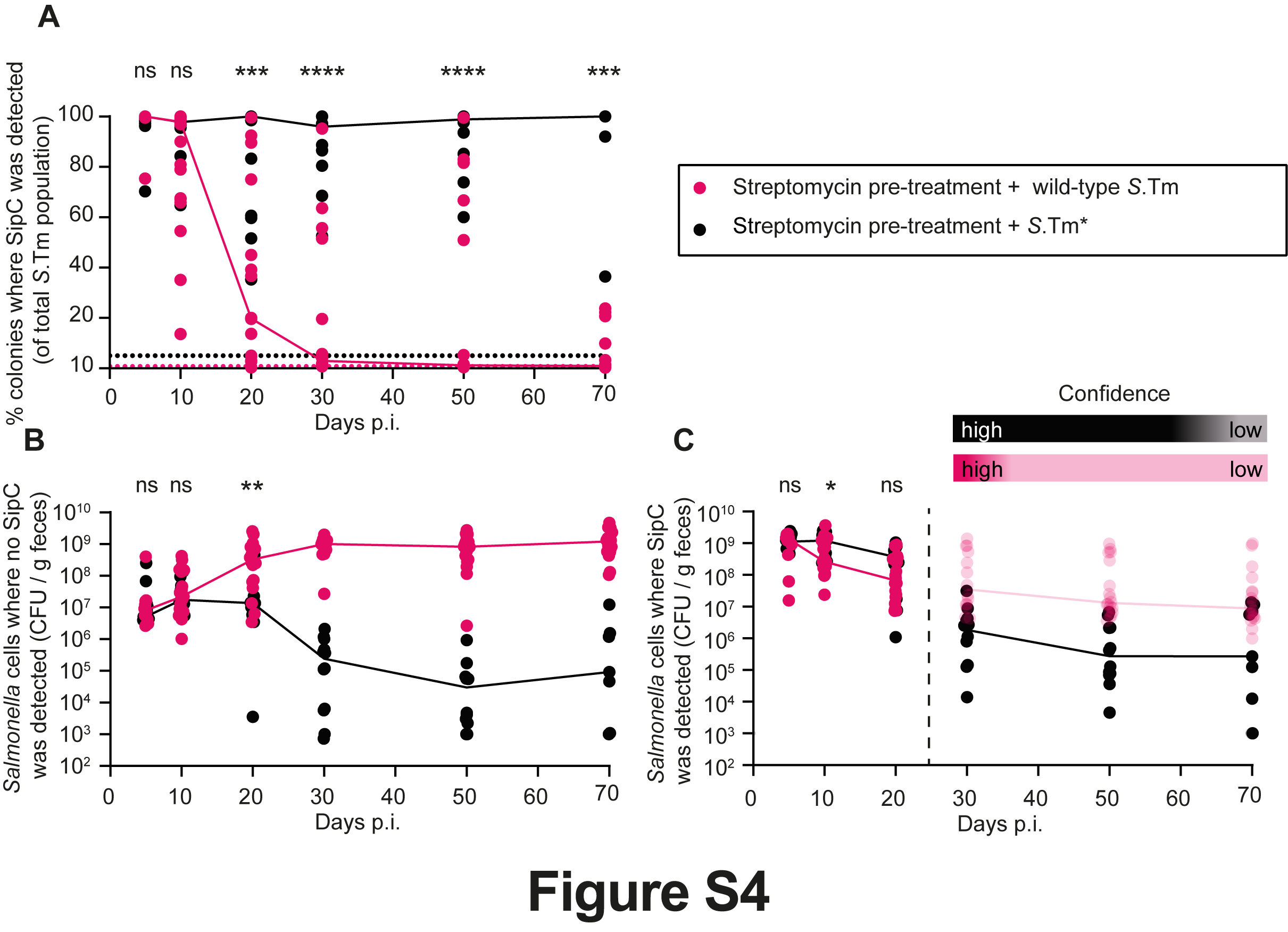

Supplement: S4 Fig — (A) Fraction of the fecal Salmonella population which yielded colonies where SipC was detected. (B) Estimate of the total size of the Salmonella population which yielded colonies where no SipC was detected. (C) Estimate of the total size of the Salmonella population which yielded colonies where SipC was detected. (A–C) The black dotted line indicates the conservative detection limit. Colored lines connect medians. Transparent pink dots (at >20 days p.i.) indicate that the calculation of the absolute sizes of the respective Salmonella populations is not very reliable as the majority of mice have >95% clones where no SipC was detected. Two-tailed Mann–Whitney U tests were used to compare wild-type S.Typhimurium to S.Tm* infected groups (p ≥ 0.05 not significant (ns), p < 0.01 (**), p < 0.001 (***), p < 0.0001 (****)). Interpretation: During the first 20 days, S.Tm*-infected mice shed more pathogen cells capable of SipC expression in their feces than the mice infected with wild-type S. Typhimurium (S4C Fig; 9.5 × 10^8^ CFU per g feces per day vs. 6.5 × 10^8^ CFU per g feces per day; average over 20 days). After 20 days of infection with wild-type S. Typhimurium, clones capable of expressing SipC were rarely detected in the feces, which prevented a reasonable estimation of the total fecal pathogen loads that remained capable of expressing SipC between days 20–70 p.i. In contrast, S.Tm*-infected mice shed low but confidently detectable loads of clones that remained capable of expressing SipC (>104 CFU/g feces) until day 70 p.i. (S4C Fig). Thus, over the course of 70 days, S.Tm*-infected mice shed higher loads of clones capable of SipC expression (approximately >1.5-fold) and lower loads of clones incapable of SipC expression (approximately 104-fold less) in their feces compared to wild-type S. Typhimurium-infected mice. Source data can be found in S1 Data file. Of note, the comparison of the total population sizes that yield colonies with detectable SipC expression is not in [file pbio.3002253.s006.tif]

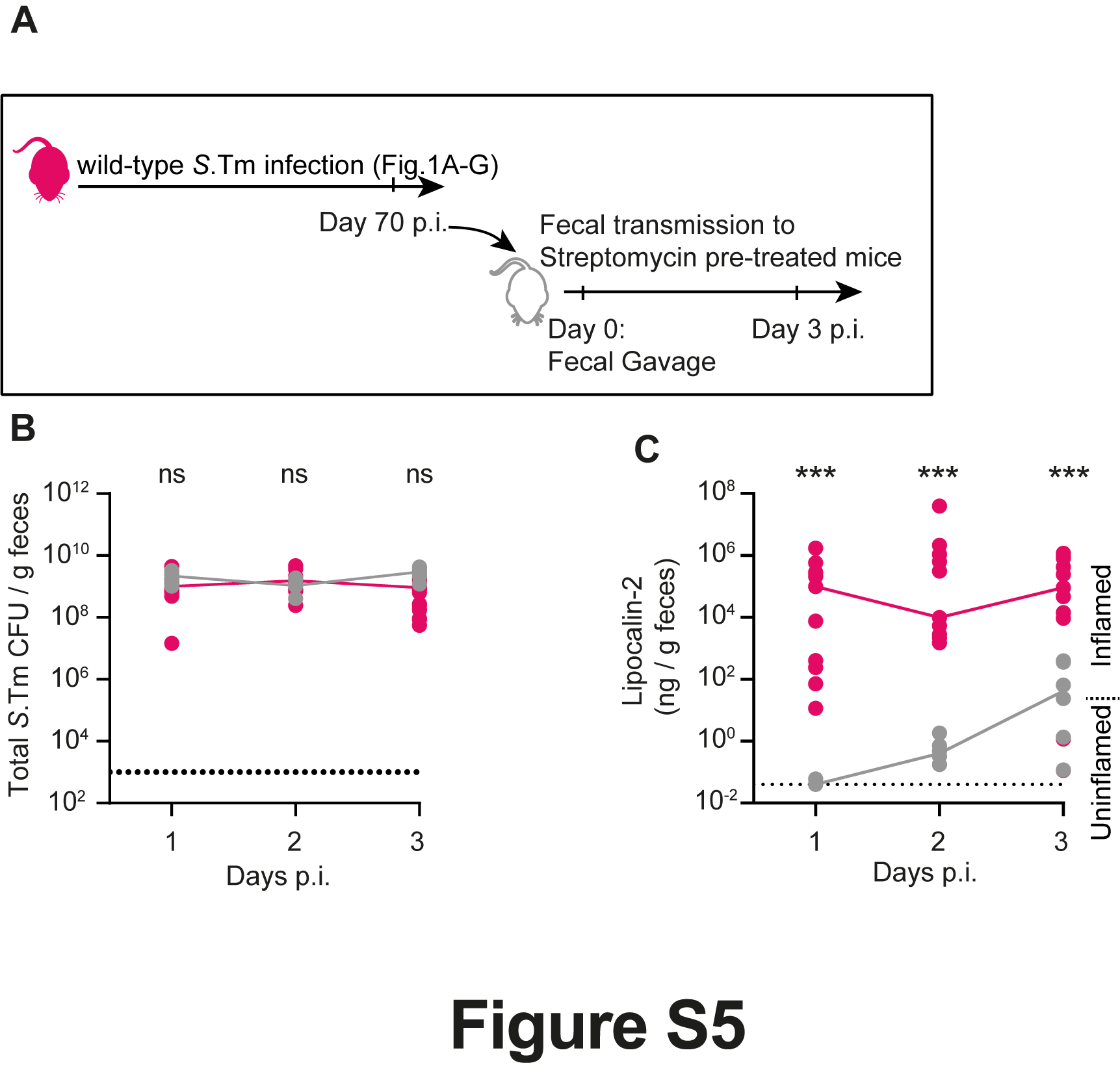

Supplement: S5 Fig — Typhimurium (from mice in Fig 1B–1D). (A) Experimental scheme. Fecal suspensions from mice in Fig 1A–1D (CONX 70 days after infection with wild-type S. Typhimurium) were transferred into streptomycin pretreated CONX mice (n = 6 mice; gray; 100 μl of 1 ml fecal suspension in PBS, by gavage). This provided us with a means to assess virulence after transmission. In contrast to the transmission by co-housing, the gavage of fecal suspensions allowed a more precise timing and dosing of the infection. The infection dynamics were compared to the original infection with wild-type S. Typhimurium in Fig 1A–1D at days 1–3 p.i. (n = 19 mice; pink). (B) Fecal Salmonella populations were enumerated by selective plating. (C) Lipocalin-2 concentrations in the feces, as determined by ELISA. Colored lines connect medians between time points. Dotted lines indicate the detection limits. Two-tailed Mann–Whitney U tests were used to compare the 2 groups at each time points (p ≥ 0.05 not significant (ns), p < 0.05 (*), p < 0.001 (***)). Source data can be found in S1 Data file. It should be noted that the experimental group developed gut Inflammation much later (days 3–4 p.i.), than the mice infected with the original wild-type S. Typhimurium strain (day 1–2 p.i.). This is consistent with a loss of TTSS-1 inflicted gut inflammation by the evolved Salmonella population. We hypothesize that the residual capacity to elicit enteropathy by this evolved population is attributable to TTSS-2-mediated pathogen growth in the gut tissue, which can elicit a delayed form of enteropathy in streptomycin pretreated mice (that is termed “alternative pathway”; [22]). (TIF) [file pbio.3002253.s007.tif]

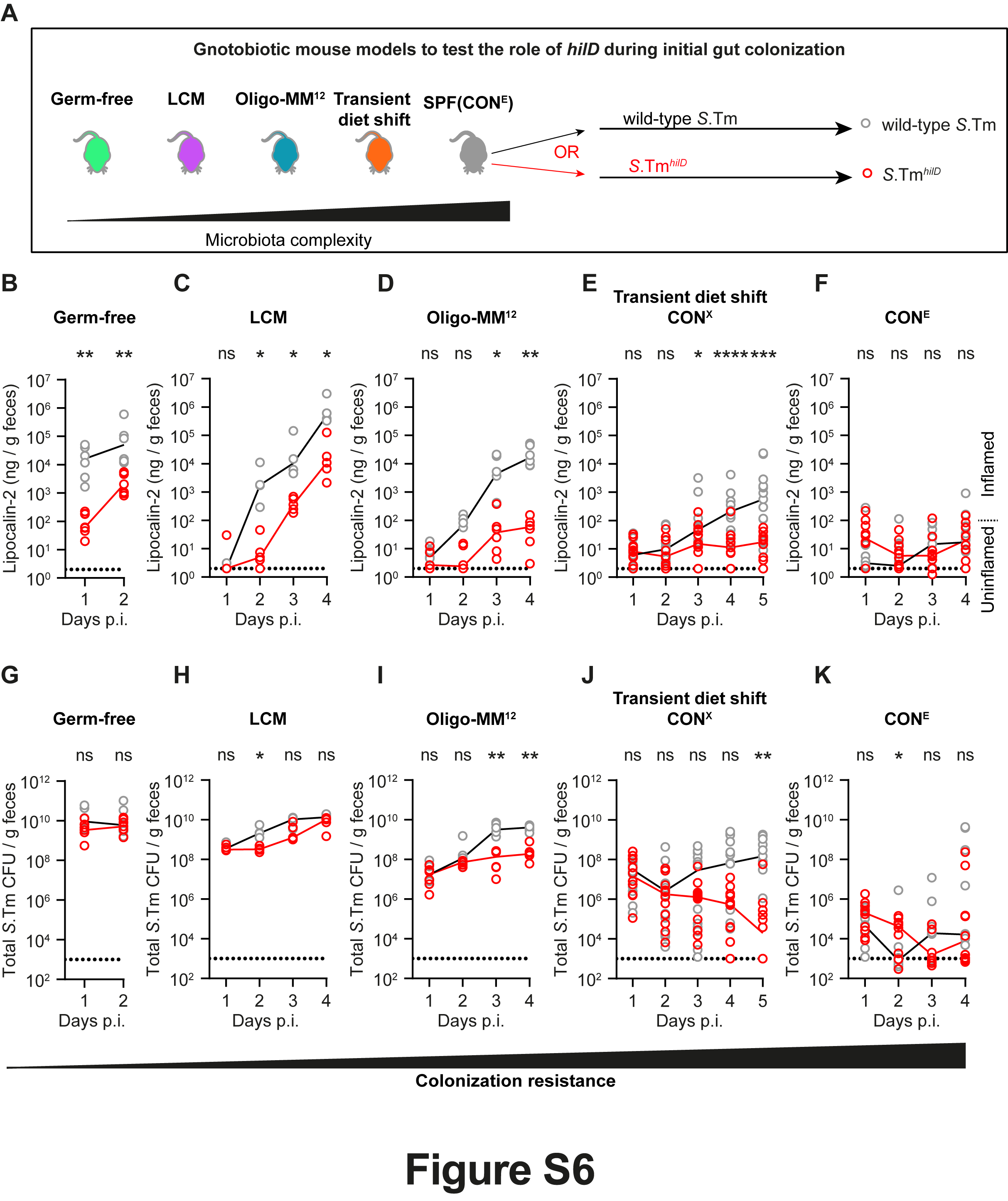

Supplement: S6 Fig — Typhimurium and S.TmhilD in mouse models conferring different degrees of CR (extends evidence related to Fig 2). (A) Experimental scheme. Germ-free mice confer no CR, can be infected via the orogastric route (this does not require any type of pretreatment) and wild-type S. Typhimurium will bloom up to carrying capacity (109–1010 CFU/g feces) and to elicit pronounced enteropathy within 12 h of infection [4,44]. LCM mice harbor a microbiota consortium composed of 8 strains and confer an intermediate level of CR. Upon orogastric infection, wild-type S. Typhimurium takes 3–4 days to grow to carrying capacity and 2–4 days to elicit pronounced enteropathy [4,46,47]. OligoMM12 mice harbor a defined microbiota composed of 12 representative microbiota strains [57]. They confer an intermediate level of CR. Upon orogastric infection, wild-type S. Typhimurium takes 3–4 days to grow to carrying capacity and to elicit pronounced enteropathy [4,25,57]. The complex SPF microbiota of CONE mice confers strong CR and prevents in most animals gut luminal growth of wild-type S. Typhimurium and of attenuated Salmonella mutants, alike. Thus, after orogastric infection, the Salmonella loads in the feces remain low in most CONE mice (typically <106 CFU/g feces) and no enteropathy develops within the first 5 days of infection (similar to CONX mice). For comparison, we also re-plot the data for CONX (from Fig 2) that were shifted from their normal plant based diet to a high-fat diet for the day before the infection. The 24 h exposure to high-fat diet reduces the degree of CR in the CONE mice so that wild-type S. Typhimurium can grow up to densities of ≈108 CFU/g gut-luminal content and elicits enteropathy by day 3–4 in most animals [4,25]. (B–K) Groups of the indicated mice were infected either with wild-type S. Typhimurium (5 × 10^7^ CFU, by gavage; gray circles; n = 6 for GF, n = 4 for LCM, and n = 6 for Oligo-MM12; n = 10 for CONE; n = 13 for transient diet shift) or with an isogenic hilD mu [file pbio.3002253.s008.tif]

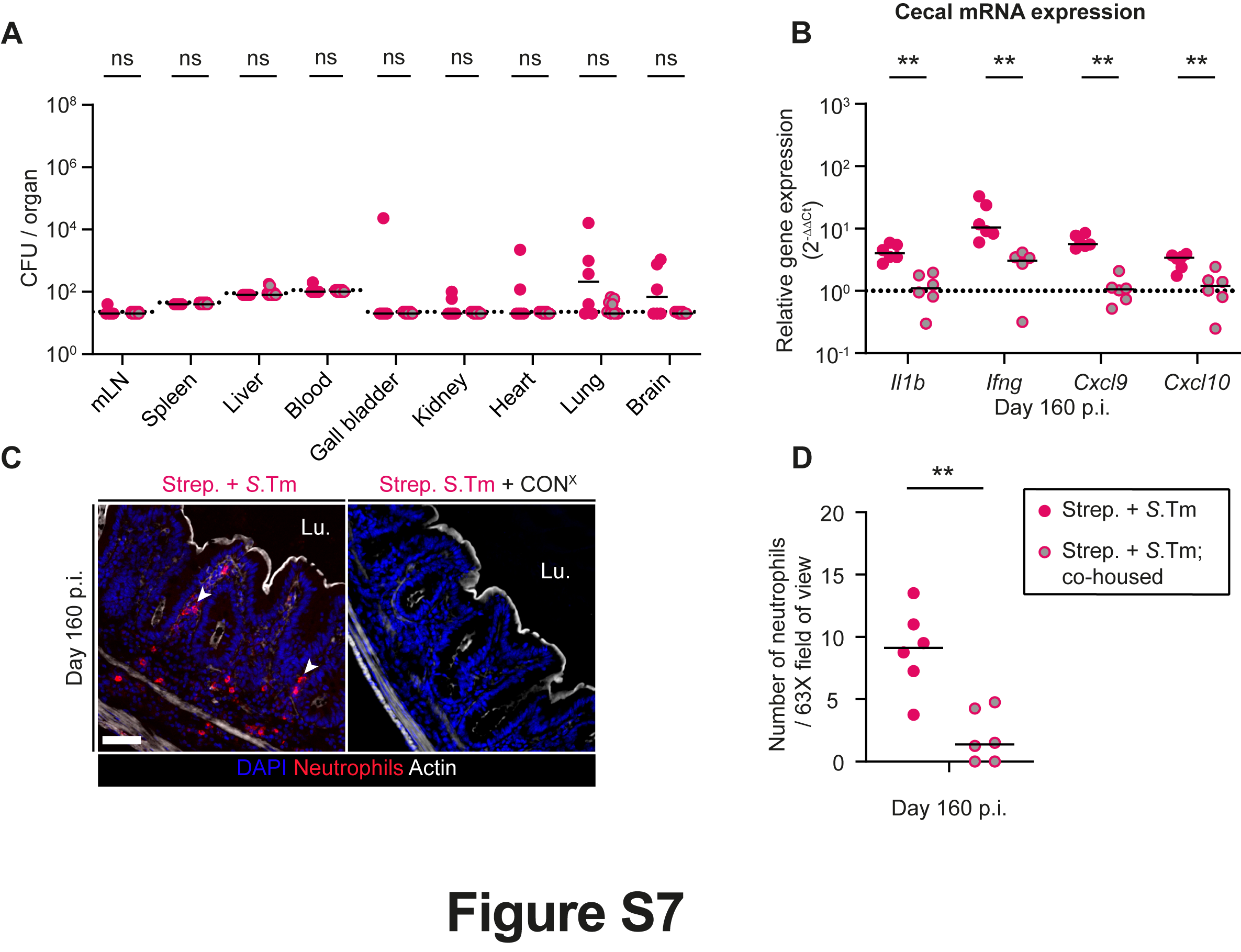

Supplement: S7 Fig — Mice had been infected with wild-type S. Typhimurium (as described in Fig 3) and co-housed (red circles with gray filling; n = 6 mice per group) or not (red filled circles; n = 6 mice per group) with an unperturbed CONX donor mouse from day 70 p.i. on. (A) Organ loads at day 160 p.i. (n = 6 mice per group). Lines indicate the median. mLN = mesenteric lymph node. Blood is reported as CFU per ml. (B) Pro-inflammatory gene expression analysis at day 160 p.i. (n = 6 mice per group). The results are plotted as fold induction from cecal tissue of mice w/o co-housing over the average of mice co-housed with a CONX donor mouse. (C) Representative immunofluorescence images of cecal tissue sections from mice of both groups at day 160 p.i. Nuclei (blue; DAPI), neutrophils (red; Ly6B.2), and actin (white; Phalloidin) are stained. Lu. = Lumen. White arrows indicate neutrophil recruitment to the cecal epithelium. Scale bar = 50 μm. (D) Quantification of neutrophils in cecal tissue sections as shown in panel C (n = 6 mice per group). (A–D) Two-tailed Mann–Whitney U tests were used to compare S.Tm to S.Tm + CONX (p ≥ 0.05 not significant (ns), p < 0.05 (*), p < 0.01 (**)). Source data can be found in S1 Data file. (TIF) [file pbio.3002253.s009.tif]

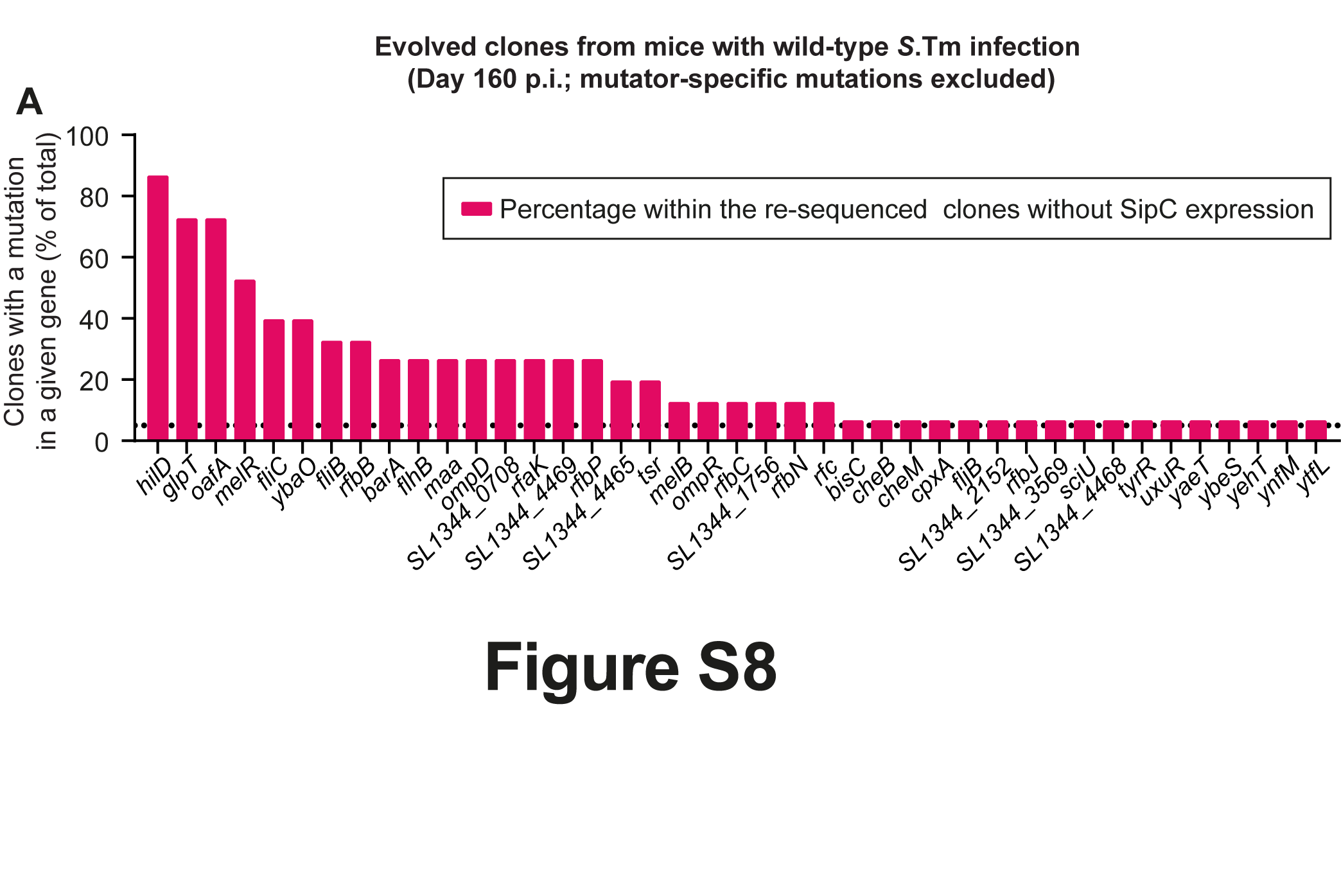

Supplement: S8 Fig — Typhimurium infected mice at day 160 p.i. (relates to Fig 3A–3D). Genome re-sequencing was performed on clones isolated from mice in Fig 2A–2E. A complete overview of the non-synonymous mutations is summarized in S3 Table. Genes mutated clones without detectable SipC expression were recovered from the control mice infected with wild-type S. Typhimurium 160 days without co-housing. Genes mutated only in mutS mutant mutator strains are excluded from this graph. Only non-synonymous mutations are shown. Genes are sorted according to the frequency of mutations found in the respective gene (n = 20 clones total; dotted line indicates the percentage that corresponds to a mutation in only 1 clone). hilD mutations were detected in 87% of all analyzed clones and the frequency of hilD mutations was even higher (and additional mutations accumulated) compared to the clones isolated at days 50–70 p.i. (S2 Table). Source data can be found in S1 Data file. (TIF) [file pbio.3002253.s010.tif]

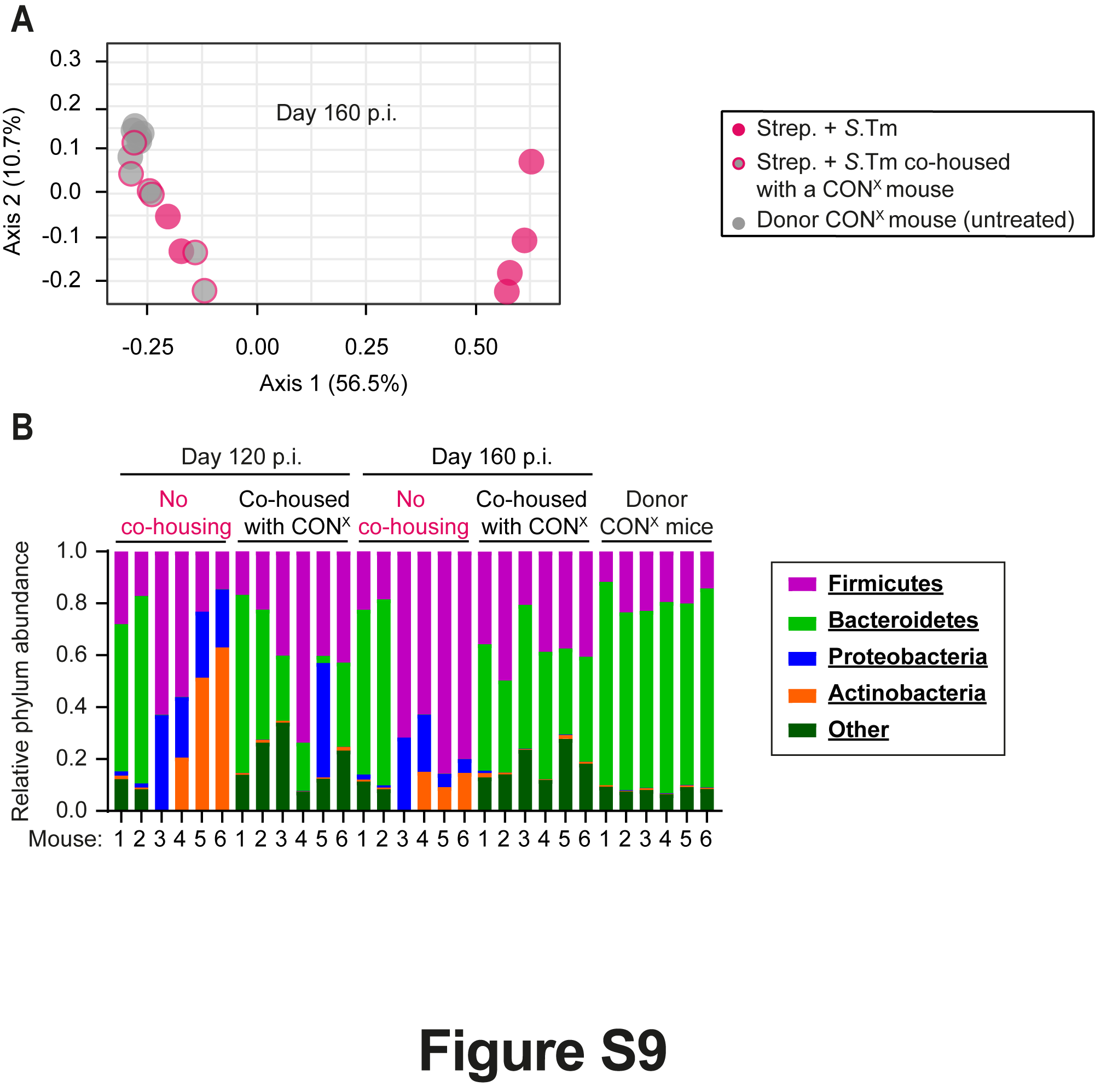

Supplement: S9 Fig — (A) Principal coordinate analysis based on Bray–Curtis dissimilarities between samples (after the square-root transformation of abundances). Data points represent individual mice at day 160 p.i. PERMANOVA R2 = 0.284 and p = 0.017 for day 120 p.i. and R2 = 0.4034 and p = 0.009. (B) 16S community sequencing analysis of fecal samples at day 120 and day 160 p.i. from control or co-housed mice (n = 6 fecal microbiotas were analyzed for each group). Source data can be found in S1 Data file. The bar plot shows the relative abundance of the dominant phyla. The four most abundant phyla are shown, with the rest of the community shown as “other.” (TIF) [file pbio.3002253.s011.tif]

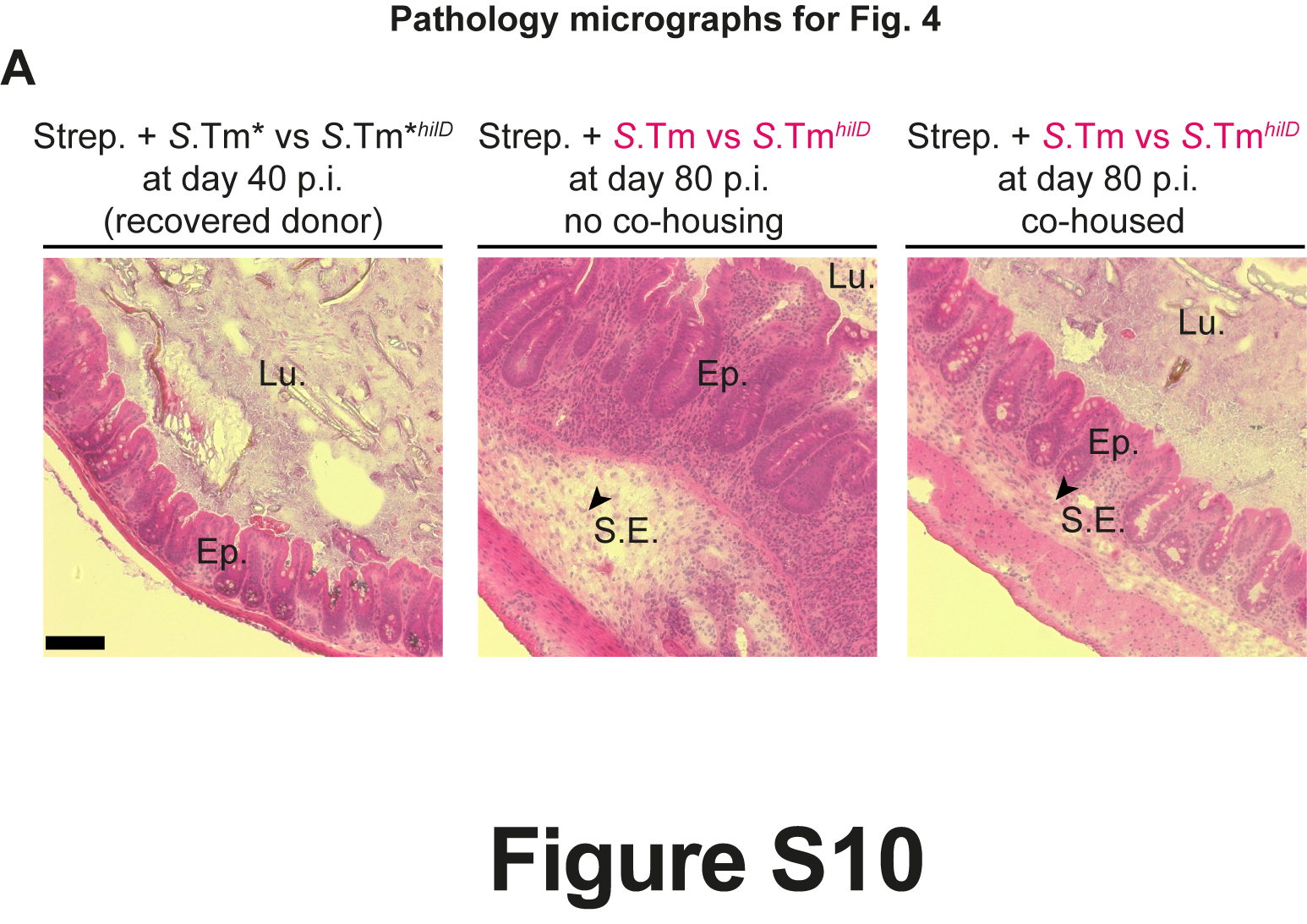

Supplement: S10 Fig — (A) Representative images of hematoxylin and eosin (HE) stained cecal tissue sections from mice infected with S.Tm*:S.Tm*hilD for 40 days, or S.Tm:S.TmhilD for 80 days, or infected with S.Tm:S.TmhilD for 40 days and co-housed with a recovered mouse for another 40 days Lu. = Lumen. S.E. = Submucosal edema. Black arrows indicate submucosal edema. Scale bar = 100 μm. (TIF) [file pbio.3002253.s012.tif]

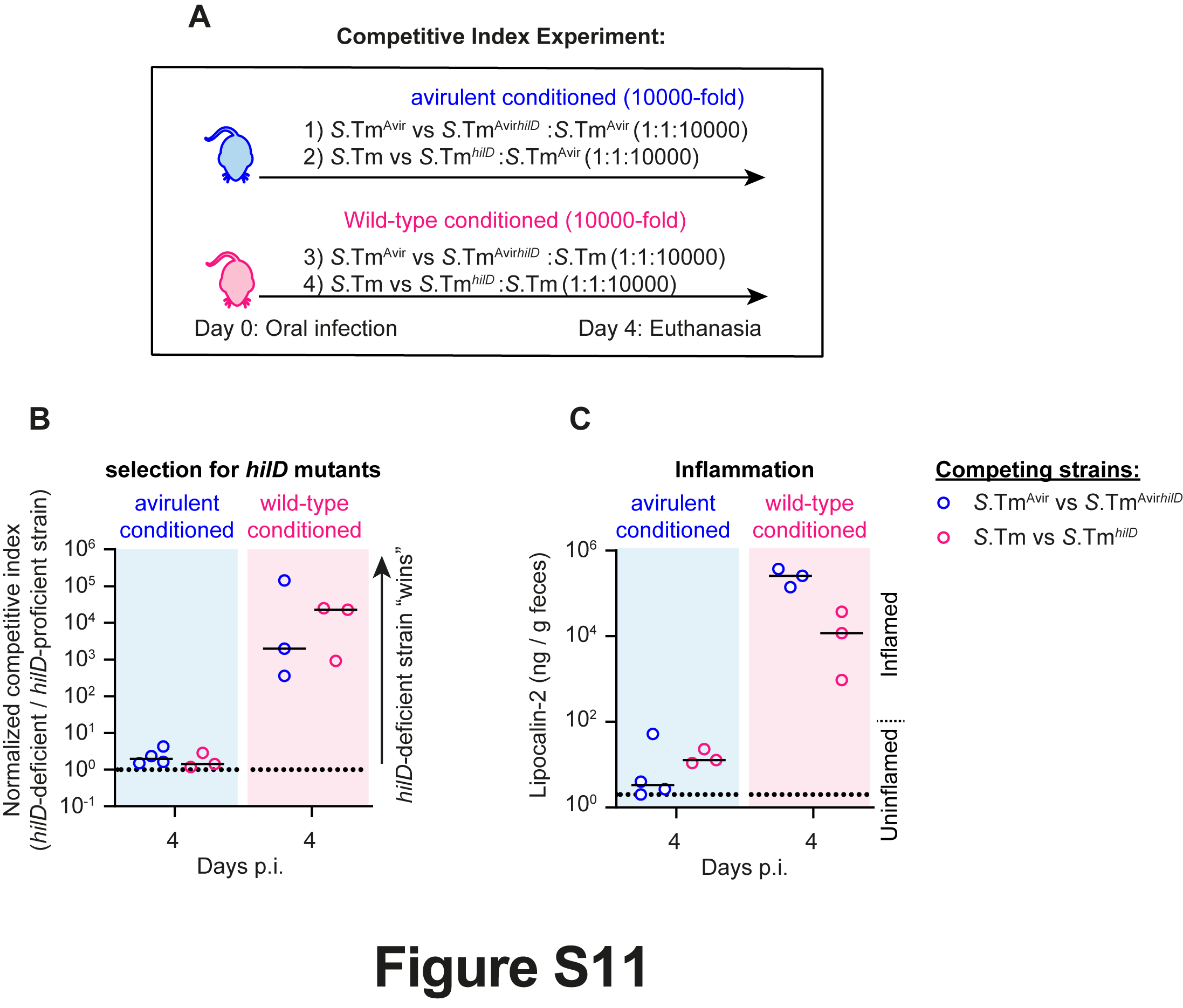

Supplement: S11 Fig — Typhimurium and this happens in invasive and non-invasive strain backgrounds. Streptomycin pretreated C57BL/6 mice harboring a complex specified pathogen free microbiota (termed CONE mice in this work) were infected with the following mixtures of Salmonella strains (5 × 10^7^ CFU, by gavage): (1) S.TmAvir:S.TmAvirhilD: S.TmAvir (1:1:10.000; n = 4 mice per group) (Avirulent conditioned); (2) S.Tm:S.TmhilD: S.TmAvir (1:1:10.000; n = 3 mice per group) (Avirulent conditioned); (3) S.TmAvir:S.TmAvirhilD: S.Tm (1:1:10.000; n = 3 mice per group) (wild-type conditioned); (4) S.Tm:S.TmhilD: S.Tm (1:1:10.000; n = 3 mice per group) (wild-type conditioned) for 4 days. (A) Experimental scheme. (B) The competitive index (C.I.), as calculated by the ratio of the isogenic hilD-deficient vs. hilD-proficient strain pairs. The dotted line indicates a C.I. of 1. (C) Lipocalin-2 ELISA was performed on feces to compare the level of gut inflammation between the different groups. The dotted line indicates the detection limit. The color code indicates the degree of gut inflammation ([Lcn2]>200–1,000 ng/g feces; mild gut inflammation, [Lcn2] = 1,000–1,000.000 ng/g feces, pronounced gut inflammation). Source data can be found in S1 Data file. (TIF) [file pbio.3002253.s013.tif]

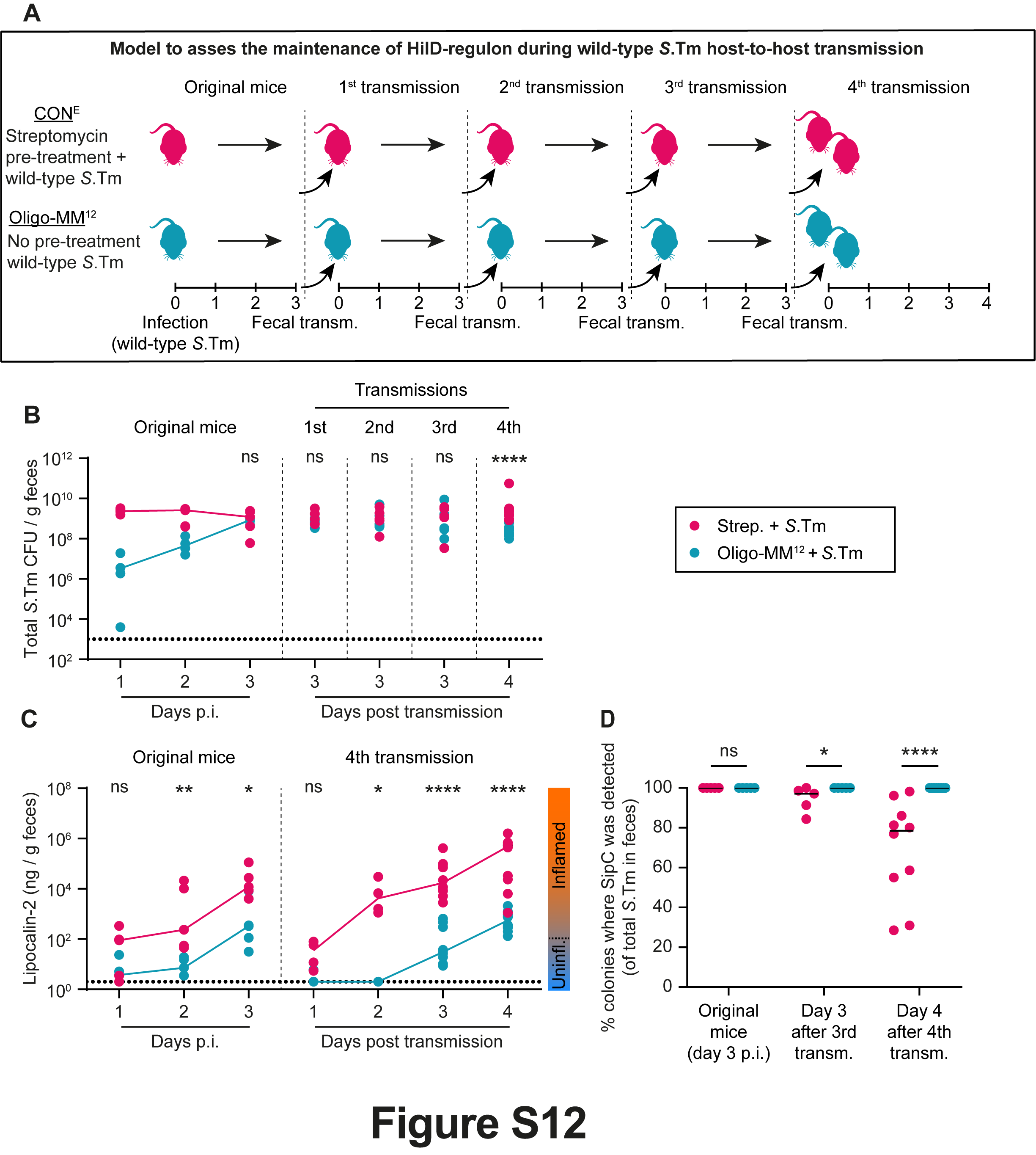

Supplement: S12 Fig — untreated OligoMM12. (A) Experimental scheme. Streptomycin pretreated CONE mice (strongly disrupted CR; n = 5 mice; 2 independent experiments; pink symbols) and Oligo-MM12 mice (intermediate CR; n = 5 mice; 2 independent experiments; blue symbols) were infected with the same wild-type S. Typhimurium strain (5 × 10^7^ CFU, by gavage). Fecal pellets were collected every third day, resuspended and used to infect the next group of mice (2 mice per 1 infected mouse pellet) by oral gavage at the day of collection. Mice from the last (fourth) transmission were infected for 4 days (n = 10 for each group). (B) Fecal Salmonella loads as determined using MacConkey plates with selective antibiotics. (C) Lipocalin-2 concentrations in the feces, as determined by ELISA. (D) Fraction of clones without detectable SipC, as determined by the colony protein blot assay. Two-tailed Mann–Whitney U tests were used to compare CONE + streptomycin to Oligo-MM12 at each time point (p ≥ 0.05 not significant (ns), p < 0.05 (*), p < 0.01 (**), p < 0.001 (***), p < 0.0001 (****)). Source data can be found in S1 Data file. Interpretation: In line with previous work, wild-type S. Typhimurium elicited enteropathy (as measured by lipocalin-2 ELISA) in both groups of mice. However, the disease had faster kinetics and was more pronounced in the streptomycin pretreated CONE mice than in the Oligo-MM12 mice develop moderate colitis in the first 4 days of the infection [85]. As we transmitted fecal suspensions into naive mice every third day (for 4 times), we ensured that OligoMM12 would not develop proceed to pronounced colitis (which would be expected to occur at day 4 p.i.). This design had 2 important advantages: We could achieve more vs. less pronounced colitis over the entire course of the experiment while using the exact same wild-type S. Typhimurium strain. And we could avoid life-threatening degrees of systemic pathogen spread, which would occur beyond days 5–6 after high-level gut colonization in s [file pbio.3002253.s014.tif]

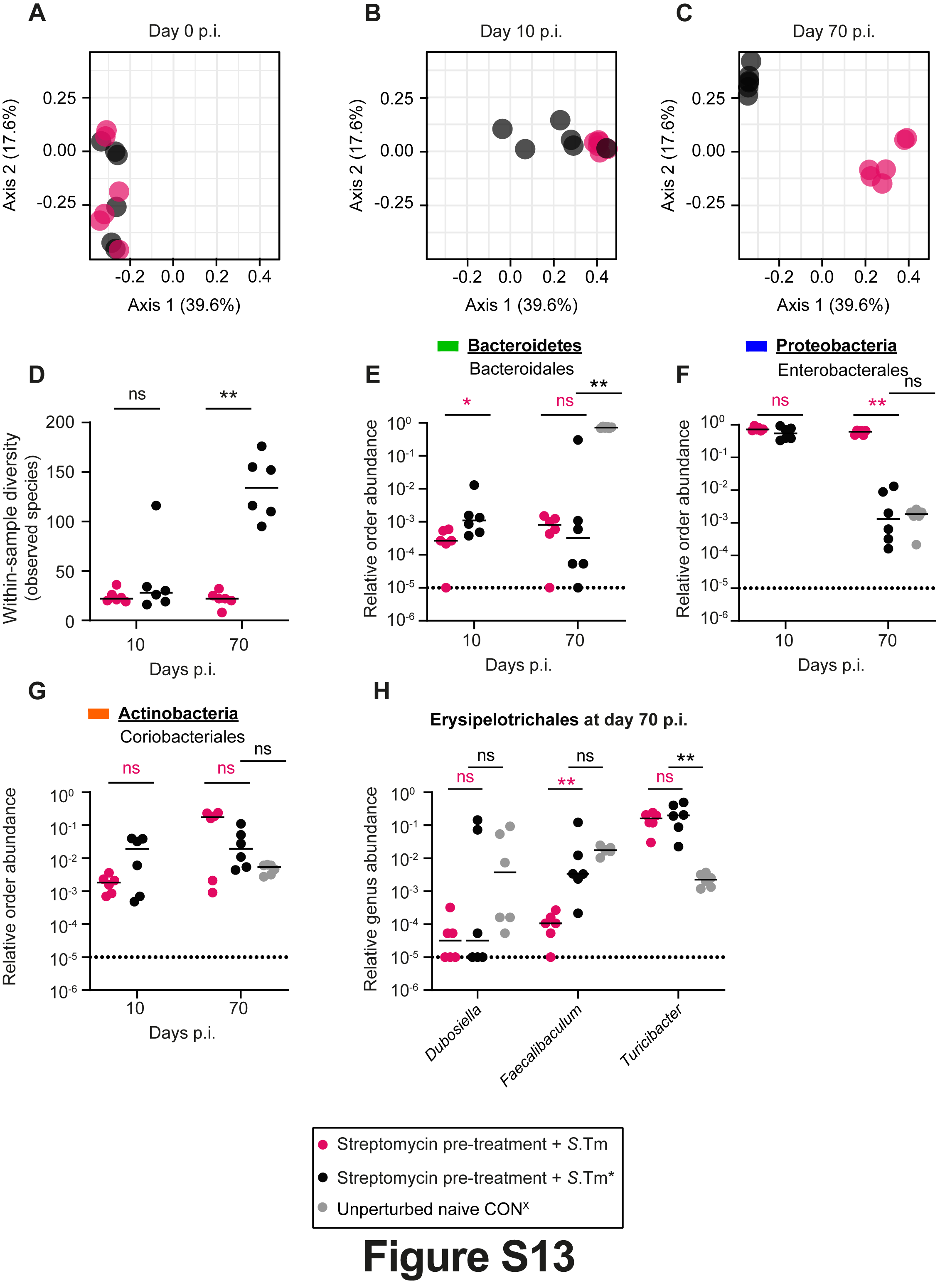

Supplement: S13 Fig — 16S community sequencing analysis of fecal samples at day 10 and day 70 p.i. from mice in Fig 1B–1D. (A–C) Principal coordinate analysis based on Bray–Curtis dissimilarities between samples (after the square-root transformation of abundances). Data points represent individual mice, and a colored border defines the grouping of data points within each sample group. PERMANOVA R2 = 0.067 and p = 0.589 for day 0 p.i., R2 = 0.397 and p = 0.0045 for day 10 p.i., and R2 = 0.660 and p = 0.002 for day 70 p.i. (D) Within-sample diversity as measured by the number of observed species. (pink circles: wild-type S. Typhimurium S.Tm; black circles: S.Tm*). (E–G) Relative order abundances compared at day 10 and 70 p.i. Fecal microbiota from unpertubed CONX mice served as reference point for the normal CONX microbiota. (E) Bacteroidales of the phylum Bacteroidetes. (F) Enterobacterales of the phylum Proteobacteria. (G) Coriobacterales of the phylum Actinobacteria. (H) Relative abundances of the most abundant genera belonging to the order Erysipelotrichales. Genera were compared at 70 p.i. between mice infected with wild-type S. Typhimurium vs. S.Tm*. Fecal microbiota from unpertubed CONX mice served as reference point for the normal CONX microbiota (gray circles). Dotted line indicates the detection limit. Lines indicate the median. Two-tailed Mann–Whitney U tests were used to compare wild-type S. Typhimurium to S.Tm*-infection or S.Tm*-infections to untreated CONX (p ≥ 0.05 not significant (ns), p < 0.05 (*), p < 0.01 (**), p < 0.001 (***), p < 0.0001 (****)). Source data can be found in S1 Data file. Interpretation: A striking dominance of genus Turicibacter in post-colitis gut was observed in both experimental groups in dependent of the level of gut inflammation, suggesting a superior adaptation of these bacteria to the inflamed gut. Besides, genus Faecalibaculum was differentially enriched only in the post-colitis gut of the S.Tm*-infected mice. Which could be interpreted as that [file pbio.3002253.s015.tif]

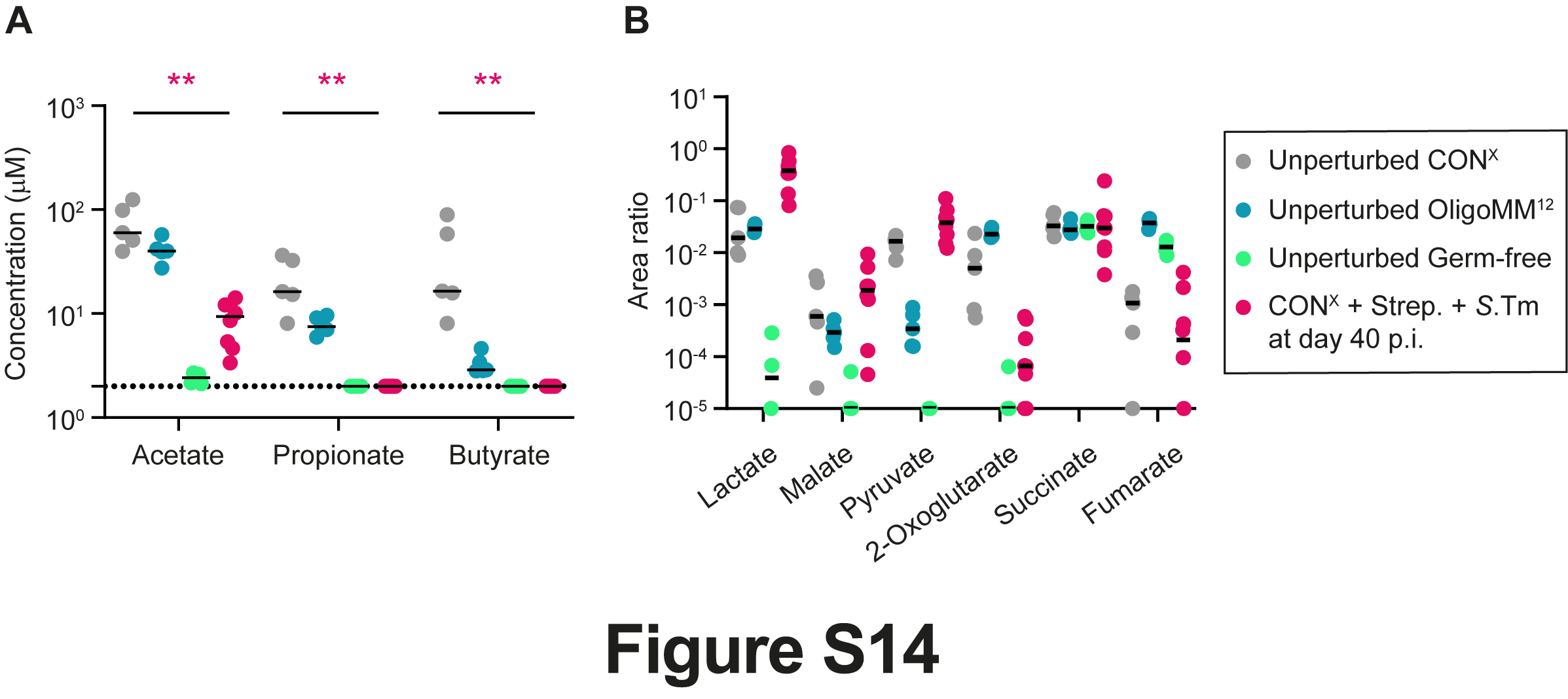

Supplement: S14 Fig — This analysis focused on the cecum content, as this is the site where most Salmonella and microbiota growth occurs. The cecum content from untreated CONX (n = 5 animals), OligoMM12 (n = 5 animals), and germ-free mice (n = 5 animals) was collected. Streptomycin pretreated CONX mice (n = 8 animals) were infected with a 106:1 mixture of wild-type S. Typhimurium and S.TmhilD and the cecal content was collected at day 40 p.i. The bacteria were removed by centrifugation and we collected the supernatants to quantify the metabolites accessible in the liquid content of the cecum lumen. Metabolite concentrations were determined by mass spectrometry with respect to internal standards for the SCFAs acetate, propionate, and butyrate or with respect to 13C labeled succinate for the other metabolites, such as lactate, malate, pyruvate, 2-oxoglutarate, succinate, and fumarate. (A) Concentrations of acetate, propionate, and butyrate. Dotted line indicates the limit of quantification (LOQ), values on the dotted line are below LOQ. (B) Relative abundance of lactate, malate, pyruvate, 2-Oxoglutarate, succinate, and fumarate as measured by the log2 transformed area ratio normalized to labeled succinate. Values on the X-axis are below the detection limit. Two-tailed Mann–Whitney U tests were used to compare untreated CONX mice to CONX mice at day 40 p.i. with wild-type S. Typhimurium (p ≥ 0.05 not significant (ns), p < 0.05 (*), p < 0.01 (**), p < 0.001 (***), p < 0.0001 (****)). Source data can be found in S1 Data file. Interpretation: The drop in the SCFA concentrations confirms that the wild-type S. Typhimurium infection disrupts the normal physiology of the CONX microbiota quite strongly and over long periods of time, as indicated by the day 40 p.i. data. (TIF) [file pbio.3002253.s016.tif]

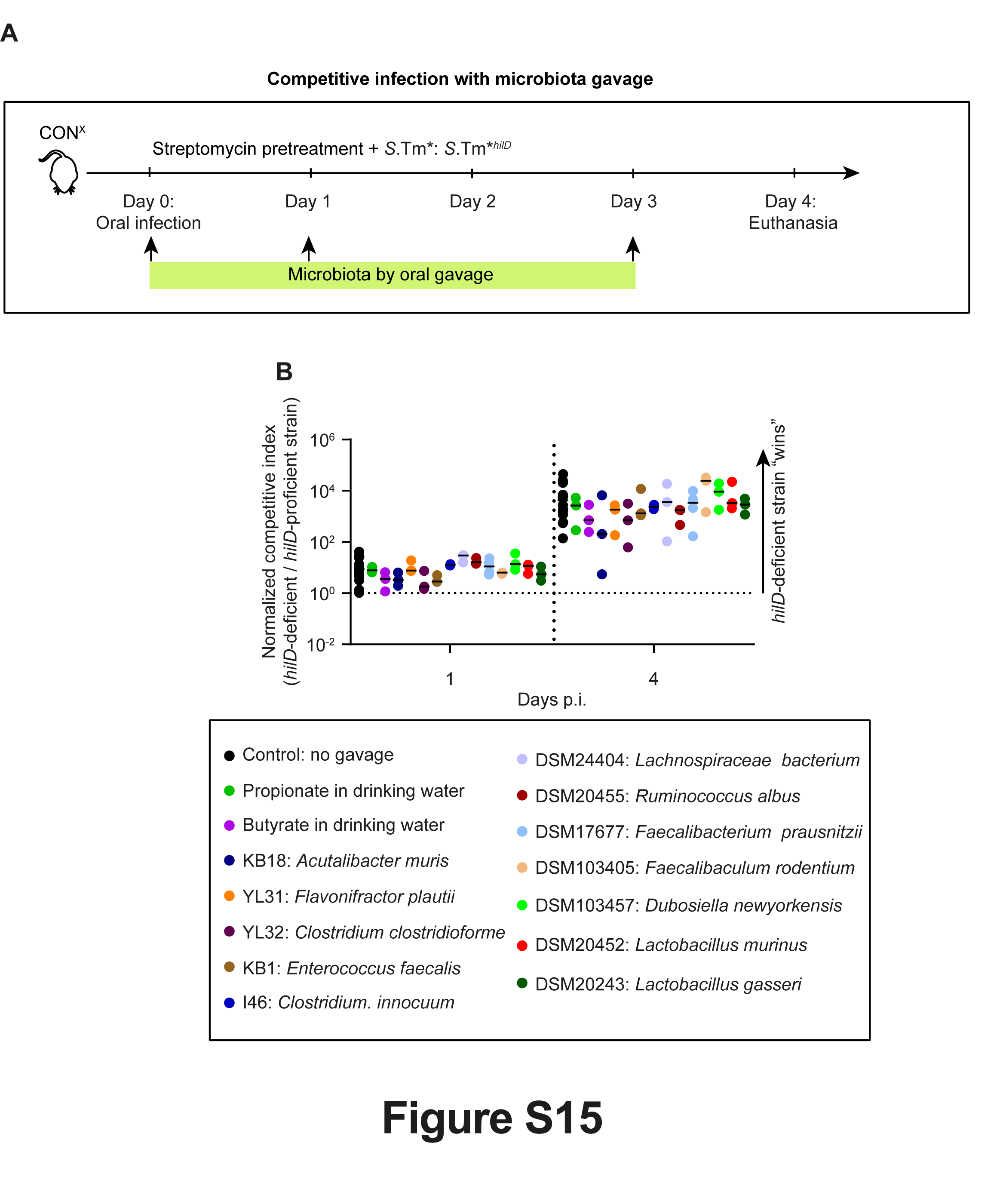

Supplement: S15 Fig — (A) Experimental scheme. (B) Streptomycin pretreated CONX mice (n = 35) were infected with a mixture of S.Tm* and isogenic hilD mutant (S.Tm*:S.Tm*hilD; 103:1 ratio) at day 0 and we analyzed pathogen gut colonization for 4 days. The first group remained in hygienic isolation (control; black circles; n = 14). The second and third group also remained under hygienic isolation and received drinking water supplemented with 25 mM Na-propionate or 50 mM Na-butyrate. The remaining groups were inoculated with glycerol stock of overnight cultures of the indicated strains on days 0, 1, 3 p.i. (ca. 5 × 10^5^ to 5 × 10^7^CFU, by gavage; n = 3 (except for DSM17677, n = 4)). The coloring indicates the tested microbiota strain. The normalized C.I. is plotted as the ratio of the isogenic hilD-deficient vs. hilD-proficient strain pairs, determined by selective plating. Source data can be found in S1 Data file. Interpretation: These results suggest that the selection against the rise of hilD mutants is a result of a combined effect of diverse microbiota members or associated metabolites. (TIF) [file pbio.3002253.s017.tif]

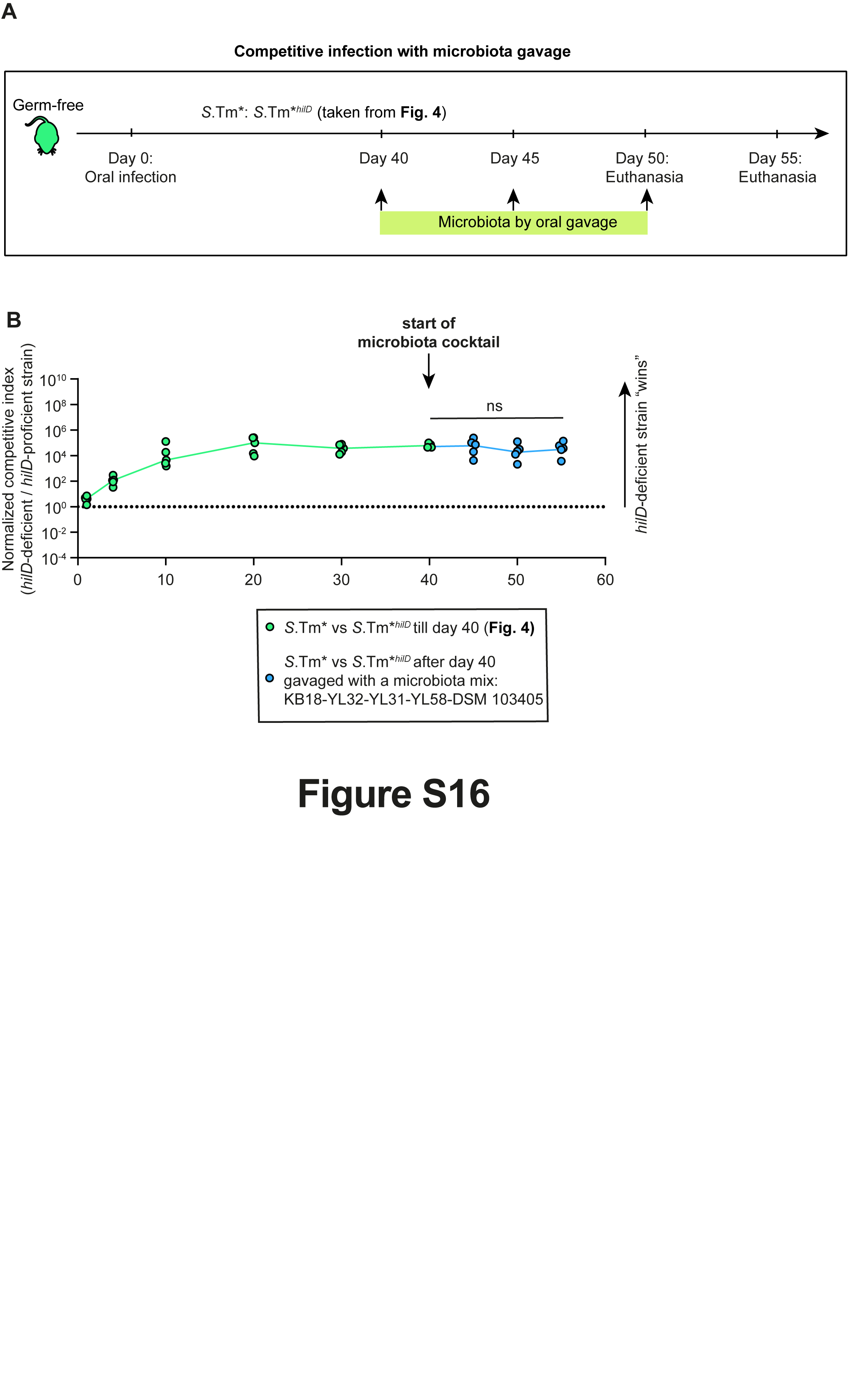

Supplement: S16 Fig — (A) Experimental scheme. Germ-free mice were taken from the end of the experiment shown in Fig 4. At this point, these germ-free mice had been infected for 40 days with a 100:1 mixture of S.Tm* and S.Tm* hilD (5 × 10^7^ CFU, by gavage; n = 5). On days 40, 45, and 50 after infection, the mice were inoculated, with glycerol stocks of o/n cultures from strains of OligoMM12 mice or from DSM collection (Cocktail A: KB18-YL32-YL31-YL58-DSM 103405; using an established protocol [57]). These microbiota strains were chosen to represent microbiota members that regrew by day 70 of infection with S.Tm* and which may therefore contribute to displacing mutant-dominated pathogen populations from the gut lumen. (B) Competitive index (C.I.) of S.Tm* hilD vs. S.Tm*, as determined using MacConkey plates with selective antibiotics. The C.I. is normalized to the inoculum. The dotted line indicates a C.I. of 1. The data for days 0–40 is re-plotted from Fig 4. Source data can be found in S1 Data file. Interpretation: The inoculation with the microbiota mixture had no detectable effect on the selection for or against S.Tm* hilD (compare days 20–40 to day 40–55 data). The reasons for this remain unclear. We speculate that this might be linked to our failure to identify the correct competitive microbiota strains, as our procedure to choose strains relied on 16S sequence information that does not provide any information on strain-specific traits that might be important for conditioning the gut-luminal milieu. (TIF) [file pbio.3002253.s018.tif]

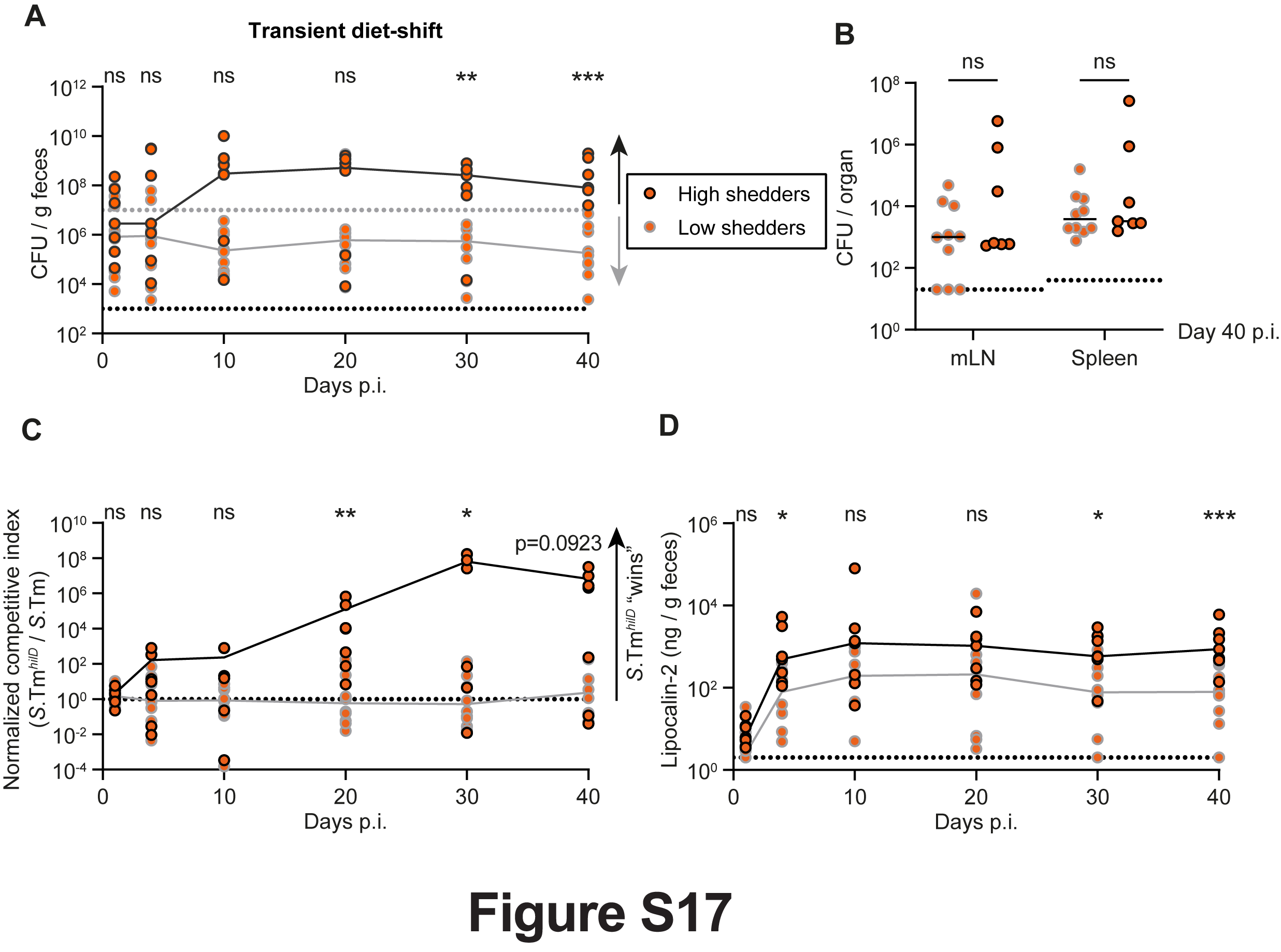

Supplement: S17 Fig — We re-analyzed the data from the experiment shown in Fig 7B–7D by splitting the mice into 2 subgroups based on the pathogen loads in the feces at day 10 p.i. High shedders (black symbols) had >107 CFU/g feces; low shedders (gray symbols) had <107 CFU/g feces at day 10 p.i. (A) Total Salmonella loads detected in the feces of high- and low shedders by selective plating. Dotted line indicates the detection limit. Black lines connect the medians. (B) Pathogen loads in the mLN and the spleen, as determined by plating. (C) The normalized competitive index (C.I.) high- and low shedders was determined by selective plating and is shown for wild-type S. Typhimurium versus S.TmhilD. The dotted line indicates a C.I. of 1. (D) An ELISA for fecal lipocalin-2 was used to compare gut inflammation between the 2 groups. Two-tailed Mann–Whitney U tests were used for statistical analysis (p ≥ 0.05 not significant (ns), p < 0.05 (*), p < 0.01 (**), p < 0.001 (***), p < 0.0001 (****)). Source data can be found in S1 Data file. Interpretation: This model nicely summarizes the trade-off between induction of gut inflammation and reduced virulence. While higher inflammation supports increased number of shedding pathogen cells (i.e., high shedders), this comes with the problem of losing virulence (increased hilD mutant accumulation). Thus, neither too high nor too low is optimal for S.Tm gut colonization. (TIF) [file pbio.3002253.s019.tif]

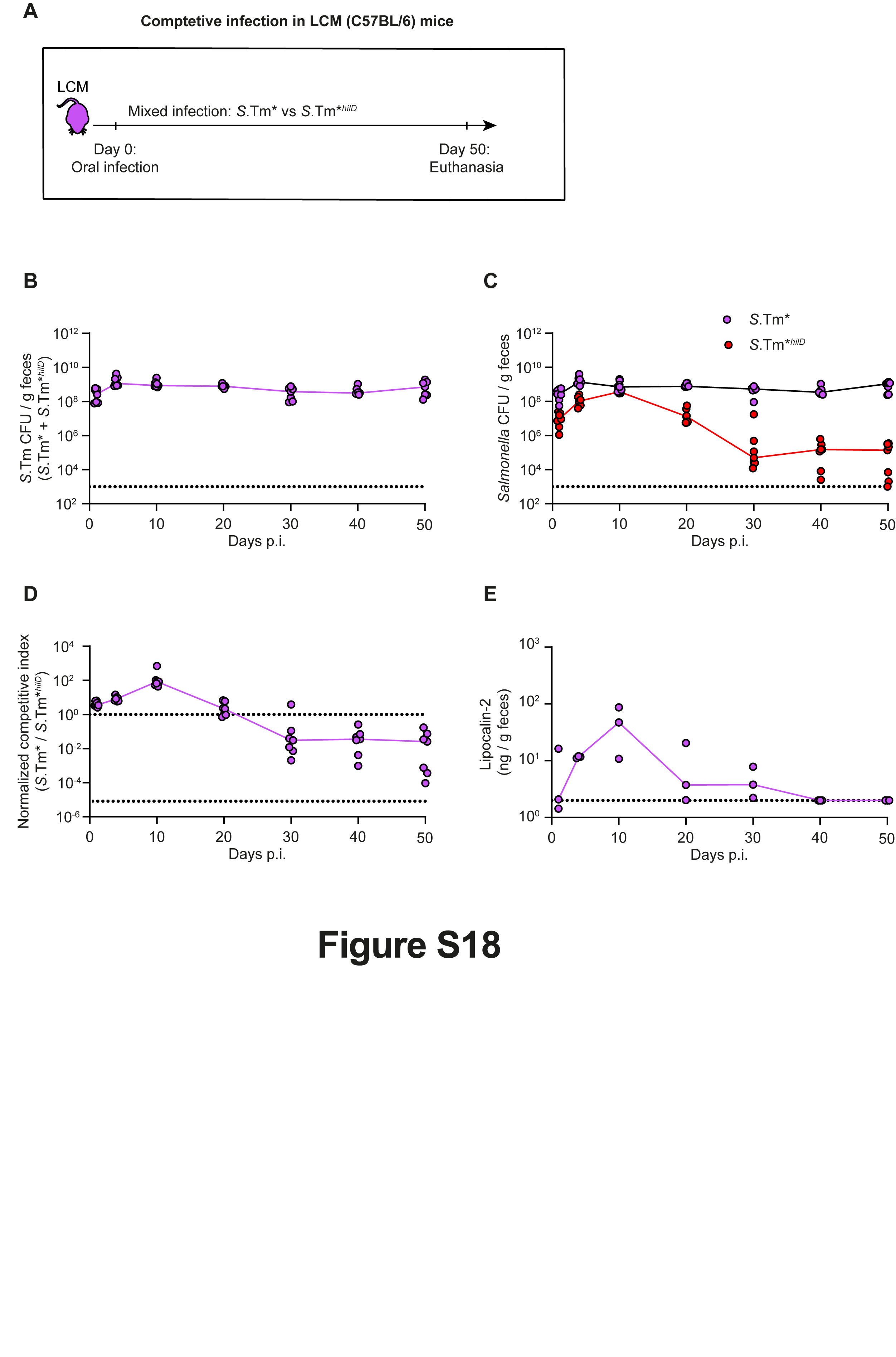

Supplement: S18 Fig — (A) Experimental scheme. (B–E) LCM mice were infected with a 100:1 mixture of S.Tm* and the isogenic hilD mutant S.Tm* hilD (5 × 10^7^ CFU, by gavage; n = 7 mice) for 40 days. (B) Total fecal Salmonella loads, as determined using MacConkey plates with selective antibiotics. (C) Fecal S.Tm* and S.Tm* hilD loads, as determined using MacConkey plates with selective antibiotics. (D) Competitive index (C.I.) of S.Tm* hilD vs. S.Tm*, as determined using MacConkey plates with selective antibiotics. The C.I. is normalized to the inoculum. The dotted line indicates a C.I. of 1. (E) Lipocalin-2 ELISA as performed on the feces. We analyzed a minimum n = 3 fecal pellets per time point. Dotted lines indicate the detection limit. Lines connect the median values at the days of analysis. Source data can be found in S1 Data file. Interpretation: In the LCM mice, we observed a selection for the hilD-deficient strain during the first 10 days of the infection. This is likely attributable to the inflamed gut-luminal milieu, which selected for the hilD mutant. Also, the inflammation has likely disturbed the LCM microbiota such that it cannot establish conditions selecting against the hilD mutant during these 10 days. The gut inflammation was apparently resolved by days 20–40, as indicated by the lipocalin-e ELISA data. In this period of the experiment, the selective advantage of the hilD-deficient strain was reversed. We hypothesize that this is attributable to the re-establishment of the LCM microbiota and that this accounts for the 10,000-fold selection of the hilD-proficient over the hilD-deficient strain (C.I. = 102 at day 10; versus C.I. = 10−2 at day 30). (TIF) [file pbio.3002253.s020.tif]
